# Design, synthesis and mechanistic study of N-4-Piperazinyl Butyryl Thiazolidinedione derivatives of ciprofloxacin with Anticancer Activity *via* Topoisomerase I/II inhibition

**DOI:** 10.1038/s41598-024-73793-y

**Published:** 2024-10-15

**Authors:** Hossameldin A. Aziz, Ahmed M. El-Saghier, Mohamed badr, Bakheet E. M. Elsadek, Gamal El-Din A. Abuo-Rahma, Mai E. Shoman

**Affiliations:** 1 Department of Pharmaceutical Chemistry, Faculty of Pharmacy, New Valley University, New Valley, 72511 Egypt; 2 Department of Medicinal Chemistry, Minia University, Minia, 61519 Egypt; 3 Department of Chemistry, Faculty of Science, Sohag University, Sohag, 82524 Egypt; 4 Department of Biochemistry, Faculty of Pharmacy, Menoufia University, Menoufia, Egypt; 5 Department of Biochemistry and Molecular Biology, Faculty of pharmacy, Assiut Branch, Al-Azhar University, Assiut, 71524 Egypt; 6 Department of Pharmaceutical Chemistry, Deraya University, New Minya, 61768 Minia Egypt; 7 Department of pharmaceutical chemistry, Deraya University, New Minia, 61768, Egypt

**Keywords:** Fluoroquinolones, Thiazolidinedione-2,4-dione, Anti-cancer, Topoisomerase inhibitors, Drug discovery, Medicinal chemistry

## Abstract

A new group of thiazolidine-2,4-dione derivatives of ciprofloxacin having butyryl linker **3a-l** was synthesized *via* an alkylation of thiazolidine-2,4-diones with butyryl ciprofloxacin with yield range 48–77% andfully characterized by various spectroscopic and analytical tools. Anti-cancer screening outcomes indicated that **3a** and **3i** possess antiproliferative activities against human melanoma LOX IMVI cancer cell line with IC_50_ values of 26.7 ± 1.50 and 25.4 ± 1.43 µM, respectively, using doxorubicin and cisplatin as positive controls with an IC_50_ of 7.03 ± 0.40 and 5.07 ± 0.29 µM, respectively. Additionally, compound **3j** showed promising anticancer activity against human renal cancer A498 cell line with IC_50_ value of 33.9 ± 1.91 µM while doxorubicin and cisplatin showed IC_50_ values of 3.59 ± 0.20 and 7.92 ± 0.45, respectively. On the other hand, compound **3i** did not show considerable anti-bacterial activity against *S. aureus*, E. *coli and P. aeruginosa*,* a*nd only moderate activity against *K. pneumoniae* with only a tenth of the activity of ciprofloxacin, confirming the cytotoxicity observed. Mechanistically, compound **3i** inhibited both topoisomerase I and II with IC_50_ of 4.77 ± 0.26 and 15 ± 0.81 µM. Furthermore, it induced cell cycle arrest at S phase in melanoma LOX IMVI cells. Moreover, **3i** provoked substantial levels of early, late apoptosis and necrosis in melanoma LOX IMVI cell line comparable to that induced by doxorubicin. Furthermore, compound **3i** increased the expression level of active caspase-3 by 49 folds higher in LOX IMVI cell, increased protein expression level of Bax more than the control by 3 folds and inhibited PARP-1by 33% in LOX IMVI. All results were supported by theoretical docking studies on both tested enzymes confirming potential cytotoxicity for the synthesized hybrids.

**Supplementary Information:**

The online version contains supplementary material available at 10.1038/s41598-024-73793-y.

## Introduction

 Fluoroquinolones are one of the most commonly prescribed antibiotics today for a variety of bacterial diseases, including urinary tract, skin, bone, soft tissue, upper and lower respiratory tract infections, and community-acquired pneumonia^1,2^. In addition, they have a variety of distinct biological characteristics, including anti-tumor^3^, anti-tubercular^4^, anti-HIV^5^, anti-malarial^6^, and anti-Alzheimer activities. Fluoroquinolones’ ability to halt cellular DNA replication and its associated processes underlies all their activities. Fluoroquinolones target two vital bacterial enzymes; Topoisomerase IV and DNA Gyrase which are essential for DNA replication and thus explain their bactericidal activity^7,8^. Reports also indicate that fluoroquinolones can impede the activity of Topoisomerase I/II. Topoisomerase enzymes are important for cell proliferation, differentiation, and survival by controlling and changing the topology of DNA in all cells^9^. Function in tumor cells is typically greater than in normal cells due to the rapid growth of cancer cells^10^. Thus, Topoisomerase I/II could be an excellent target for developing novel inhibitors with anticancer potential, including fluoroquinolones^3^.

**Fig. 1:**
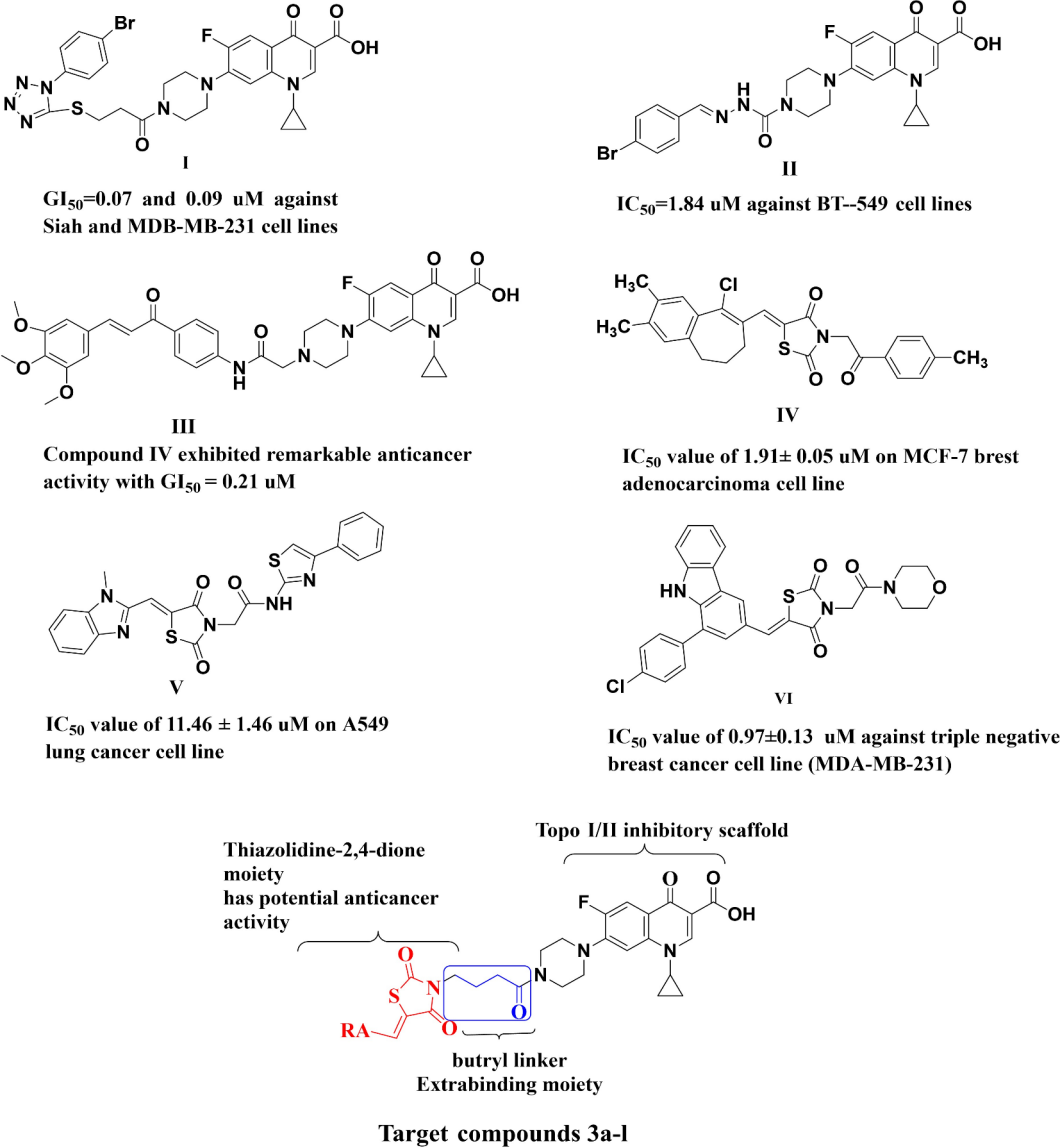
Structure of different ciprofloxacin hybrids (**I** and **II**,** III**), thiazolidine-2,4-derivatives (**IV**,** V** and **VI**) with anticancer activity and target compounds ciprofloxacin/ thiazolidine-2,4-dione hybrids **3a-l** designed for potential anticancer activities.

Additionally, as a potential anti-cancer agent, ciprofloxacin, at a concentration of 25 µg/mL, was reported to inhibit the proliferation of Jurkat cells^11^, while with doses higher than 80 µg/mL, it induced apoptosis for the same cell line^11^. These results elicited quinolone core as a critical component in designing new compounds that can fight cancer; one of the most lethal ailments worldwide^12,13^. Researchers employed various structural modifications and/or hybridization strategies to promote ciprofloxacin as a viable anticancer agent^14,15^. Reports emphasized the significance of the structure of the C-7 substitution on the fluoroquinolones’ ability to inhibit DNA topoisomerases I/II^16^. A major impact on both the physicochemical properties and biological activity was observed upon the introduction of substitution on the ciprofloxacin piperazine fragment^16^. Aromatic or hetero-aromatic scaffold carried on the piperazinyl nitrogen atom can potentiate anti-proliferative activity *via* inhibition of the mammalian enzymes topoisomerases I/II (For example: compounds I, II, and III, Fig. 1)^14,17,18^. In this context, various fluoroquinolone derivatives with N-4 piperazinyl alterations were introduced and exhibited potent cytotoxic activity *via* topoisomerase I/II inhibition, induction of apoptosis and cell cycle arrest (Fig. 1**)**^3,17,18^.

Nevertheless, thiazolidine-2,4-diones (TZD) constitute an important heterocyclic core system when combined with an aromatic moiety or other heterocyclic rings. It possesses numerous biological activities such as anti-diabetic^19^, anti-inflammatory^20^, anti-oxidant^21^, anti-microbial^22^, anti-tubercular^23^, and anticonvulsant^24^. In the literature, some TZD derivatives have promising anticancer activity *via* apoptosis induction^25^, cell cycle arrest^26^, promotion of cellular differentiation, and activation of peroxisome proliferator-activated receptors PPARγ which resulted in regulation of the transcription of selected target genes^25^. It’s interesting to note that some of the anti-tumor benefits of TZDs have been reported without PPAR- activation as induction of cellular acidosis *via* calcium storage depletion or inhibition of Na^+^/H^+^ exchanger, production of apoptotic factor reactive oxygen species (ROS), activation of mitogen activated protein kinase (MAPK), endoplasmic reticulum stress and proteasomal degradation^27,28^. Moreover, it was validated that the known TZDs, rosiglitazone potentiates the anticancer activity of gefitinib^28^. TZDs may provide a more important role in cancer treatment when combined with other pharmacophores as cancer is a complex multifactorial disease where its treatment requires combination therapy to be efficient. As a result, some hybrids containing thiazolidine-2,4-dione were synthesized and evaluated as anticancer agents (Compounds IV, V and VI, Fig. 1). Benzimidazole/thiazolidine-2,4-dione hybrids were synthesized and screened against cancer cell lines, with compound **V** showing promising anti-proliferative activity with IC_50_ of 11.46 ± 1.46 µM, half the inhibitory concentration against the A549 lung cancer cell line *via* induction of apoptosis and arresting cell cycle at G2/M phase^29^. Moreover, the in vitro anticancer activity of β-carboline-thiazolidinedione hybrids showed that compound **VI** exhibited the most potent activity against (MDA-MB-231) triple negative breast cancer cell line with an IC_50_ value of 0.97 ± 0.13 µM^30^. Additionally, benzosuberones /2,4-thiazolidenone hybrids exhibited anti-tumor activity towards breast adenocarcinoma (MDA-MB-231, MCF-7) with IC_50_ equal 13.39 ± 0.04 and 1.91 ± 0.03 µM, respectively (Compound IV, Fig. 1)^31^.

Based on the above-mentioned studies, the current research aimed at introducing the thiazolidine-2,4-one core into the *N*-4-piperazinyl moiety of ciprofloxacin *via* butyryl linker (Fig. 1) so that the two privileged pharmacophores were assembled in one molecular frame. This hybridization process attempts to potentiate the anti-cancer activity of ciprofloxacin and TZDs while improving the physiochemical properties of fluoroquinolones. The two merged pharmacophores in the current study were not studied before for the anti-cancer field. The anticancer activity was first screened at the NCI (National Cancer Institute, USA) against 60 cancer cell lines. The compound’s ability to affect the cell cycle and induce apoptosis was tested out to further validate their antiproliferative effect. Moreover, their ability to inhibit topoisomerase I/II was investigated to explore the mechanism of action, and docking studies were also done to explain the degree to which anticancer activity is related. Additionally, compounds **3a-l** were tested in comparison to parent ciprofloxacin against both Gram-positive strain and Gram-negative bacterial strains to shed insights into their antibacterial activity. While our group previously incorporated both thiazolidine-dione and ciprofloxacin in one entity to enhance anti-bacterial properties of ciprofloxacin, the current study is pioneering similar hybrids for potential synergism in anti-cancer activity. The designed compounds were hypothesized to offer activity mechanistically related to both merged scaffolds resulting in enhanced efficacy and unique physicochemical properties introducing such hybrids as potential candidates for anti-cancer drug discovery.

## Results and discussion

### Chemistry

**Fig. 2:**
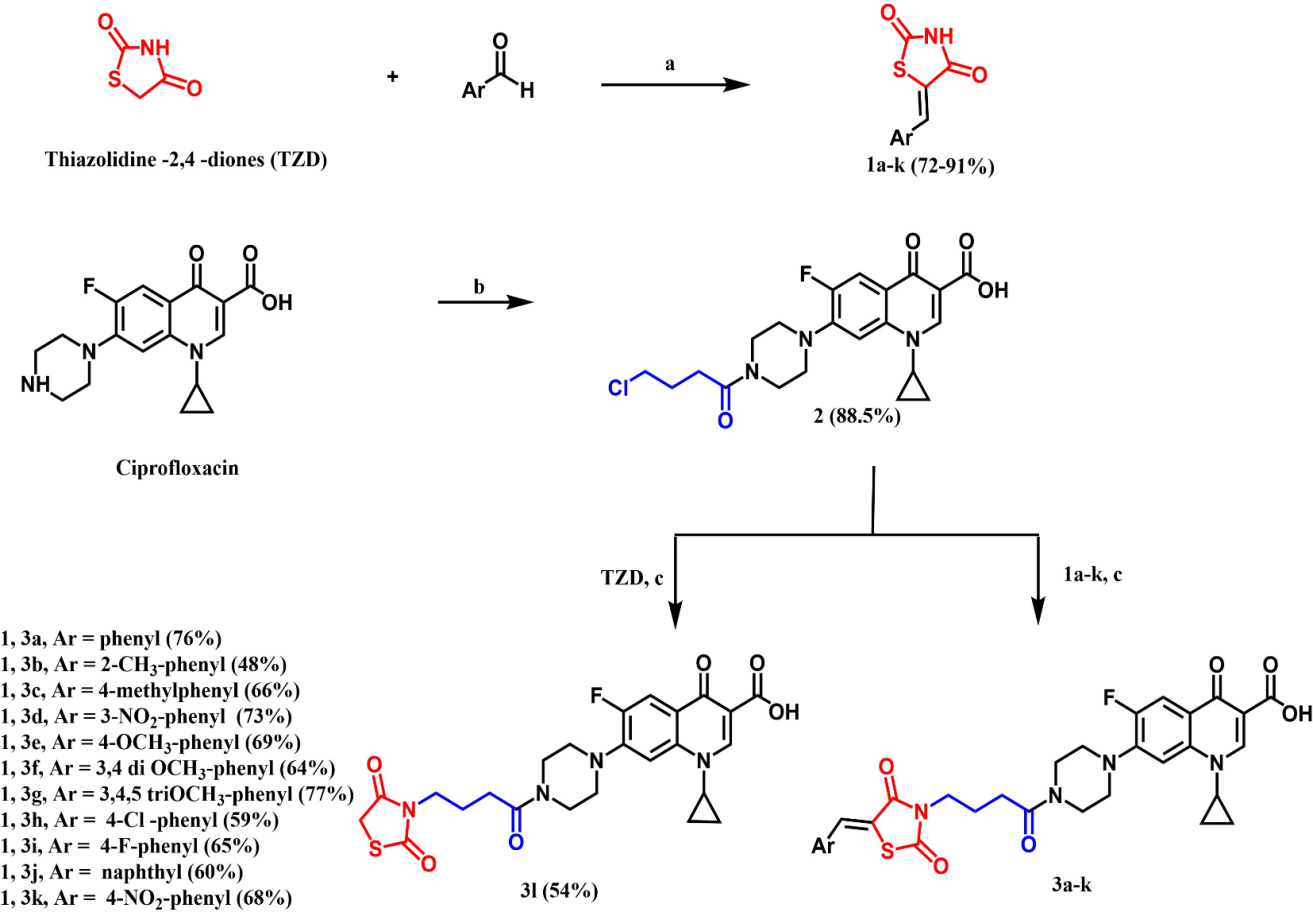
Synthesis of thiazolidine-2,4-dione derivatives **1a-k**, butyryl ciprofloxacin **2** and the designed compounds **3a-l**, **Reagents and conditions (a)** CH_3_COOH (glacial), CH_3_COONa (anhydrous); (**b**) chlorobutyryl chloride, CH_2_Cl_2_, K_2_CO_3_ and H_2_O; (**c**); KOH and DMF.

Target compounds **3a-l** were constructed as illustrated in Fig. 2. The key intermediate *N4*-butyryl ciprofloxacin **2** was synthesized by the reaction of ciprofloxacin with the acylating agent chlorobutyryl chloride in methylene chloride using a base as potassium carbonate^32^. The thiazolidine-2,4-dione core was synthesized as reported by merging chloroacetic acid with thiourea^33^. 5-Benzylidenethiazolidine-2,4-dione derivatives **1a-k** were then reached *via* Knoevenagel condensation of the appropriate aromatic aldehyde with thiazolidine-2,4-dione in glacial acetic acid in the presence of sodium acetate anhydrous^34^. Finally, synthesis of the target compounds **3a-l** was achieved with yield range (48–77%) *via* an alkylation of thiazolidine-2,4-diones **1a-k** or the unsubstituted, thiazolidine-2,4-dione with butyryl ciprofloxacin **2** in dimethyl formamide using a strong alkali such as KOH^34^. Compounds **3a-l** were characterized by different spectroscopic techniques, the presence of absorption corresponding to the five carbonyl groups is a characteristic feature in both IR and the ^13^C NMR spectra of these compounds. IR spectra showed the appearance of two carbonyls of thiazolidine-2,4-dione at 1735–1746 and 1647–1650 cm^−1^, in addition to another two carbonyls of ciprofloxacin scaffold and one carbonyl of the used linker. Similarly, ^13^C-NMR spectra of compounds **3a-l** showed five signals assigned for the five carbonyls in their expected chemical shifts. A characteristic downfield shift of the carbon for active methylene of compound unsubstituted, thiazolidine-2,4-dione **3 L** appeared at δ 42.95 ppm which upon condensation with different aldehydes appeared at δ 139.21-139.65 in addition to the characteristic pattern of ciprofloxacin^35^. ^1^H-NMR spectra showed the appearance of a new singlet mainly at 7.80–7.95 correspondent to the formed C*H* = C). Signal characteristics for the butyryl linker and ciprofloxacin scaffold appeared at their expected chemical shifts^35^.

### Pharmacology/biology

#### In vitro cytotoxicity screening

##### One dose anticancer screening of compounds 3a-l (NCI, USA)

**Fig. 3:**
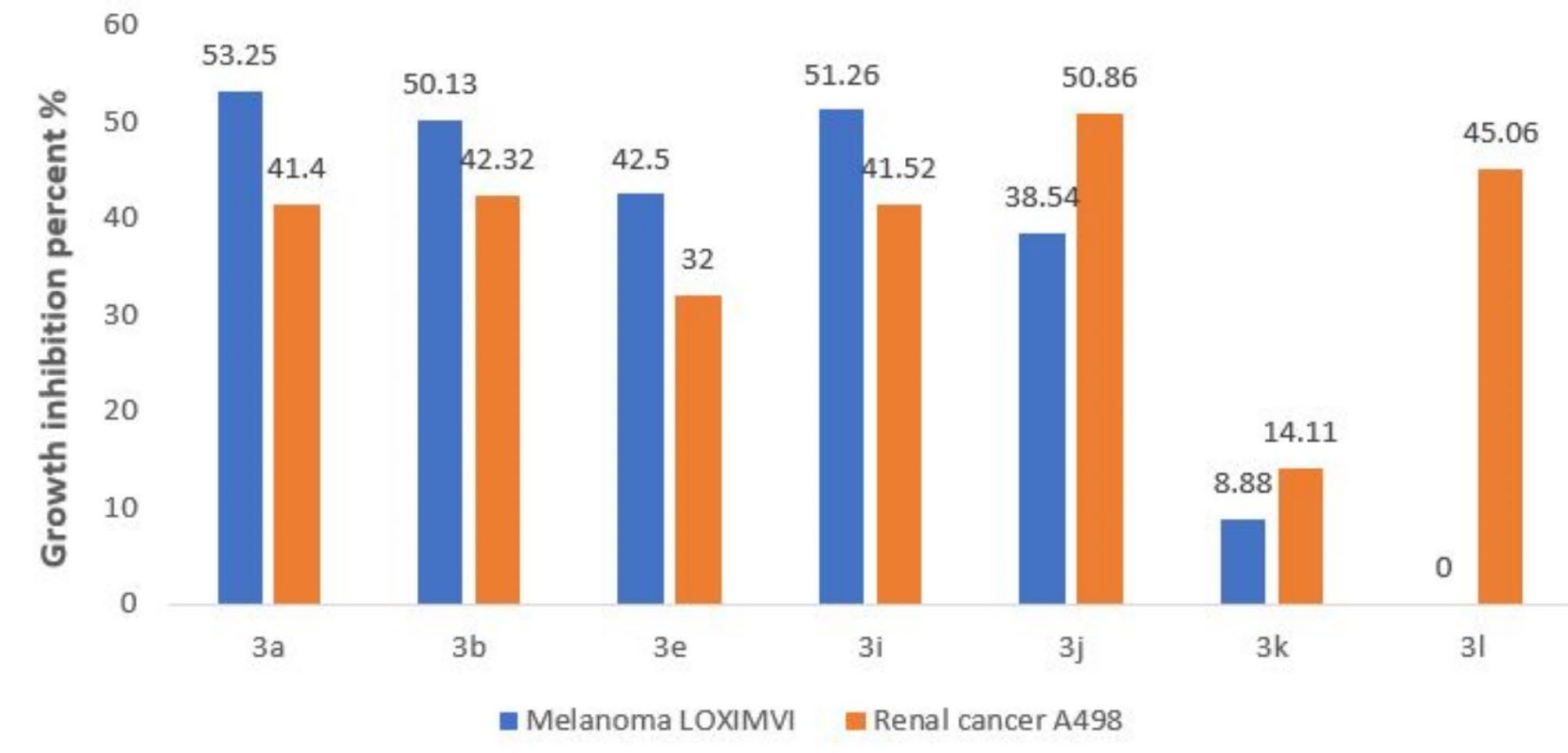
The growth inhibition percent induced by compounds **3a**,** 3b**,** 3e**,** 3i**,** 3j**,** 3k**, and **3 L** against renal cancer A498 and melanoma LOX IMVI cell lines.

Following the guidelines established by the drug evaluation department of the National Cancer Institute (NCI), USA, all target compounds **3a–l** were examined against 60 cancer cell lines at a single concentration of 10 µM. Results listed in Table 1 demonstrated a week to moderate activity for most tested compounds with high activity observed with melanoma LOX IMVI and renal A498 cell lines. In detail, the un-substituted **3a** exhibited moderate activity against renal UO-31, melanoma LOX IMVI, and breast MCF7 cancer cell lines with growth inhibition percentages of 53.25, 41.40, and 55.93, respectively (Fig. 3**)**. Moreover, the naphthyl analogue **3j** exhibited promising activity against melanoma LOX IMVI, melanoma SK-MEL-5, renal cancer cells A498 and UO-31, breast cancer cells MCF7 with growth inhibition precents of 38.54, 46.13, 50.86, 48.10, and 41.71, respectively. Both the 4-fluoro **3i** and 2-methyl derivatives **3b** exhibited reasonable activity towards melanoma LOX IMVI cell line with growth inhibition percentages of 51.26 and 50.13 and against renal cancer A498 with 41.52 and 41.32, respectively. Meanwhile, the 4-methoxy derivative **3e** showed good activity against Melanoma cells LOXIMVI with growth inhibition percentage of 42.50. In addition, the 4-nitro analogue **3k** exhibited growth inhibition percentages of 41.99 and 42.46 against renal cancer cells UO-31 and CNS cancer cells SNB-75 while the un-substituted thiazolidine 2,4-dione hybrid **3 L** showed moderate activity against renal cancer cells A498 and UO-31, CNS cancer cells SNB-75 with growth inhibition percentages of 45.06, 41.97, and 51.13, respectively (Table 1).

**Table 1:** Growth inhibition percentage (GI %) induced by single dose (10^−5^ M) of compounds **3a-l** against 9 cancer panels of 60 different cancer cell lines according NCI protocol.

Cell line	Growth Inhibition (%)											
	3a	3b	3c	3d	3e	3f	3 g	3 h	3i	3j	3k	3L
Leukemia												
CCRF-CEM	9.8	7.4	2.9	-	-	-	-	-	4.2	-	NT	NT
HL-60(TB)	-	20.8	6.2	-	-	1.9	-	-	-	-	-	-
K-562	22.5	14	-	-	-	1.1	-	0.7	-	10.9	-	-
MOLT-4	12.6	24	-	-	-	7.2	-	-	-	17.5	-	-
RPMI-8226	11.4	23.7	NT	-	-	-	-	-	-	6.1	8.7	6.0
SR	29.9	8.2	-	-	4.3	19.5	5.4	10.7	3.6	14.1	9.7	-
Non-Small Cell Lung Cancer												
A549/ATCC	14.2	5.5	-	7.8	7.9	1.3	3.5	3.4	10.4	9.3	5.5	6.7
EKVX	-	3.4	2.4	2.3	0.4	10.6	-	2.1	-	9.1	24.4	23.8
HOP-62	-	0.9	-	5.2	-	-	-	2.9	2.9	15.1	19.7	16.8
HOP-92	-	NT	-	-	8.3	0.8	-	9.7	5.7	13.3	0.2	0.7
NCI-H23	10.2	12	1.8	7.9	2.6	-	2.6	5.3	6.5	13.1	6.2	5.7
NCI-H322M	9.4	12.7	-	6.8	6.0	4.2	5.1	11.8	11.5	26.6	-	0
NCI-H460	3.7	12.9	-	-	0	-	-	0	-	3.9	-	0
NCI-H522	27.4	14.0	1.7	20.7	25.7	7.8	3.3	18.3	22.9	18.5	NT	NT
Colon Cancer												
HCT-116	19.3	22.5	-	-	0	-	-	1.8	4.5	10.6	-	-
CNS Cancer												
SF-268	12.4	22.1	3.7	2.4	2.3	-	4.4	2.3	3.1	29.1	13.3	15.1
SF-539	8.9	10.6	0.9	9.5	6.5	4.4	0.5	1.2	5.8	9.9	6.0	3.3
SNB-19	9.8	13.5	-	1.0	3.8	0.9	-	0	6.2	20.1	-	0
SNB-75	29.1	-	NT	18.2	12.6	4.9	5.9	15.5	16.5	28.4	**42.5**	**51.1**
Melanoma												
LOX IMVI	**53.3**	**50.1**	3.5	26.5	**42.5**	2.4	1.8	38.2	**51.3**	**38.5**	8.9	-
MALME-3 M	30.4	9.1	-	9.8	2.9	5.9	-	10.9	14.8	13.1	-	-
M14	8.2	9	-	0.8	-	-	0.5	0.7	4.6	16.7	-	-
MDA-MB-435	7.8	8.0	-	-	-	-	-	-	1.6	12.8	-	-
SK-MEL-2	10.1	4.0	-	2.9	6.5	2.8	-	7.3	-	-	-	-
SK-MEL-28	9.0	2.5	-	-	-	-	-	-	1.5	4.5	-	**49.5**
SK-MEL-5	24.6	9.0	3.8	2.3	3.9	1.9	-	1.1	5.5	**46.1**	0.8	-
UACC-257	21.1	1.0	-	8.1	19.7	12.3	6.7	5.1	12.3	19.1	-	-
UACC-62	28.6	22.8	4.2	10.3	7.0	1.4	3.7	6.2	19.3	20.4	2.0	8.4
Ovarian Cancer												
IGROV1	8.5	22.0	3.2	1.6	-	-	-	-	1.3	25.9	15.9	15.3
OVCAR-4	2.5	12.7	-	2.6	-	-	-	5.1	0.5	16.4	13.7	19.2
OVCAR-8	15.4	2.6	-	3.1	5.3	2.9	1.9	5.3	6.8	9.0	-	-
Renal Cancer												
786-0	21.2	8.9	0.9	8.5	18.7	2.4	-	11.3	18.2	18.5	14.1	4.8
A498	**41.4**	**42.3**	5.3	8.9	32.0	15.5	2.3	7.7	**41.5**	**50.9**	29.9	**45.1**
ACHN	14.5	2.3	2.3	4.2	1.2	-	-	0.7	4.6	11.5	0.1	0.6
CAKI-1	10.9	20.5	1.5	5.0	5.5	1.7	2.2	4.4	4.9	18.0	19.5	22.7
RXF 393	-	-	7.2	-	-	-	-	-	-	11.2	47.9	30.7
SN12C	2.6	7.5	-	3.7	-	0.8	0.6	0.4	2.2	24.1	8.7	14.6
TK-10	-	-	-	-	11.3	8.7	-	1.1	4.7	-	33.5	28.7
UO-31	36.0	26.2	16.5	20.7	18.5	14.5	1.5	28.0	21.7	**48.1**	41.1	41.0
Breast Cancer												
MCF7	**55.9**	11.7	-	1.9	-	3.9	3.6	-	1.3	**41.7**	-	-
MDA-MB231	25.2	21.4	-	8.0	12.4	0.1	-	13.7	26.4	25.2	11.2	10.1
HS 578T	8.8	17.2	-	8.2	2.4	1.2	3.2	-	3.0	30.2	NT	NT
BT-549	7.3	8.5	NT	3.0	10.3	3.4	-	3.8	8.6	20.5	1.0	4.8
T-47D	8.1	30.5	-	-	0.6	0.9	-	2.0	5.0	7.5	-	-
MDA-MB-468	12.9	6.6	-	0.5	-	-	-	-	7.6	4.7	-	2.1
Prostate Cancer												
PC-3 71.03	-	14.5	1.1	-	-	-	-	-	-	-	-	2.4
DU-145	-	6.6	-	-	-	-	-	-	-	21.9	7.0	8.3

NT: not tested, - means no detected activity, **bold** numbers indicate GI > 40%.

##### In vitroanti-cancer activity of compounds 3a-b, 3i-l against melanoma LOX IMVI and renal A498 cell lines

Based on the above-mentioned results in Table 1, compounds with the highest observed activity namely **3a**,** 3b**,** 3i**,** 3j**, and **3 L** were selected for IC_50_ determination against the most sensitive cell lines; melanoma LOX IMVI and renal cancer A498 cell lines in comparison to doxorubicin and Camptothecin as reference compounds using MTT assay. Results showed moderate anticancer activity of compounds **3a**, **3b** and **3i** against melanoma LOX IMVI with IC_50_ values of 26.70 ± 1.50, 42.70 ± 2.41, and 25.40 ± 1.43 µM in comparison to doxorubicin and cisplatin with IC_50_ values of 7.03 ± 0.40 and 5.07 ± 0.29 µM, respectively. Concerning A498 renal cancer cell line, compound **3j** showed promising anticancer activity with an IC_50_ value of 33.90 ± 1.91 µM but lower than the reference compounds doxorubicin and cisplatin which exhibited IC_50_ values of 3.59 ± 0.2 and 7.92 ± 0.45 µM, respectively (Table 2).

**Table 2:** In-vitro cytotoxicity expressed as half inhibitory concentration (IC_50,_ µM ± SEM) of compounds **3a-b** and **3i-3 L** against human melanoma LOX IMVI and A498 renal cancer cell lines and normal cell line WI 38 in comparison to the conventional cisplatin and doxorubicin.

Compound	In vitro cytotoxicity (IC_50_)		
	LOX IMVI (µM ± SEM)	A498 cell line (µM ± SEM)	W138 normal cell line (µM ± SEM)
**3a**	26.7 ± 1.5	> 150	-
**3b**	42.7 ± 2.41	> 150	-
**3i**	25.4 ± 1.43	147 ± 8.3	45.13 ± 2.43
**3j**	110 ± 6.23	33.9 ± 1.91	-
**3k**	> 150	> 150	-
**3 L**	109 ± 6.15	80.7 ± 4.55	-
**Cisplatin**	5.07 ± 0.29	7.92 ± 0.45	-
**Doxorubicin**	7.03 ± 0.4	3.59 ± 0.2	18.13 ± 0.98

##### In vitro cytotoxicity activity of compounds**3i** against normal cell line W138

The most potent compound as an anticancer agent **3i** in comparison to doxorubicin was tested for activity against the normal cell line WI 38 to confirm their selective activity towards cancer cellsResults showed that the newly synthesized active compound **3i** has a good margin of safety with an IC_50_ equal 45.13 ± 2.43 µM against the normal cell line WI 38 almost double that against LOX IMVI cells and with more selectivity to that of the cytotoxic doxorubicin which exhibited IC_50_ of 18.13 ± 0.98 µM as shown in Table 2.

##### Topoisomerases inhibition assay

**Fig. 4:**
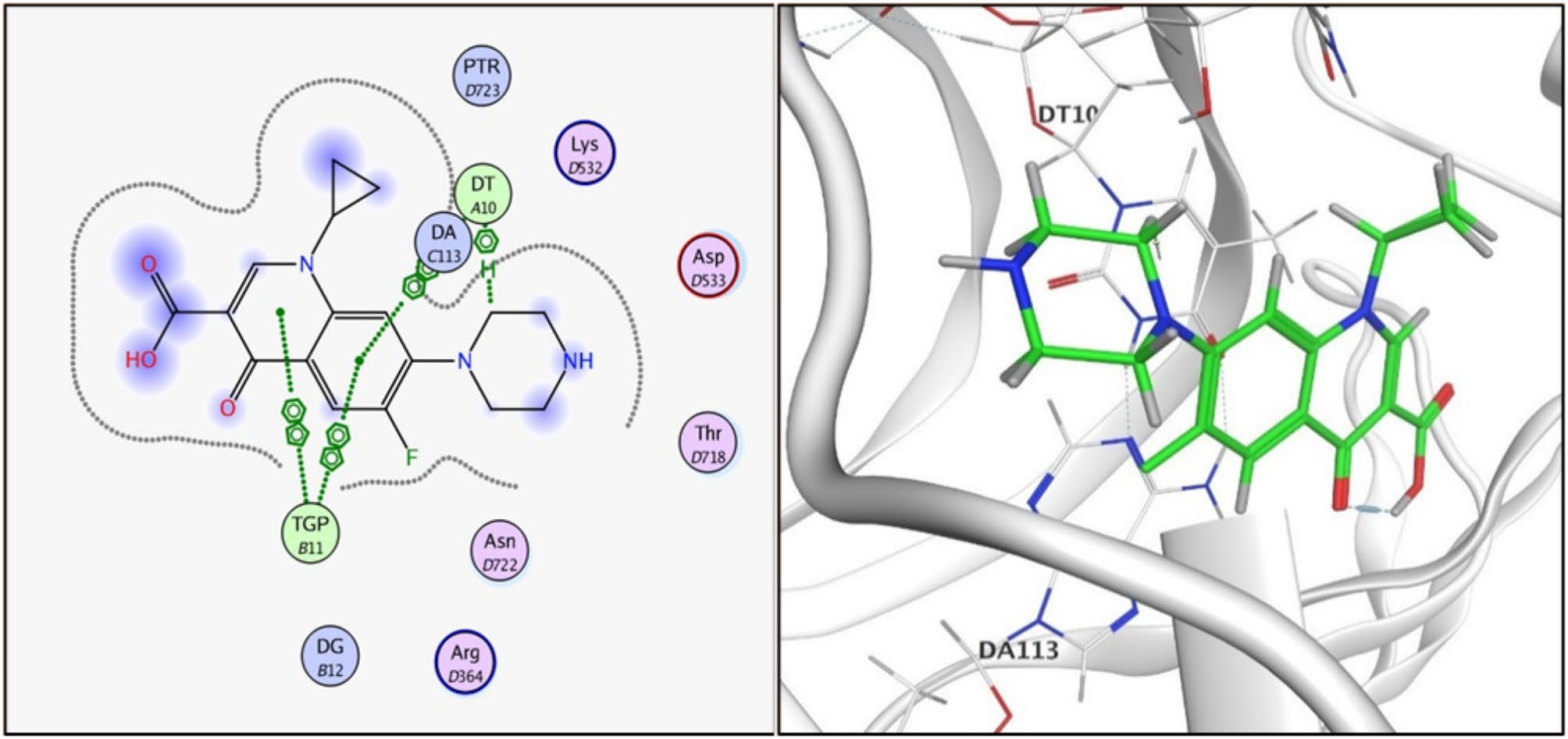
2D and 3D illustration of **ciprofloxacin** docked into the active site of topoisomerase I enzyme (PDB: 1T8I).

**Fig. 5:**
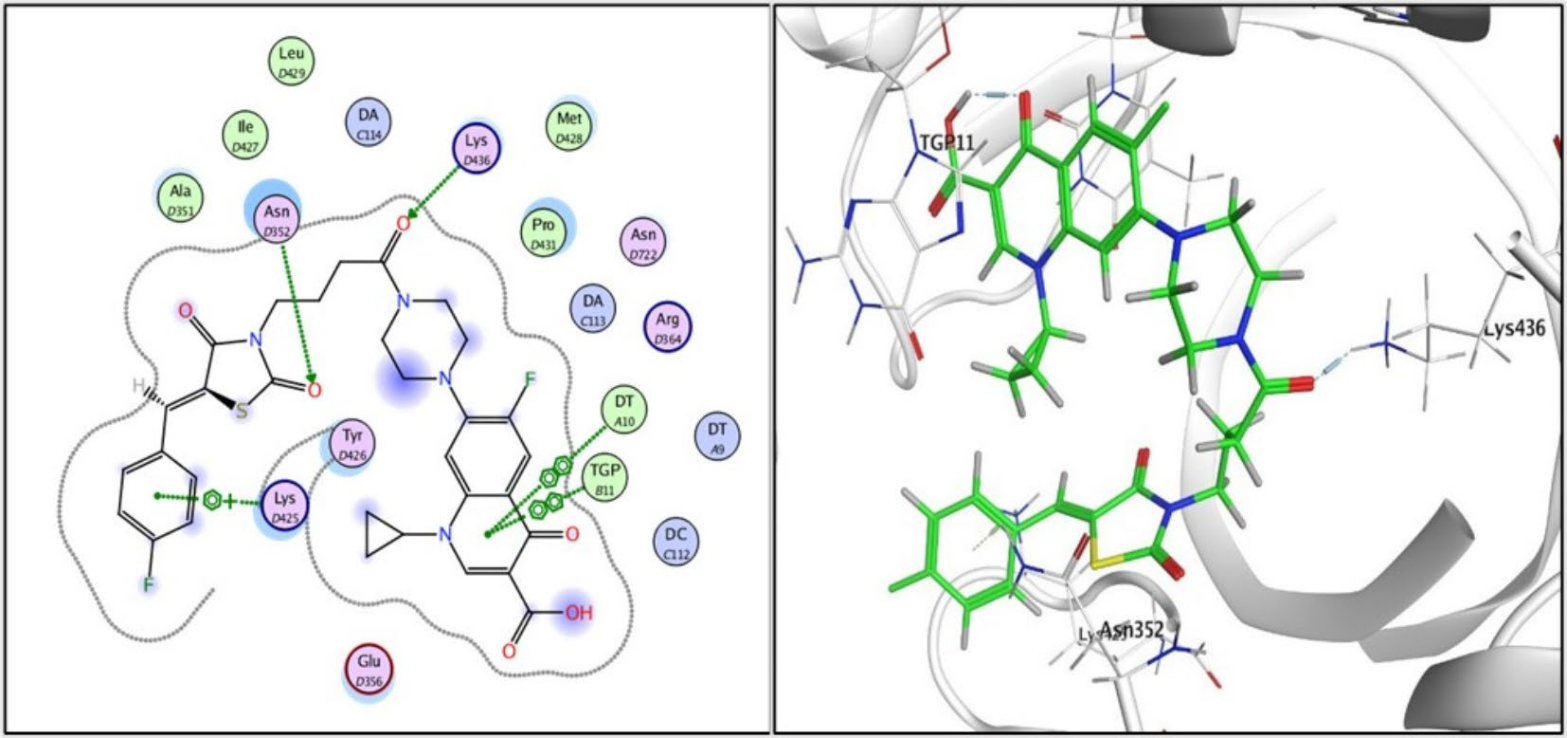
2D and 3D illustration of **3i** docked into the active site of topoisomerase I enzyme (PDB: 1T8I).

Compounds with highest cytotoxicity observed; **3a-b** and **3i-l;** were screened for topoisomerase I and II inhibition in comparison to cisplatin and doxorubicin. Results of topoisomerase I inhibition revealed that compounds **3b** and **3i** (IC_50_ = 7.2 and 4.8 **µ**M) were of comparable potency to that of doxorubicin (IC_50_ = 6.5 **µ**M) and about three folds more potent than that of cisplatin (IC_50_ = 11.3 **µ**M). Furthermore, compound **3a** was less potent than cisplatin, both of IC_50_ around 14 **µ**M, while compounds **3j-l** were of lower activity with IC_50_ values of 23.50, 36.6 and 18.80 µM. Nevertheless, all compounds were less potent in Topoisomerase II inhibition. Compounds **3a-b** and **3i-l** displayed topoisomerase II inhibition showing IC_50_ values of 35.60, 31.10, 15.00, 39.80, 18.7 and 14.30 µM, respectively. Compound **3i** showed topoisomerases I/II inhibition activity comparable to that of cisplatin (Table 3; Figs. 4 and 5).

**Table 3:** Topoisomerase I/II inhibitory activity by compounds **3a- b**,** 3i-l**, doxorubicin and camptothecin measured as IC_50_ in µM ± SEM.

Compound	Topoisomerase I IC_50_ µM ± SEM	Topoisomerase II IC_50_ µM ± SEM
**3a**	14.50 ± 0.79	35.60 ± 1.94
**3b**	7.15 ± 0.39	31.10 ± 1.69
**3i**	**4.77 ± 0.26**	**15.00 ± 0.81**
**3j**	23.50 ± 1.28	39.80 ± 2.17
**3k**	36.60 ± 1.99	18.70 ± 1.02
**3 L**	18.80 ± 1.02	14.30 ± 0.78
**Cisplatin**	5.71 ± 0.31	11.30 ± 0.62
**Doxorubicin**	3.36 ± 0.18	6.49 ± 0.35

Significant values are in bold.

##### Docking study

**Fig. 6:**
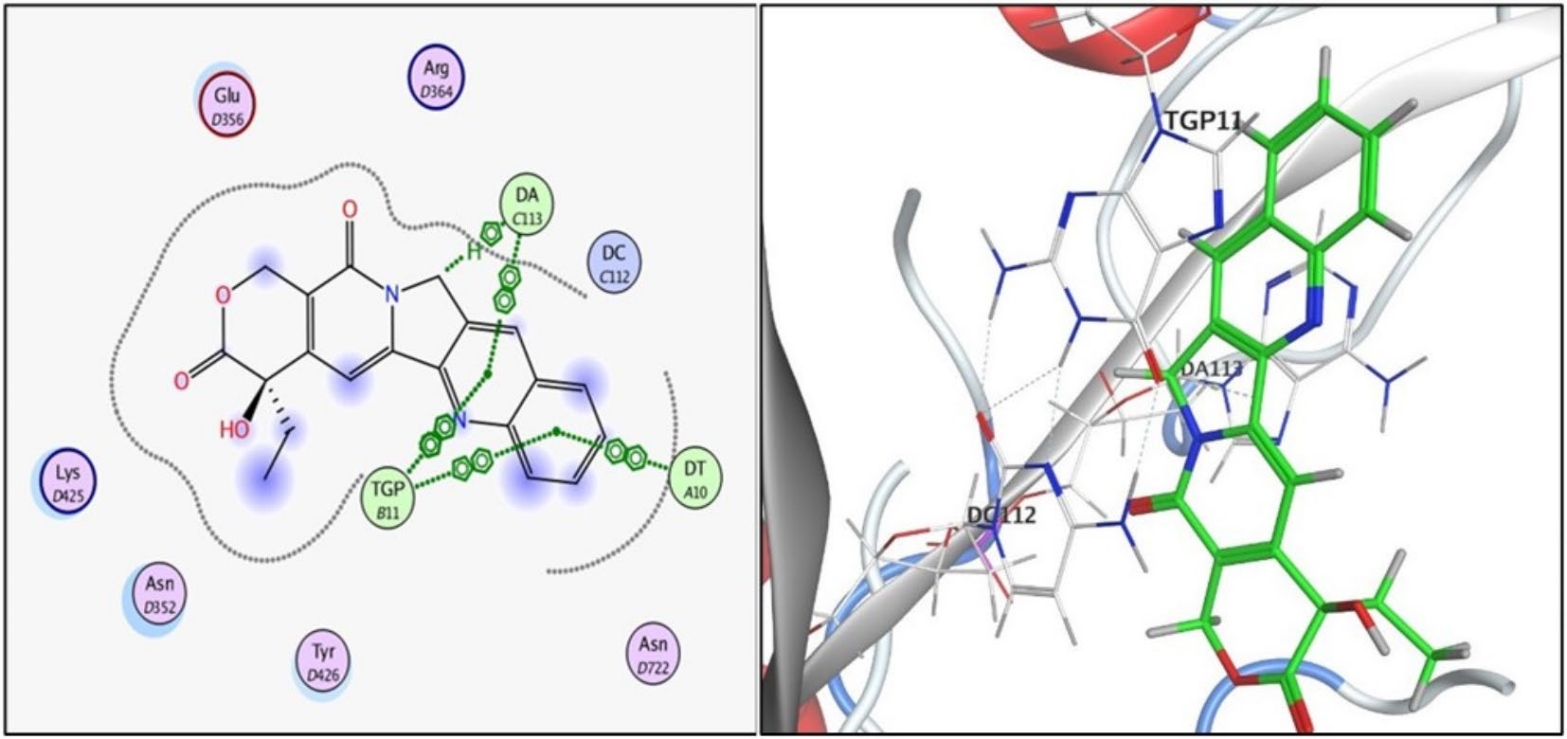
2D and 3D illustration of **Camptothecin** docked into the active site of topoisomerase I enzyme (PDB: IT8I).

Compound **3i**, the one with highest activity against both topoisomerases I/II, and ciprofloxacin were theoretically studied via docking into the active pocket of topoisomerase I enzyme (PDB: 1T8I^36^ and topoisomerase II (PDB: 3QX3)^36^ to spot the potential binding interactions induced by the new structural alterations to ciprofloxacin compared to the parent ciprofloxacin using camptothecin as appositive control for Topo I and doxorubicin as appositive control for Topo II. MOE 2014 software was used to perform docking stimulations. The ligands found in both PDB files were redocked into the active site to validate the docking methodology, poses obtained were almost identical to original binding patterns (Supplementary data file). Redocking of the co-crystalized ligands were done to confirm the validation of the docking protocol employed. The obtained poses were almost identical to the co-crystalized ligand separated, supporting information. Additionally, The validity of the docking method employed and its ability to accurately predict the right orientation of ligands is supported by the value of the root-mean-square deviation (RMSD) of the docked poses for the potent derivative **3i**, parent ciprofloxacin, and etoposide or camptothecin with the co-crystallized ligand relative to crystal orientation being less than 1.5 Å. Negative binding scores ensure that fluoroquinolone derivatives spontaneously bind to the active site of the topoisomerases I/II enzymes. The docked compound **3i** compound has high affinity for the topoisomerase I and II enzymes with binding free energy (∆G) values of -10.01 and − 7.49, comparable to camptothecin affinity to Topo I, doxorubicin and etoposide affinity to Topo II with binding free energy (∆G) values of -7.28 and − 7.64, and 7.76as shown in Tables 4 and 5. Compound **3i** showed good binding with the active site of topoisomerase I (PDB: 1T8I) *via* hydrogen bonding with amino acids residue ASN 352, LYS 436 in addition to binding with nucleotide base DA 10 and Van der walls interaction, Fig. 5. Compound **3i** also showed binding with the active site of topoisomerase II (PDB: 3QX3) by Van der walls interaction and hydrogen bonding with thiazolidine-2,4-dione moiety and quinolone nucleus, Fig. 6. Importantly, the newly synthesized compound **3i** showed extra-hydrophobic interactions *via* thiazolidine-2,4-dione moiety benzylidene moiety and butyryl linker as shown in Figs. 5 and 6.

**Table 4:** Binding interactions and energy scores (kcal/mol) of compounds **3i**, ciprofloxacin and camptothecin docked into the pocket of the active site of topoisomerase I (PDB: 1T8I).

Compound	Energy score (kcal/mol)	Ligand interaction		
		Amino acid residue	Interaction type	Length (Aº)
**Ciprofloxacin**	-7.128	DT 10 TGP 11 DT 10 TGP 11	H-pi Pi-Pi Pi-Pi Pi-Pi	4.50 3.95 3.99 3.38
**3i**	-10.013	LYS 436 ASN-352 LYS-425 DT-10 TGP-11	H-bond H-bond Pi-cation Pi-Pi Pi-Pi	2.73 3.46 3.96 3.97 3.42
**Camptothecin**	-7.276	DA 113 DA 113 TGP 11 TGP 11 DT 10 TGP 11	H-Pi Pi-Pi Pi-Pi Pi-Pi Pi-Pi Pi-Pi	3.34 3.63 3.58 3.53 3.75 3.45

**Table 5:** Binding interactions and energy scores (kcal/mol) and of compounds **3i**, **ciprofloxacin**,** etoposide**, and **doxorubicin** docked into the active site of topoisomerase II (PDB: 3QX3).

Compound	Energy score (kcal/mol)	Ligand interaction		
		Amino acid residue	Interaction type	Length (Aº)
**Ciprofloxacin**	-6.735	MG 2000	Metal interaction	2.06
**3i**	-7.491	LYS 739 Ile 372 DT 9	H-bond H-bond Pi-Pi	2.78 3.20 3.42
**Doxorubicin**	-7.639	DC 14 DT 15 MG GLU 477	H-bond pi-H Meal interaction H-bond	3.28 2.92 3.19 3.87
**Etoposide**	-7.758	DG 13 DG 13	H-bond H-bond	2.93 2.93

**Fig. 7:**
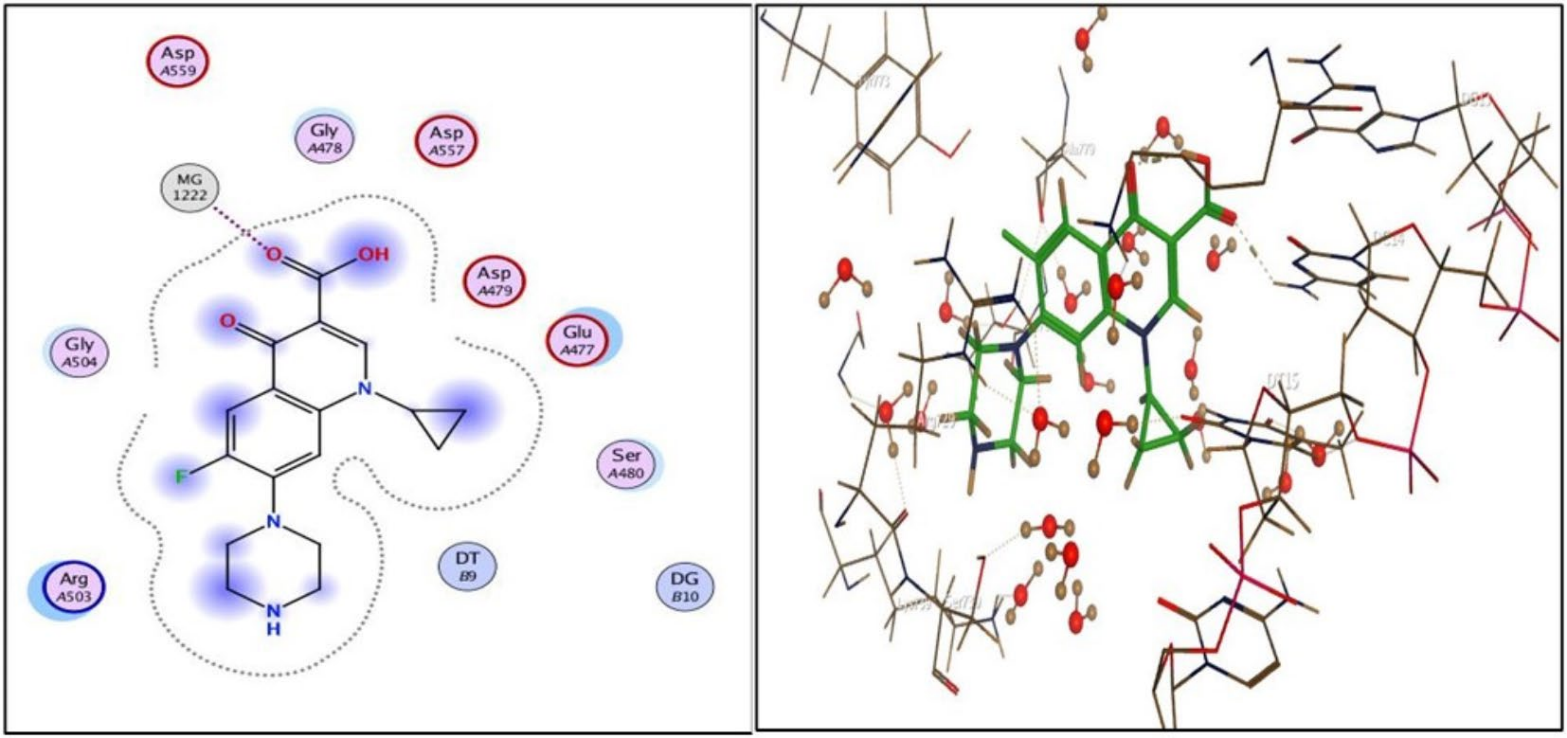
2D and 3D illustration of **ciprofloxacin** docked into the active site of topoisomerase II enzyme (PDB: 3QX3).

**Fig. 8:**
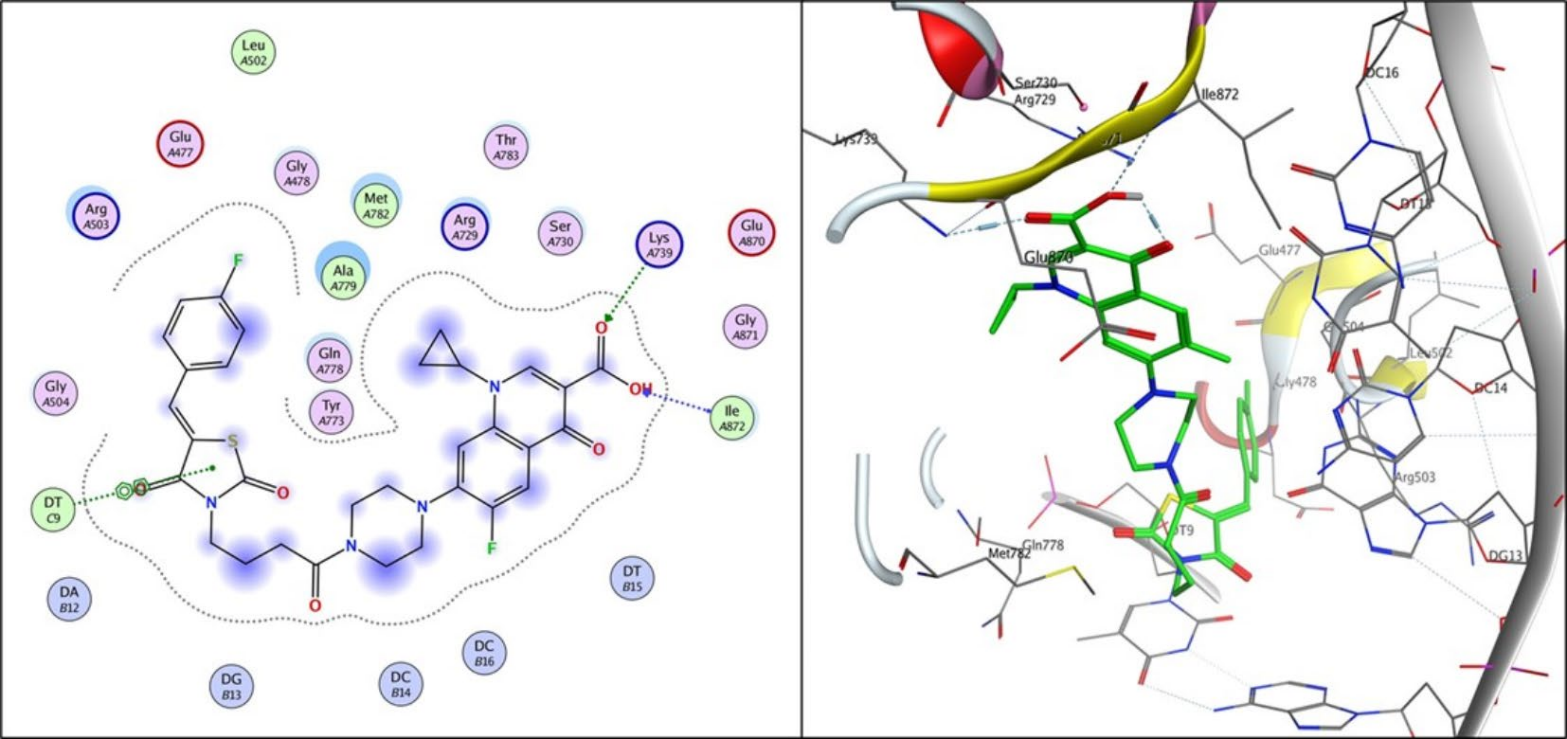
2D and 3D diagram of **compound 3i** docked into the active site of topoisomerase II enzyme (PDB: 3QX3).

**Fig. 9:**
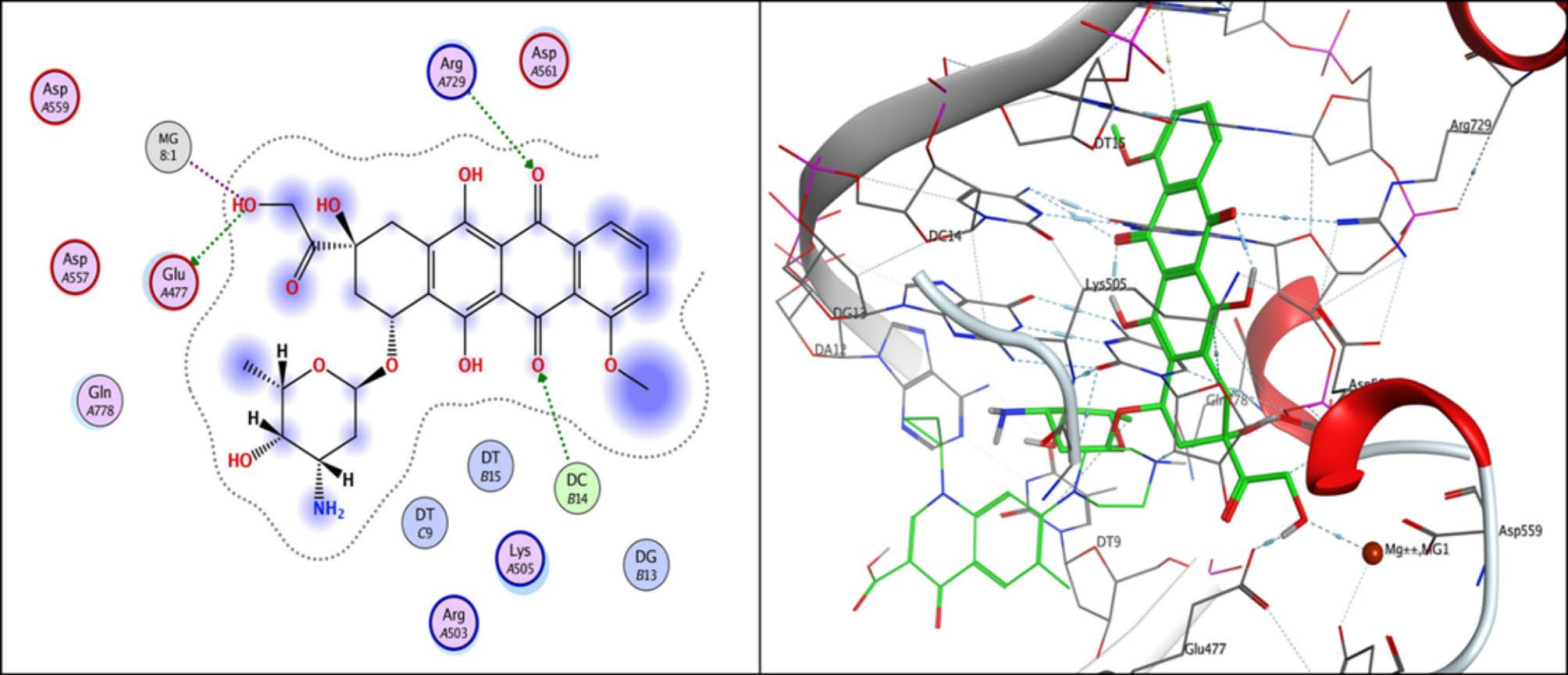
2D and 3D illustration of **doxorubicin** docked into the active site of topoisomerase II enzyme (PDB: 3QX3).

Docking records demonstrated that the new compound **3i** and ciprofloxacin would possibly fit into the topoisomerases I/II pocket in the same manner as that of camptothecin and doxorubicin (Figs. 4, 5, 6 and 7, 8, 9). Compound **3i** potentially can form H bonds with both Lys 739 and Ile 872 in the active site of Topo II, while 2 H bonds formed with Lys 436 and Asn 352 and a pi-cation bond with Lys 425 seen in the active site of Topo I.

Compared to ciprofloxacin, compound **3i** can for additional Pi-Pi interactions, and hydrogen bonding which might explains the difference in energy score between compound **3i** and its parent ciprofloxacin (Figs. 4, 5 and 7, 8 and ; Tables 4 and 5). Generally, the docking data supported the reference to Topoisomerase as a potential mechanism for the action of compound **3i**.

##### Cell cycle analysis and apoptosis assay

**Fig. 10:**
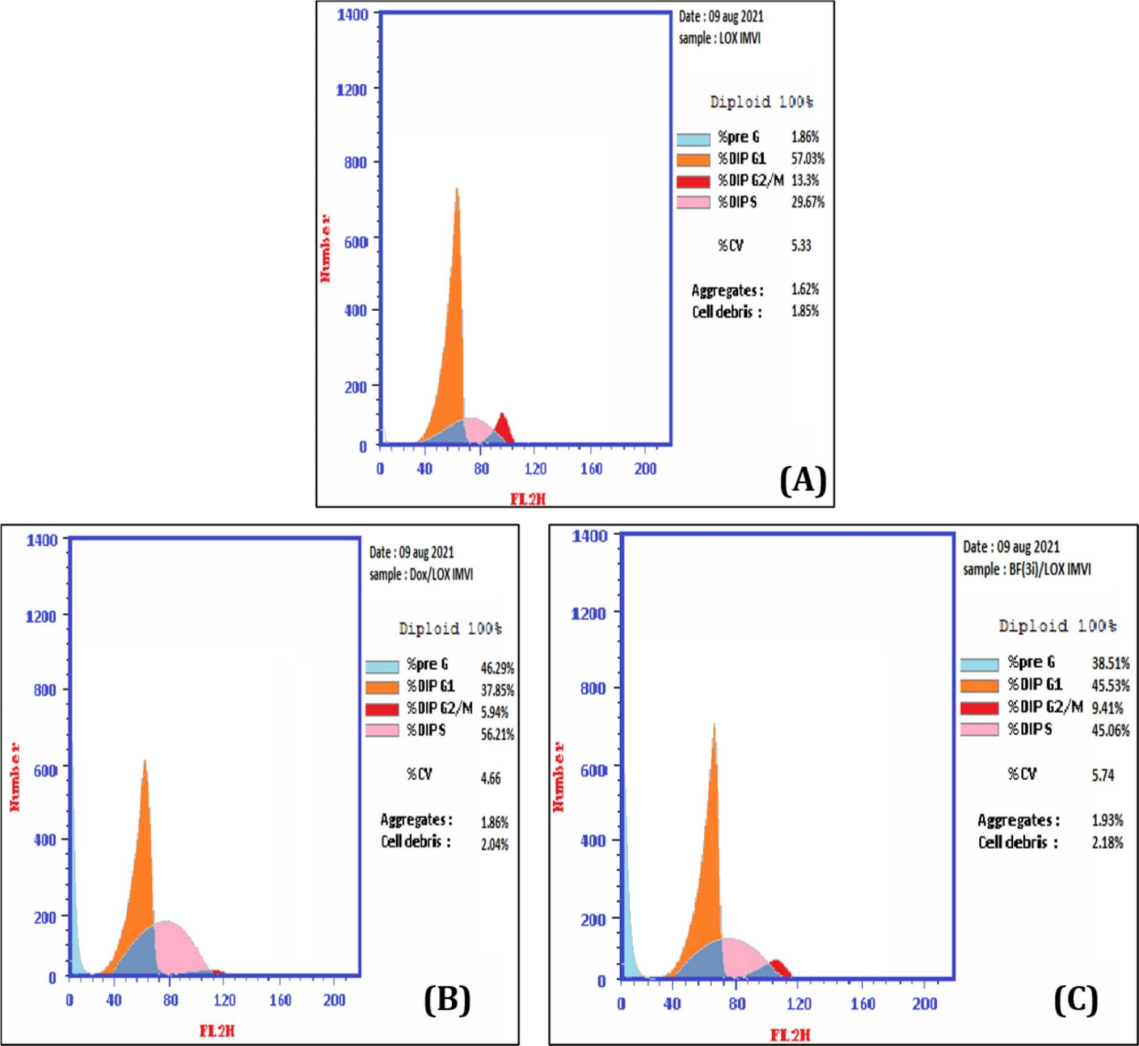
Flow cytometric analysis of the cell cycle distribution of melanoma LOX IMVI cells treated with DMSO (Control, **A)**, doxorubicin (IC_50_, 7.03 µM, **B**) and compound **3i** (IC_50_, 25.4 µM, **C**) fixed and stained by propidium iodide.

Herein, the effect of compound **3i** on cell cycle progress in melanoma LOX IMVI cell line was analyzed in comparison to doxorubicin using flow cytometry assay. Results showed that compound **3i** increased the percentage of cells at pre-G1 by 20 folds indicating its apoptotic potential that encourage cancer cell death. Also, percentage of cells in S phase was raised from 29.67 to 45.06 when treating melanoma LOX IMVI cells via compound **3i** at its previously measured IC_50_ suggesting the tendency of compound **3i** to induced cycle arrest at S phase (Table 6; Fig. 10).

**Table 6:** DNA content (%) at the four different stages of the cell cycle of melanoma LOX IMVI cancer cell line treated with DMSO as control, doxorubicin, and compound **3i** measured after cytometric analysis of the cell cycle distribution.

Compound	%G0-G1	%S	%G2/M	%Pre-G1
	DNA content			
**3i**/LOX IMVI	45.53	45.06	9.41	38.51
Doxorubicin /LOX IMVI	37.85	56.21	5.94	46.29
DMSO/ LOX IMVI	57.03	29.67	13.3	1.86

**Fig. 11:**
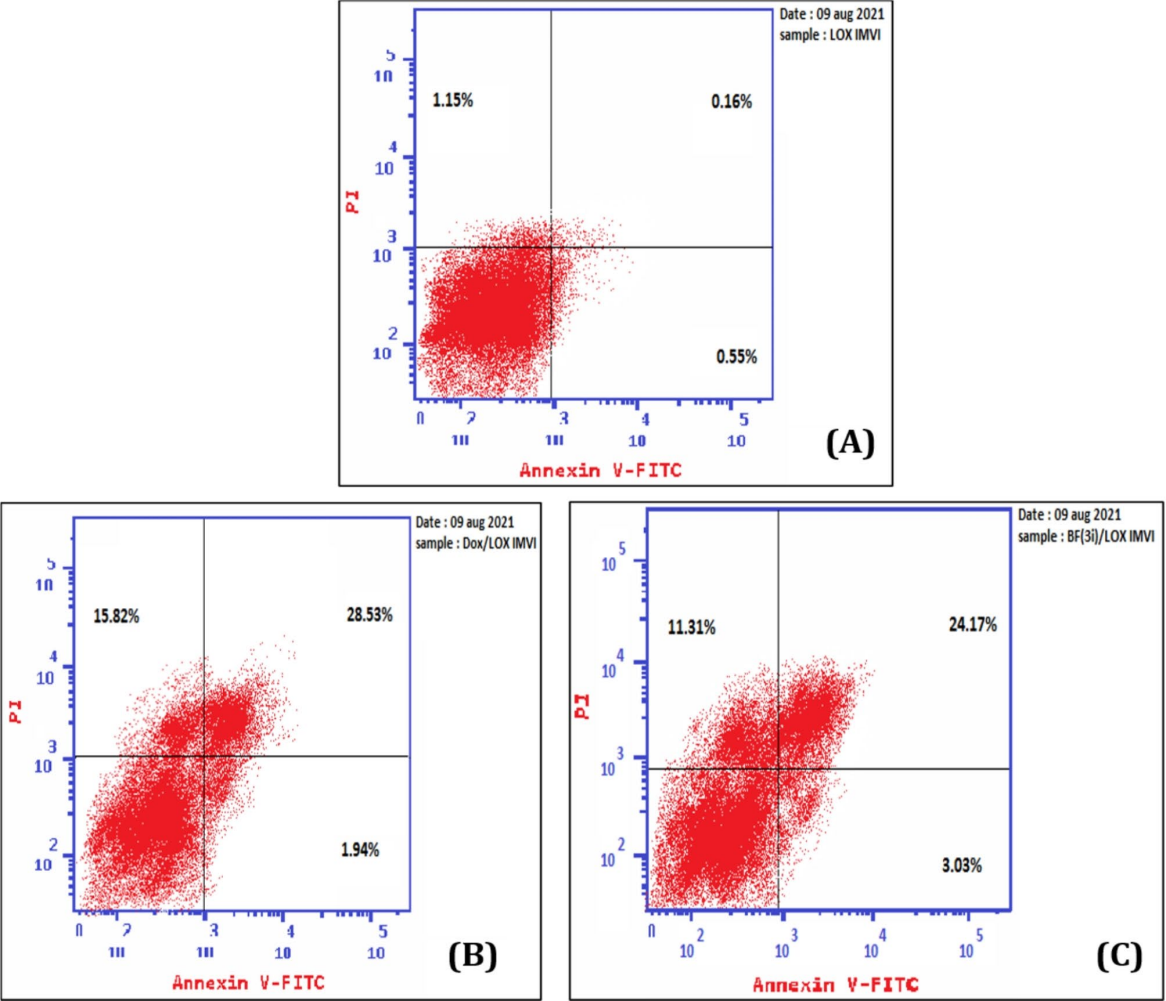
The apoptosis and necrosis of melanoma LOX IMVI; measured by Annexin V-FITC/PI assay; induced by DMSO (control, **A**), doxorubicin (IC_50_, 7.03 $\:\mu\:$M, **B)** and **3i** (IC_50_, 25.4 µM, **C**). The four quadrants are identified as: lower left quadrant; LL: viable cells, lower right quadrant; LR: early apoptotic cells, upper right quadrant; UR: late apoptotic cells, upper left quadrant; UL: necrotic cells.

The apoptotic ability of compound **3i** were studied to decide whether its anticancer activity against melanoma LOX IMVI cell line is associated with an increase of apoptosis and necrosis or not. Analysis using annexin assay expressed that when melanoma LOX IMVI cell line treated with IC_50_ concentration of compound **3i**, significant levels of early and late apoptosis in addition to necrosis were induced (3.03, 24.17 and 11.31%, respectively) which are comparable to those induced by the conventional cytotoxic drug doxorubicin 1.94, 28.53 and 15.82, respectively (Tables 7and Fig. 11).

**Table 7:** The apoptosis and necrosis assay of melanoma LOX IMVI cell treated with IC_50_ concentration of compound **3i**, doxorubicin against negative control.

Compound	Apoptosis			Necrosis
	Total	Early	Late	
3i/ LOX IMVI	38.51	3.03	24.17	11.31
Dox. / LOX IMVI	46.29	1.94	28.53	15.82
cont. LOX IMVI	1.86	0.55	0.16	1.15

##### Effect of compound 3i on the protein expression level of Caspase-3, Bax and PARP-1

**Fig. 12:**
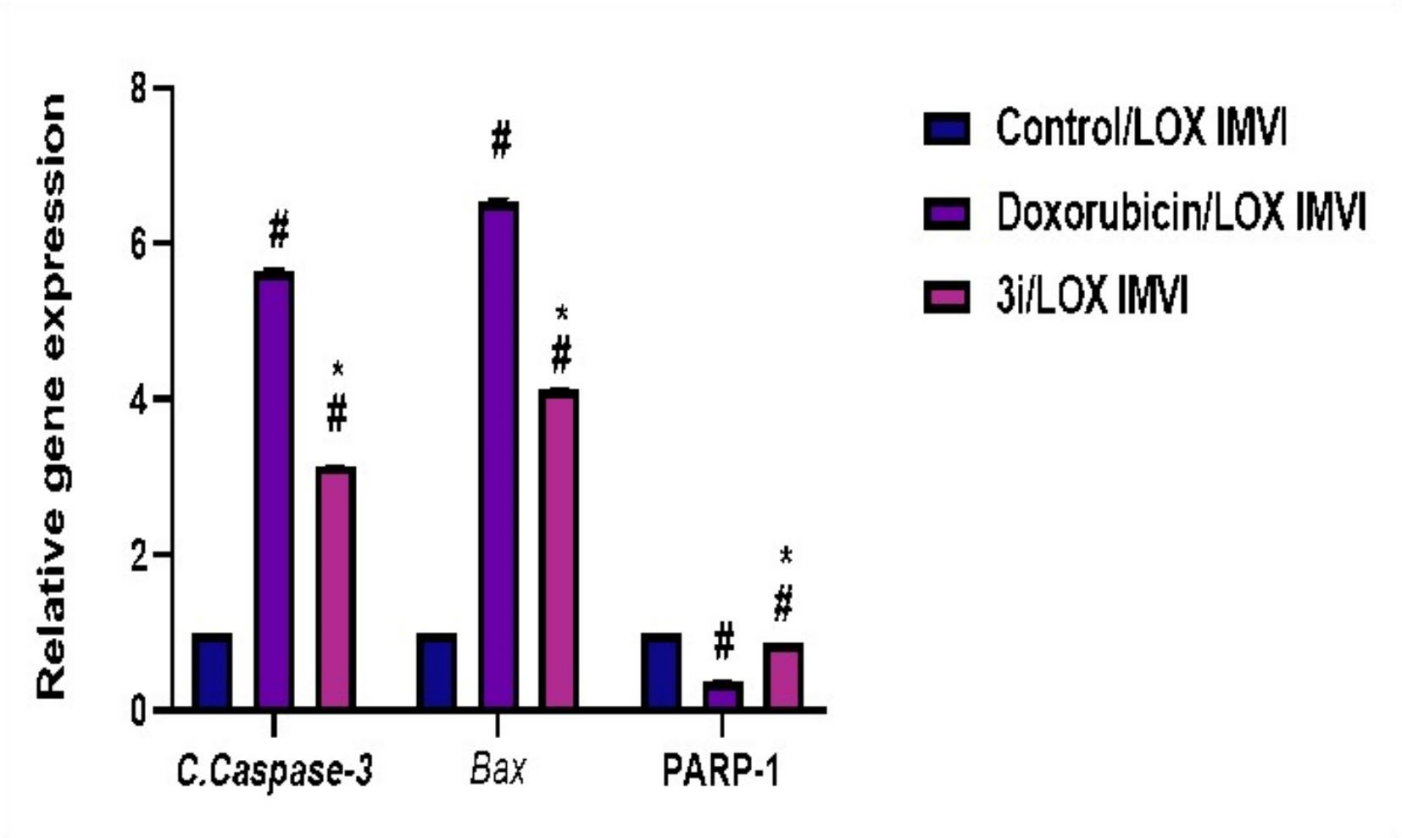
Effect of 3i/LOX IMVI on the expression of C.Caspase-3, Bax, and PARP-1 proteins, One-way ANOVA (analysis of variance) followed by the Bonferroni post hoc test for multiple comparisons, and Two-way ANOVA tests are performed, with #*P* < 0.05 used to compare to Control/LOX IMVI group, * *P* < 0.05 used to compare the Doxorubicin/LOX IMVI group.

**Fig. 13:**
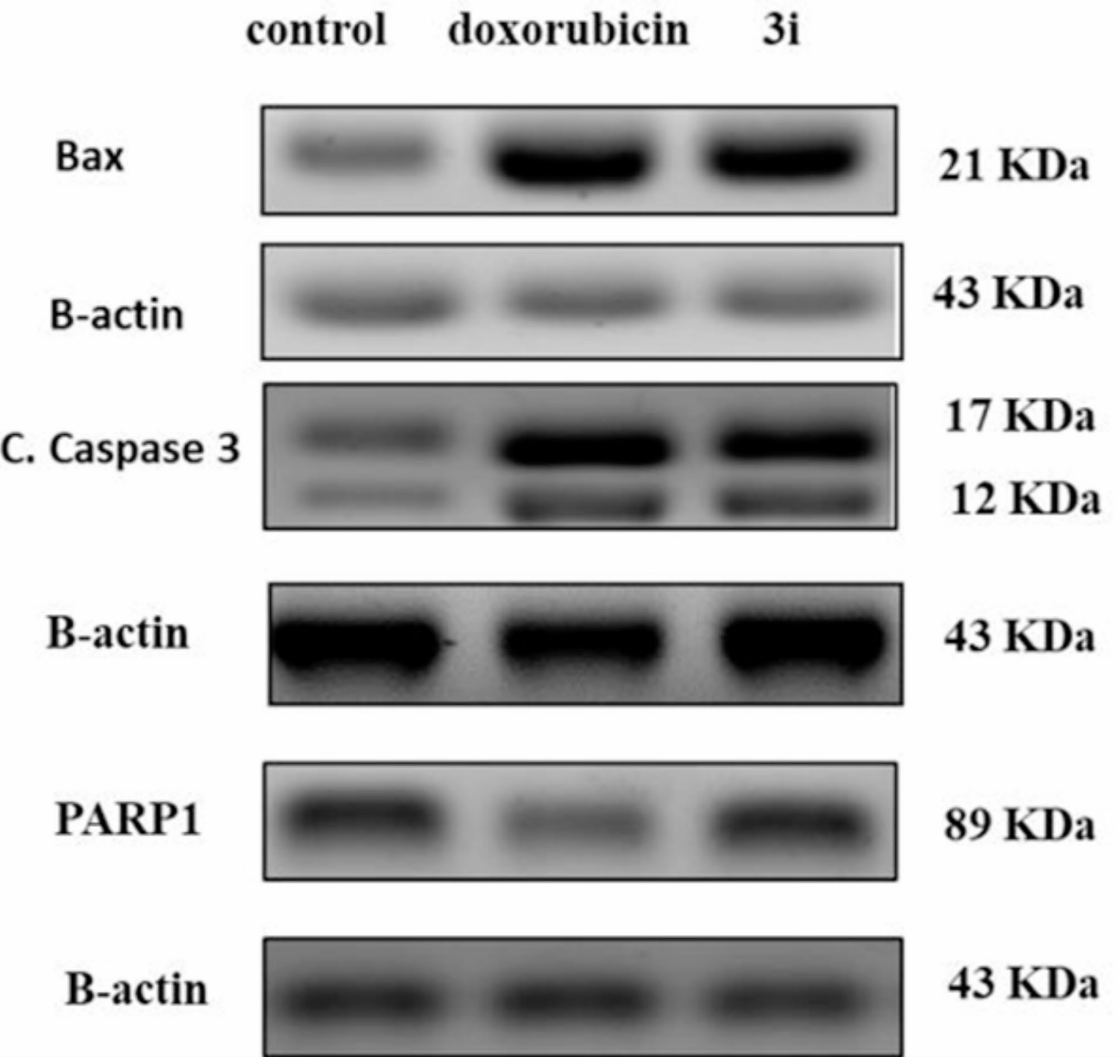
Western blotting analysis of the effect of compound **3i** on Bax, caspase 3 and PARP-1.

Western plotting was used to evaluate the effect of compound **3i** on the expression level of active caspase-3, Bax and PARP-1. Results implied that in comparison to the untreated control LOX IMVI cells, the expression level of caspase-3 was 49 folds higher in cells treated with IC_50_ concentration of compound **3i**. Also, compound **3i** showed comparable expression levels of caspase-3 to that of doxorubicin (Table 8 and Figs. 12 and 13). In addition, compound **3i** increased the expression level of the apoptotic protein Bax in LOX IMVI cell line by approximately 3 folds over its normal expression level in the control group (Table 8 and Figs. 12 and 13). Moreover, the obtained results revealed that compound **3i** exhibited the potential to decrease the normal expression level of PARP-1 by 33% in a comparable force to the inhibitory potential of doxorubicin (41%) (Table 8 and Figs. 12 and 13).

**Table 8:** Effects of compound **3i** and doxorubicin on the protein expression level of Bax, caspases 3 activity and on PARP-1 inhibition in comparison to untreated LOX IMVI cell line as negative control.

Compound	Caspace-3	Bax	PARP-1
	Optical density (OD)	OD	OD
3i/ LOX IMVI	3.14	4.13	0.87
Doxorubicin/ LOX IMVI	5.67	6.56	0.38
Control/ LOX IMVI	1	1	1

#### Screening of antibacterial activities

The in vitro antibacterial screening of the final compounds **3a-l** was assessed against Gram-positive *S. aureus ATCC 6538 strain* and Gram-negative strains, *K. pneumoniae ATCC 10,031*, *P. aeruginosa ATCC 27,853 and E. coli ATCC 25,922* using standard agar cup diffusion method^37^ in comparison to ciprofloxacin as antibacterial reference and results were listed as the minimum concentration that inhibited bacterial growth (Table 9).

**Table 9:** Minimum inhibitory concentrations (MIC, µM) for the new target compounds **3a-l** in addition to the parent *ciprofloxacin* against *S. Aureus ATCC 6538*,* E. Coli* (ATCC 25922), *K. pneumoniae ATCC 10,031 and P. Aeruginosa ATCC 27,853.*

Compound	Bacterial strain			
	Gram + ve strains	Gram –ve strains		
	S. aureus ATCC 6538	E. coli ATCC 25,922	K. pneumoniae ATCC 10,031	*P*. aeruginosa ATCC 27,853
**3a**	> 20	**0.20**	0.22	> 20
**3b**	> 20	0.32	**0.04**	> 20
**3c**	> 20	0.48	0.38	> 20
**3d**	> 20	0.20	0.13	0.87
**3e**	> 20	1.94	0.19	> 20
**3f**	> 20	2.23	> 20	> 20
**3 g**	> 20	0.25	0.25	> 20
**3 h**	> 20	**0.04**	0.80	> 20
**3i**	> 20	0.54	> 20	> 20
**3j**	> 20	2.60	> 20	> 20
**3k**	> 20	0.25	0.66	> 20
**3 L**	> 20	> 20	> 20	> 20
**Ciprofloxacin**	5.49	**0.04**	**0.10**	**0.06**

According to the MICs depicted in Table 9, it is clear that ciprofloxacin derivatives, unlike ciprofloxacin, showed no pronounced activity against Gram-negative *p. aeruginosa ATCC 27,853* or Gram-positive strain *S. aureus ATCC 6538.* Only compound **3 h** exhibited comparable activity to that of ciprofloxacin against *E. coli* both with MIC of 0.04 µM. While compounds **3e**, **3f**, and **3j** was of much lower activity with MICs of 1.94–2.60 µM. Screening against *K. pneumoniae ATCC 10,031* elicited that compound 3b was more potent than ciprofloxacin with an MIC of 0.04 compared to 0.1 µM, while the ciprofloxacin derivatives **3f**,** 3i**,** 3j**, and **3 L** was devoid of that activity. Compounds **3a**, **3c**-**3e**,** 3 g–3 h**, and **3k** was of moderate activity against *K. pneumoniae ATCC 10,031* with MICs of 0.13–0.80 µM which are of slightly lower potency compared to parent ciprofloxacin which achieved MIC of 0.10 µM.

#### Physicochemical properties and ADME prediction

The Swiss Institute of Bioinformatics (SIB) provides the SwissADME online tool, which is publicly accessible. This tool combines many computational methods to provide a thorough evaluation of the pharmacokinetics profile and drug-like properties of small compounds^38^. The tool available at http://www.swissadme.ch. The technique was used to determine the possible effectiveness and suitability of the newly created compounds in terms of their pharmacokinetics. The hybrid (**3i**) with the highest level of activity had a logPo/w value of 3.85, indicating moderate hydrophobicity, and a topological polar surface area (TPSA) of 145.5 Å with 8 hydrogen bond acceptor groups and just 1 donor group.

**Fig. 14:**
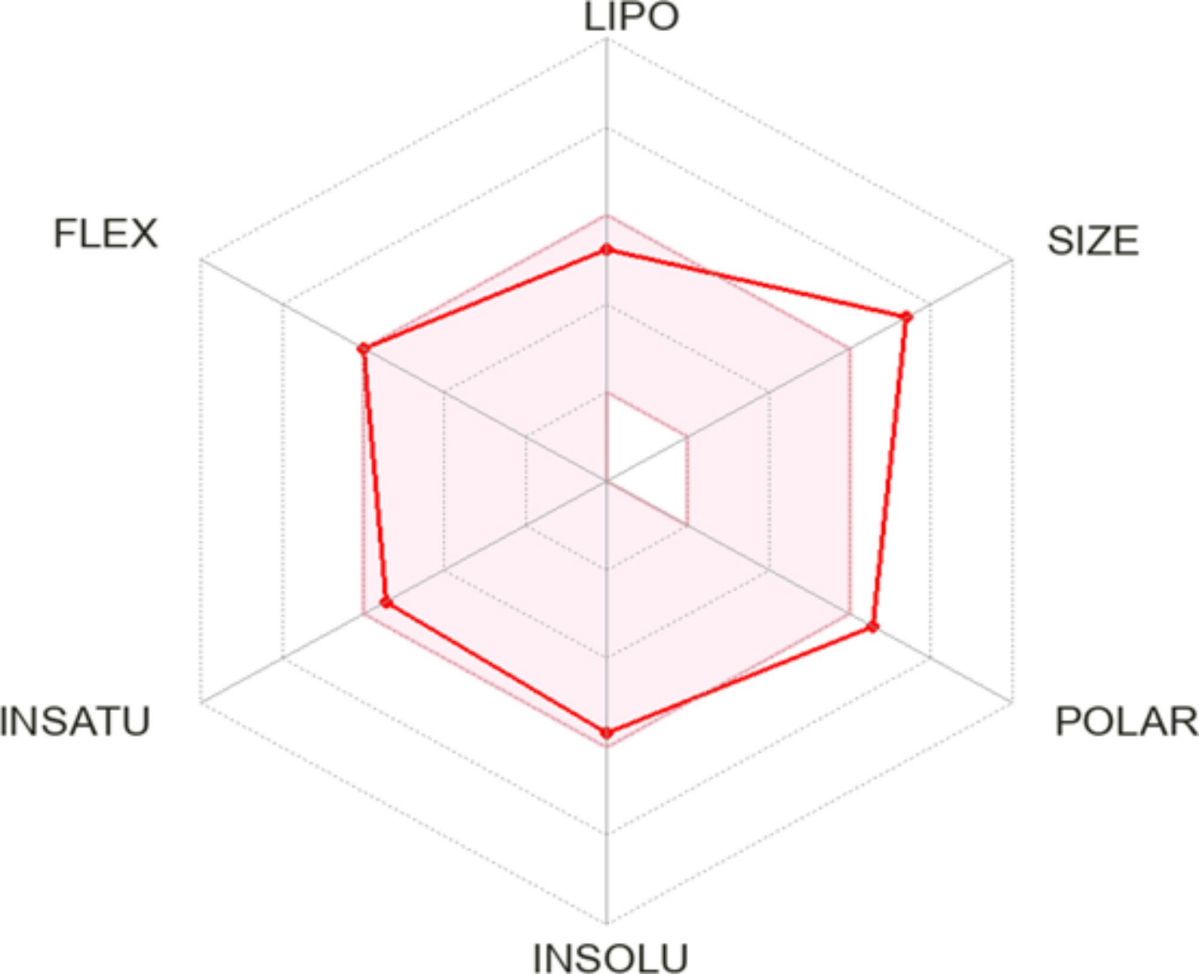
Swiss ADME online tool’s bioavailability radar graph for compound **3i**. The range of optimal property values for oral bioavailability is shown by the pink region, while the expected characteristics of **3i** are indicated by the red lines.

The tested chemical 3i has a predicted GIT availability score of 0.56. Figure 14 displays the bioavailability radar map for compounds **3i**. The radar plot consists of six axes representing six crucial characteristics for oral bioavailability: saturation (INSATU), flexibility (FLEX), lipophilicity (LIPO), size (SIZE), polarity (POLAR), and solubility (INSOLU)^39^. The pink region shows the range of ideal property values, whereas the red line represents compounds **3i**. Compounds **3i** have predicted attributes that are almost entirely inside the pink area, suggesting their high expected oral bioavailability. The SwissADME online web tool indicated that the ciprofloxacin/thiazolidine hybrid **3i** largely meets the criteria for drug-likeness set by major pharmaceutical companies. It only violates one criterion, which is the high molecular weight according to Lipinski’s (Pfizer)^40^ filter, and demonstrates a violation of 5 degrees higher than the 140 topological surface area defined by Veber’s (GSK)^41^ filter. This suggests that the recently created compounds exhibit favourable pharmacokinetic qualities and desirable drug-like characteristics. It also provides a foundation for developing comparable derivatives that completely meet the necessary pharmacokinetic parameters.

### Discussion

Medicinal chemists now widely use hybridization as a well-known strategy to design potential targets for complex ailments like cancertopo^17,36^. The capacity of hybridization to tackle several targets by one inhibitor was beneficial in such a context to permit better efficiency and lower toxicity and thus potentially replace combination therapy^42^. Having this in mind, the authors used the hybridization technique to join two potentially active scaffolds in the design of compounds **3a**-**l**. Extensive alkylation or acylation of the piperazine ring joins ciprofloxacin with other fragments. Both tools were employed to modify the structure of ciprofloxacin to enhance its antibacterial and/or anticancer activity^43,44^. The two used fragments; ciprofloxacin and thiazolidin-2,4-dione; held strong support in the literature for their anti-cancer activity^17,29–31,36^. The two fragments were successfully linked together through a butyryl linker, forming compounds **3a-l** using acetylation of the piperazine ring as a tool to add the thiazolidine ring.

The formed hybrids inaugurated the design hypothesis as they showed moderate anti-cancer activity against the 60 cell lines panel tested by NCI (Table 1**)**, with the best activity observed against Renal cancer A498, Renal cancer UO-31, CNS Cancer SNB-75. The introduction of a phenyl group at the active methylene of the thiazolidine-2,4-dione moiety of compound **3 L** by Knoevenagel condensation in compound **3a** resulted in potentiating the anti-proliferative activity against melanoma LOX IMVI, melanoma SK-MEL-5 cell lines. It is worth mentioning that the introduction of electron withdrawing or donating groups on the phenyl moiety has no significant impact on the anti-proliferative activity of the target compounds **3a-l** and compound **3i**, with the smallest substitution having comparable activity as unsubstituted **3a** with an IC_50_ of 25.4 ± 1.43$\:{\upmu\:}\text{M}$ compared to 26.7 ± 1.50 $\:{\upmu\:}\text{M}$ for **3a** against LOX IMVI cell line, while being more active against A498 cells with an IC_50_ of 33.9 ± 1.91 $\:{\upmu\:}\text{M}$ compared to more than 150 $\:{\upmu\:}\text{M}$ for **3a**. With all other substituents inducing decreased activity, these data imply that electronic factors probably do not contribute to the observed activity, while the size of substitution must be very small for optimal activity. Interestingly, previous reports of joining the sulfur-containing sulfadiazole ring on the carboxylic acid group of ciprofloxacin showed similar anti-cancer activity on a different spectrum of cell lines, such as MCF 7 and A549^45^.

Since ciprofloxacin makes up most of the hybrids that were made, topoisomerase inhibition seems like a good way to explain the anti-cancer activity that was seen. Besides, topoisomerases I/II are excellent targets for developing novel compounds with anticancer activity, especially compounds containing the quinolone moiety, which have well known topoisomerase enzyme inhibitory activity^36^. The study of the topoisomerase inhibitory activity of hybrids containing ciprofloxacin has always been a rational mechanistic step in previous literature^46^. Thus, in vitro testing against both enzymes supported those facts, with compound **3i** having the highest anti-cancer activity and being the most potent against Topo I and II with 4.77 ± 0.26 and 15 ± 0.81, supporting the hypothesis that topoisomerase inhibition is the major mechanism contributing to the anti-cancer activity. Furthermore, data suggests Topo I is more affected than Topo II. Docking also endorsed the achieved activity, as the study revealed a potential ability of compound **3i** to form more stable complexes with the active site than that of ciprofloxacin. A lower energy score was seen with the complex formed with the active site of Topo I than that formed with Topo II supporting the in vitro data that revealed Topo I is more affected by the tested compounds than Topo II. The phenyl ring substitution on the thiazolidine ring participated in a pi-cation interaction with amino acids found in Topo active site (Figs. 4 and 8) explaining the enhanced activity observed in compounds **3a** and **3i** compared to **3 L**. Moreover, the presence of the fluorine atom slightly out of the active site of both Topo I/II (indicated by the blue highlight on the fluorine atom in both Figs. 4 and 8 suggesting that the size of the substitution is essential in those group of compounds as increased size will not probably fit into the active site of both enzymes and explains the decreased activity observed with other substitutions such as methyl, nitro and naphthyl groups.

The anti-cancer activity is usually chaperoned with both cell cycle arrest and apoptosis. The cell cycle closely controls proliferation and differentiation processes^47^. An examination of the cell sequence implied G1, S, G2, and M as the four stages of the cell cycle. The cell enlarges in the G1 phase, and the DNA becomes ready for duplication. The second stage (S phase) initiates DNA replication, while the third stage (G2) involves the repair of new DNA. Finally, in the fourth stage (M), the nuclear division occurs^48^. Therefore, cell cycle arrest can effectively inhibit the proliferation of cancer cells. An accumulation of cells treated with compound **3i** in the S phase revealed cell cycle arrest induction at the S phase. A result that was reasonable and goes with the literature reporting that ciprofloxacin is inducing cell cycle arrest at the same stage^49^.

Moreover, compound **3i** was able to induce apoptosis. Apoptosis is a regulated process that leads to cell self-destruction. Any defects in the apoptosis process result in malignancy and increased resistance to cancer chemotherapy. Cancer disease is associated with too little apoptosis^50^. The ability of **3i** to induce apoptosis was ascertained by both cell accumulation at the pre-G phase in cell cycle analysis and by the annexitin stain analysis of LOX IMVI cells treated with compound **3i**.

For further investigation of the mechanism that compound **3i** uses to induce apoptotic protein levels were measured, including caspase 3, Bax, and PARP-1. Caspases have an important role in apoptosis, as if caspases were activated during the initial stages of apoptosis, the cleavage of crucial cellular components, including structural proteins are usually triggered. Caspase activation also led to activation caspase 3 activity^36^. The ability of compound **3i** to intensify caspase 3 levels to about 20 folds more than control indicates a significant role for caspase 3 in **3i**-induced apoptosis. The association of both pharmacophores utilised in **3i** with caspase 3 upof other enzymes inducing DNA cleavage^36^. Malignancies typically impair -regulation entirely rationalizes the high caspase 3 levels obtained, as both ciprofloxacin^51^ and thiazolidinedione^25^ are reported to provoke caspase 3 levels, eliciting apoptosis.

The ability of **3i** to promote apoptosis was also supported by the elevated levels of Bax observed. Bax acts as an apoptotic activator, being a part of the Bcl-2 gene family. It induces a mitochondrial voltage-dependent anion channel (VDAC) opening, initiating membrane potential disruption with cytochrome c release. It is a significant component of P53-mediated apoptosis^52^. Again, the association of ciprofloxacin with such a mechanism^54^ completely explains the increased level of Bax associated with **3i**.

Poly (ADP-ribose) polymerase-1 (PARP-1) is a nuclear repair enzyme. PARP-1 blockers usually result in destabilized replication of DNA due to the formation of PARP-1 DNA entrapment, which results in replication stress-induced mitotic catastrophe and cell death. Therefore, researchers have developed PARP inhibitors as chemotherapy sensitizers for cancer treatment^26^. Structure based drug design has identified thiazolidine-2,4-dione as potential inhibitors for PARP-1 enzyme^55^ thus the ability of **3i** to inhibit PARP-1 was evaluated. **3i** elicited a downregulation of about a third of PARP-1 found in LOX IMVI cells, contributing to the apoptosis of such cells.

Based on the above-mentioned results, the anticancer activity of compound **3i** can be emphasized against LOX IMVI, which is associated with apoptosis induction and cell cycle arrest.

Furthermore, due to ciprofloxacin’s pronounced anti-bacterial activity, the antibacterial activity of compounds **3a-l** was analyzed. Data disclosed that hybridization of ciprofloxacin with thiazolidine-2,4-dione *via* butyryl linker mainly abolishes activity against Gram-positive strain *S. aureus ATCC 6538* as the Minimum inhibitory concentrations (µM) of all target compounds **3 a-l** is > 20 µM compared to ciprofloxacin (MIC = 5.49). Similarly, it abolished the activity against *P. aeruginosa* ATCC 27,853. The activity observed with the other two tested Gram-negative strains, E. coli ATCC 25,922 and *K. pneumoniae* ATCC 10,031 with less than that observed with the parent ciprofloxacin, with very few exceptions, such as compound **3 h** which was equipotent to ciprofloxacin against *E. coli* and compound **3b**, which was highly active against K. pneumoniae ATCC 10,031.

The current research assumes a significant role for the type of linker used for the denoted activity. Our research group previously designed hybrids of the two pharmacophores; ciprofloxacin and thiazolidinedione, through a short acetyl linker. The obtained hybrids showed strong anti-bacterial activity, with barely any anti-cancer activity observed^34^. Further research is being undertaken to fully explain the impact of the linker on such hybrids.

## Conclusion

The current study of ciprofloxacin/thiazolidine hybrids reported the synthesis of the designed hybrids using a butyryl hybrid. The synthesized compounds highlighted the significance of the used linker in hybrid design. The introduction of a butyryl linker in connecting thiazolidine and ciprofloxacin shifted the activity of the synthesized compounds toward anti-cancer activity rather than anti-bacterial one. A promising anti-cancer activity was observed with the highest activity observed against the human melanoma LOX IMVI cancer cell line. An unsubstituted phenyl group (**3a)** or one carrying a very small electronegative substitution on the thiazolidine ring (**3i)** appears optimum for activity against LOX IMVI cancer cell line with IC_50_ 26.7 ± 1.50 and 25.4 ± 1.43 µM, respectively. More importantly, compound **3i** showed lower toxicity toward normal cell line W138 in comparison to doxorubicin with IC_50_ values of 45.13 ± 2.43 and 18.13 ± 0.98, respectively. Mechanistically, the most active compound 3i showed inhibition for both topoisomerase I and II with IC_50_ values of 4.77 ± 0.26 and 15.00 ± 0.81, respectively in addition to the induction of apoptosis and blockade of the cell cycle at S phase. Docking study was in accordance with biological experiments supporting the ability of the compounds to inhibit topoisomerase enzymes. Hence, such outcomes could establish the basis for utilizing both ciprofloxacin and thiazolidine in future studies for the design of targeted anti-cancer agents and further validate the activity of such compounds in defeating cancer cell lines.

## Experimental

### Chemistry

#### General

Reactions were tracked using TLC on aluminum pre-coated silica gel (2 cm, x 5 cm, Kieselgel 60, Merk) plates using methylene chloride 95: methanol 5 V/V as eluent. Spots were identified via illumination to a UV lamp at a wavelength of 254 nm. Uncorrected melting values were found using an electrothermal melting point device from Stuart Scientific Co. The Faculty of Science at Sohag University uses a Shimaduz 408 instrument Spectrophotometer to record IR spectra as KBr discs. The Faculty of Science at Sohag University and Faculty of Pharmacy, Mansoura University and used a Bruker AM NMR (400 MHz) spectrometer to capture NMR spectra. All numbers referring to NMR data obtained are in parts per million (ppm) using tetramethylsilane (TMS) as reference slandered. At the Regional Centre for Mycology and Biotechnology, Al-Azhar University, Cairo, Egypt, elemental microanalyses of the synthesized compounds for carbon, nitrogen, and hydrogen were conducted using APCI as the ion source. Mass spectroscopy was performed at the Nawah Scientific Centre in Cairo, Egypt.

#### General procedure of the synthesis of compounds 1a-k

To a mixture of TZD (0.234 g, 2 mmol) and the appropriate aromatic aldehyde (2 mmol), sodium acetate (0.410 g, 5 mmol) was added. The whole mixture then heated at reflux in glacial acetic acid for 10–12 h. Then the reaction mixture was emptied into crushed ice and the formed solid was filtered, washed with distilled water and recrystallized from methanol to obtain pure compounds **1a-k** in E-configuration which emphasized by their reported melting points^34,36^. the compounds **1a-k** confirmed by comparing to the reported melting point in addition to their ^1^H NMR (as shown in supplementary data).

**(*E*)-5-benzylidenethiazolidine-2**,**4-dione 1a**.

White crystals; yield: 0.177 g (87%); mp: 248–250 °C; (reported: 248–250 °C)^34^, ^56^.

**(*E*)-5(2-methyl benzylidene)thiazolidine-2**,**4-dione 1b**.

Brown crystals; yield: 0.189 g (87.8%); mp: 186–187 °C; (reported: 184–185 °C)^34^.

**(*E*)-5(4-methyl benzylidene)thiazolidine-2**,**4-dione 1c**.

Yellowish crystals; yield: 0.198 g (91%); mp: 227–228 °C; (reported: 225–226 °C)^34,57^.

**(*E*)-5(3-nitro benzylidene)thiazolidine-2**,**4-dione 1d**.

Yellowish white crystals; yield: 0.209 g (84.7%); mp; 188–189 °C; (reported: 187–188 °C)^34,57^.

**(*E*)-5(4-methoxybenzylidene)thiazolidine-2**,**4-dione 1e**.

White crystals; yield: 0.188 g (80%); mp: 217–220 °C; (reported: 217–218 °C)^34,57^.

**(*E*)-5(3**,**4-dimethoxybenzylidene)thiazolidine-2**,**4-dione 1f**.

Yellow crystals yield: 0.191 g (72%); mp: 210–211 °C; (reported: 208–210 °C)^34,57^.

**(*E*)-5(3**,**4**,**5-trimethoxybenzylidene)thiazolidine-2**,**4-dione 1 g**.

Yellow crystals; yield: 0.222 g (75%); mp: 207–209 °C; (reported: 210–213 °C)^34,58^.

**(*E*)-5(4-chlorobenzylidene)thiazolidine-2**,**4-dione 1 h**.

White crystals; yield: 0.184 g (80%); mp: 228–230 °C; (reported: 226–228 °C)^34,59^.

**(*E*)-5 (4-fluorobenzylidene)thiazolidine-2**,**4-dione 1i**.

White crystals; yield: 0.230 g (77%); mp: 222–223 °C; (reported: 223–224 °C)^34,57^.

**(*E*)-5-((naphthalene-1-yl) methylene)thiazolidine-2**,**4-dione 1j**.

Pale yellow crystals; yield: 0.195 g (89.5%); mp: 285–286 °C; (reported: 285–287 °C)^34^.

**(*E*)-5 (4-nitrobenzylidene)thiazolidine-2**,**4-dione 1k**.

Orange crystals; yield: 0.215 g (86.2%); mp: 261–262 °C; (reported: 261–263 °C)^34,60^.

#### Procedure for the synthesis of buytryl ciprofloxacin 2

To a stirred solution of ciprofloxacin (1 mmol) in DCM (25 mL) at 0–5 °C. A solution of potassium carbonate (1.5 mmol) in distilled water (25 mL) was added. Chlorobutyryl chloride (1.15 mmol) in methylene chloride (25 mL) was added over a period of 20 min. Stirring was continued for 2 h at 0–5 °C, then at room temperature for extra 12 h. Then the organic layer separated, washed with distilled water (3 × 20 mL), 1 N HCl (3 × 20 mL) and distilled water (3 × 20 mL), dried over sodium sulfate anhydrous, flittered and finally dried to afford compound **2**^59,61^.

#### 7-(4-(4-chlorobutryl)piperazin-1-yl)-1-cyclopropyl-6-fluoro-1,4-dihydro-4-oxoquinoline-3-carboxylic acid 2

White powder; yield: 0.386 g (88.50%); mp: 261–262 °C, IR (KBr) ύ (cm^−1^): 1715 (carboxylic acid C = O), 1685 (amidic C = O), 1625 (C4- C = O); ^1^H-NMR (400 MHz, DMSO-*d*_*6*_) δ ppm: 1.21–1.27 (2 H, m, cyclopropyl-H), 1.32–1.36 (2 H, m, cyclopropyl-H), 2.03–2.05 (2 H, m, Br–CH_2_C*H*_*2*_–CH_2_), 2.55 (2 H, m, *J*_*H−F*_ = 8.0 Hz, Cl–CH_2_CH_2_C*H*_*2*_CO), 3.32–3.36 (4 H, m, Piperazine-H), 3.69 (2 H, t, *J*_*H−F*_ = 8.0 Hz, Cl–C*H*_2_CH_2_CH_2_CO), 3.71–3.77 (4 H, m, Piperazine-H), 3.81–3.87 (1H, m, cyclopropyl-H), 7.62 (1H, d, *J*_*H−F*_ = 6.6 Hz, H-8), 7.89(1H, d, *J*_*H−F*_ = 13.8 Hz, H-5), 8.66 (1 H, s,H-2), 15.11 (1 H, s, COO*H*); ^13^C-NMR (100 MHz, DMSO-*d*_*6*_): 8.06, 22.19, 28.41, 36.33, 42.95, 44.94, 45.48, 49.63, 50.02, 106.99, 107.29, 111.54, 119.16, 139.58, 144.59, 149.54, 154.35, 165.53, 171.33, 176.51.

### General procedure for the synthesis of compounds 3a-l

A mixture of butyryl ciprofloxacin **2** (0.109 g, 0.25 mmol), thiazolidine-2,4-dione (for synthesis of **3 L**) or the appropriate derivative of 5-benzylidenethiazolidine-2,4-diones **1 a-k** (0.25 mmol) (for synthesis of **3 a-k)** and potassium hydroxide (0.50 mmol) in DMF (5 mL) was refluxed for 8–12 h. then, the reaction mixture added to crushed ice; the formed precipitate was filtered, washed with distilled water (3 × 25 mL), dried and finally crystallized from acetonitrile to afford target compounds **3a-l**^34^.

#### 7-[4-[4-[5-benzylidene-2,4-dioxo-thiazolidin-3-yl]butanoyl]piperazin-1-yl]-1-cyclopropyl-6-fluoro-4-oxo-quinoline-3-carboxylic acid 3a

White powder; yield: 0.114 g (76%); mp: 238 °C; IR (KBr) ύ (cm^−1^): 1742 (thiazolidine-2, 4-dione C = O), 1717 (carboxylic acid C = O), 1693 (amidic C = O), 1649 (thiazolidine-2, 4-dione C = O), 1628 (4-keto C = O) and 883 (C-S) ; ^1^H-NMR (400 MHz, DMSO-*d*_*6*_) ppm: 1.17–1.23 (2 H, m, cyclopropyl-H), 1.29–1.37 (2 H, m, cyclopropyl-H), 1.90–1.96 (2 H, m, -CH_2_*CH*_*2*_CH_2_-CO), 2.48 (2 H, t, *J* = 6 Hz, -CH_2_CH_2_C*H*_2_-CO), 3.32–3.38 (4 H, m, Piperazine-H), 3.75 (2 H, t, *J* = 6 Hz, N -C*H*_2_CH_2_ CH_2_-CO), 3.77–3.81 (4 H, m, piperazine-H), 3.82–3.86 (1H, m, cyclopropyl-H), 7.50–7.56 (4 H, m, Aromatic-H), 7.58–7.60 (1H, m, Aromatic-H), 7.62 (1H, d, *J*_H−F_ = 7.0 Hz, H-8 ), 7.88 (1H, s, olefinic-H), 7.92 (1H, d, *J*_H−F_ = 12.8 Hz, H-5), 8.67 (1H, s, H-2), 14.99 (1H, s, COOH); ^13^C-NMR (100 MHz, DMSO-*d*_*6*_): 8.05, 23.04, 29.87, 36.30, 41.49, 44.93, 44.96, 49.66, 49.86, 106.91, 107.39, 111.54 (d, *J* = 23 Hz), 119.25, 122.16, 127.16, 129.81, 130.48, 131.00, 131.60, 133.06, 133.53, 139.67, 145.39, 148.47, 153.38, 166.29, 167.92, 170.42, 170.75, 176.86; Anal. Calcd for C_31_H_29_FN_4_O_6_S: C, 61.58; H, 4.83; N, 9.27; S, 5.18 Found: C, 61.75; H, 4.81; N, 9.26; S, 5.20. MS (APCl) calcd for C_31_H_26_FN_4_ K_2_O_6_S [M + 2 K-3 H]^−^: 679.1, found: 679.2.

#### 1-cyclopropyl-6-fluoro-7-[4-[4-[5-(m-tolyl)methylene]-2,4-dioxo-thiazolidin-3-yl]butanoyl]piperazin-1-yl]-4-oxoquinoline-3-carboxylic acid 3b

Yellow crystals; yield 0.074 g (48%); mp: 2206 –227 °C ; IR (KBr) ύ (cm^−1^): 1742 (thiazolidine-2, 4-dione C = O), 1717 (carboxylic acid C = O), 1693 (amidic C = O), 1649 (thiazolidine-2, 4-dione C = O), 1628 (4-keto C = O) and 884 (C-S); ^1^H-NMR (400 MHz, DMSO-*d*_*6*_) δ ppm:1.16–1.18 (2 H, m, cyclopropyl-H), 1.21–1.27 (2 H, m, cyclopropyl-H), 1.86–1.91 (2 H, m, -CH_2_*CH*_*2*_CH_2_-CO), 2.39 (3 H, s, C*H*_3_-), 2.47 (2 H, t, *J* = 6 Hz, -CH_2_CH_2_C*H*_2_-CO), 3.27–3.34 (4 H, m, Piperazine-H), 3.68 (2 H, t, *J* = 6 Hz, N-C*H*_2_CH_2_ CH_2_-CO), 3.71–3.79 (4 H, m, Piperazine-H), 3.80–3.85 (1H, m, cyclopropyl-H), 7.35–7.40 (3 H, m, Aromatic-H), 7.40–7.44 (1H, m, Aromatic-H), 7.57 (1H, d, *J*_H−F_ = 7.0 Hz, H-8), 7.89 (1H, d, *J*_H−F_ = 14.0, H5), 8.00 (1H, s, -olefinic-H), 8.59 (1H, s, H2), 14.95 (1H, s, COOH); ^13^CNMR (100 MHz, DMSO-*d*_*6*_): 8.05, 19.77, 23.03, 29.84, 36.32, 41.85, 44.93, 49.64, 49.94, 106.97, 107.31, 111.53 (d, *J* = 23 Hz), 119.32, 123.80, 127.13, 127.65, 130.86, 130.93, 130.93, 131.46, 132.63, 139.09, 145.42, 148.51, 152.24, 166.02, 166.34, 168.20, 170.40, 176.80; Anal. Calcd for C_32_H_31_FN_4_O_6_S: C, 62.12; H, 5.05; N, 9.06; S, 5.18 Found: C, 61.89; H, 5.04; N, 9.08; S, 5.16.

#### 1-cyclopropyl-6-fluoro-7-[4-[4-[5-(p-tolyl)methylene]-2,4-dioxo-thiazolidin-3-yl]butanoyl]piperazin-1-yl]-4-oxoquinoline-3-carboxylic acid 3c

White powder; yield 0.101 g (66%); mp: 233–234 °C; IR (KBr) ύ (cm^−1^):1735 (thiazolidine-2, 4-dione C = O), 1713 (carboxylic acid C = O), 1683 (amidic C = O), 1650 (thiazolidine-2, 4-dione C = O), 1624 (4-keto C = O) and 886 (C-S); ^1^H-NMR (400 MHz, DMSO-*d*_*6*_) δ ppm:1.16–1.20 (2 H, m, cyclopropyl-H), 1.29–1.34 (2 H, m, cyclopropyl-H), 1.89–1.94 (2 H, m, -CH_2_*CH*_*2*_CH_2_-CO), 2.37 (3 H, s, C*H*_3_-), 2.46 (2 H, t, *J* = 6.4 Hz, -CH_2_CH_2_C*H*_2_-CO), 3.30–3.39 (4 H, m, Piperazine-H), 3.63–3.71 (4 H, m, Piperazine-H), 3.73 (2 H, t, *J*_H−F_ = 6.4 Hz, N -C*H*_2_CH_2_ CH_2_-CO), 3.80–3.85 (1H, m, cyclopropyl-H), 7.34 (2 H, d, *J* = 8.0 Hz, Aromatic-H), 7.49 (2 H, d, *J* = 8.0 Hz, Aromatic-H), 7.56 (1H, d, *J*_H−F_ = 7.0 Hz, H-8), 7.86 (1H, s, N-C*H*=), 7.92 (1H, d, *J*_H−F_ = 14.0 Hz, H-5), 8.66 (1H, s, H2), 14.95 (1H, s, COOH);^13^C NMR (100 MHz, DMSO-*d*_*6*_): 8.05, 21.53, 23.03, 29.82, 36.33, 41.85, 44.92, 49.65, 49.90, 106.93, 107.30, 111.50 (d, *J* = 23 Hz), 119.20, 120.84, 130.44, 130.57, 130.74, 133.14, 139.62, 141.36, 145.39, 148.46, 153.37 (d, *J* = 250 Hz), 165.02, 166.32, 167.80, 167.95, 170.39, 176.83; Anal. Calcd for C_32_H_31_FN_4_O_6_S: C, 62.12; H, 5.05; N, 9.06; S, 5.18 Found: C, 62.35; H, 5.06; N, 9.03; S, 5.20.

#### 1-cyclopropyl-6-fluoro-7-[4-[4-[5-[(3-nitrophenyl)methylene]-2,4-dioxo-thiazolidin-3-yl]butanoyl]piperazin-1-yl]-4-oxoquinoline-3-carboxylic acid 3d

White powder; yield: 0.118 g (73%); mp: 263 °C; IR (KBr) ύ (cm^−1^): 1735 (thiazolidine-2, 4-dione C = O), 1715 (carboxylic acid C = O), 1689 (amidic C = O), 1649 (thiazolidine-2, 4-dione C = O), 1625 (4-keto C = O) and 888 (C-S); ^1^H-NMR (400 MHz, DMSO-*d*_*6*_) δ ppm:^1^H-NMR (400 MHz, DMSO-*d*_*6*_) δ ppm: 1.17–1.23 (2 H, m, cyclopropyl-H), 1.30–1.35 (2 H, m, cyclopropyl-H), 1.89–1.92 (2 H, m, -CH_2_C*H*_2_CH_2_-CO), 2.48 (2 H, d, *J* = 6 Hz,-CH_2_ CH_2_ C*H*_2_-CO), 3.33–3.40 (4 H, m, Piperazine-H), 3.65–3.71 (4 H, m, Piperazine-H), 3.77 (2 H, t, *J* = 6 Hz, N-C*H*_2_ CH_2_ CH_2_-CO), 3.80–3.85 (1H, m, cyclopropyl-H) 7.56 (1H, d, *J*_H−F_ = 7.0 Hz, H-8), 7.83(1H, t, *J* = 8.0 Hz, Aromatic-H), 7.92 (1H, d, *J*_H−F_ = 13.2 Hz, H5), 8.02 (1H, d, *J* = 8.0 Hz, Aromatic-H), 8.06 (1H, s, Aromatic-H ), 8.29 (1H, d, *J* = 8 Hz, Aromatic-H), 8.44 (1H, s, -olefinic-HC), 8.66 (1H, s, H2), 14.98 (1H, s, COOH); ^13^C-NMR (100 MHz, DMSO-*d*_*6*_): 8.05, 22.99, 29.86, 36.30, 42.12, 44.32, 44.93, 49.65, 49.88, 106.89, 107.34, 111.50 (d, *J* = 23 Hz), 119.21, 124.84, 125.01, 125.20, 130.66, 131.40, 135.23, 135.84, 139.63, 145.37, 148.44, 148.80 153.36 (d, *J* = 252 Hz), 165.95, 166.30, 167.35, 170.42, 176.83; Anal. Calcd for C_31_H_28_FN_5_O_8_S: C, 57.31; H, 4.34; N, 10.78; S, 4.94 Found: C, 57.12; H, 4.33; N, 10.74; S, 4.92; MS (APCl) calcd for C_31_H_29_FN_5_O_8_S [M + H]^+^: 650.2, found: 649.50.

#### 1-cyclopropyl-6-fluoro-7-[4-[4-[5-[(4-methoxyphenyl)methylene]-2,4-dioxo-thiazolidin-3-yl]butanoyl]piperazin-1-yl]-4-oxoquinoline-3-carboxylic acid 3e

Yellow powder; yield: 0.109 g (69%); mp: 244 –243 °C; IR (KBr) ύ (cm^−1^): 1746 (thiazolidine-2, 4-dione C = O), 1715 (carboxylic acid C = O), 1690 (amidic C = O), 1654 (thiazolidine-2, 4-dione C = O), 1626 (4-keto C = O) and 888 (C-S); ^1^H-NMR (400 MHz, DMSO-*d*_*6*_) δ ppm: 1.15–1.20 (2 H, m, cyclopropyl-H), 1.30–1.36 (2 H, m, cyclopropyl-H), 1.88–1.92 (2 H, m, -CH_2_*CH*_*2*_CH_2_-CO), 2.46 (2 H, t, *J* = 6.4 Hz, -CH_2_CH_2_C*H*_2_-CO), 3.33–3.37 (4 H, m, Piperazine-H), 3.67 (2 H, t, *J* = 6.4 Hz, N-C*H*_2_CH_2_ CH_2_-CO), 3.70–3.75 (4 H, m, Piperazine-H), 3.80–3.84 (1H, m, cyclopropyl-H), 3.84 (3 H, s, OC*H*_*3*_), 7.09 (2 H, d, *J* = 8 Hz, Aromatic-H), 7.55 (2 H, d, *J* = 8 Hz, Aromatic-H), 7.56 (1H, d, *J*_H−F_ = 7.0 Hz, H-8), 7.85 (1H, s, N-C*H*=), 7.92 (1H, d, *J*_H−F_ = 14.0 Hz, H-5), 8.65 (1H, s, H2), 15.08 (1H, s, COOH); ^13^C-NMR (100 MHz, DMSO-*d*_*6*_): 8.05, 23.08, 29.83, 36.31, 41.79, 44.92, 44.94, 49.63, 49.89, 56.00, 106.89, 107.34, 111.50 (d, *J* = 23 Hz), 115.44, 115.54, 118.81, 119.21, 125.97, 132.61, 133.12, 139.63, 145.37, 148.43, 153.37 (d, *J* = 247 Hz), 161.62, 166.36, 167.96, 170.39, 176.83; Anal. Calcd for C_32_H_31_FN_4_O_7_S: C, 60.56; H, 4.92; N, 8.83; S, 5.05. Found: C, 60.66; H, 4.90; N, 8.80; S, 5.06.

#### 1-cyclopropyl-7-[4-[4-[5-(3,4-dimethoxyphenyl)methylene]-2,4-dioxo-thiazolidin-3-yl]butanoyl]piperazin-1-yl]-6-fluoro-4-oxo-quinoline-3-carboxylic acid 3f

Yellow powder; yield: 0.106 g (64%); mp: 241 °C ; ^1^H-NMR (400 MHz, DMSO-*d*_*6*_) δ ppm: 1.17–1.21 (2 H, m, cyclopropyl-H), 1.28–1.35 (2 H, m, cyclopropyl-H), 1.90–1.93 (2 H, m, -CH_2_*CH*_*2*_CH_2_-CO), 2.48 (2 H, t, *J* = 6.4 Hz, -CH_2_CH_2_C*H*_2_-CO), 3.23–3.27 (4 H, m, Piperazine-H), 3.29–3.37 (4 H, m, 4-Piperazine-H), 3.74 (2 H, t, *J* = 6.4 Hz, N -C*H*_2_CH_2_ CH_2_-CO), 3.80–3.84 (1H, m, cyclopropyl-H), 3.84 (6 H, s, OC*H*_3_ ), 7.12 (1H, d, *J* = 8 Hz, Aromatic-H), 7.19 (1H, d, *J* = 8 Hz, Aromatic-H), 7.55 (1H, d, *J*_H−F_ = 8.0 Hz, H-8), 7.85 (1H, s, -olefinic-H), 7.92 (1H, d, *J*_H−F_ = 13.8 Hz, H5), 8.65 (1H, s, H2), 15.08 (1H, s, COOH); ^13^C-NMR (100 MHz, DMSO-*d*_*6*_*)*: 8.04, 23.09, 29.82, 36.29, 41.79, 44.82, 44.94, 49.64, 49.91, 56.13, 56.24, 106.87, 107.35, 111.51 (d, *J* = 22 Hz), 112.74, 114.00, 118.99, 119.21, 124.30, 126.21, 133.49, 139.63, 145.33 (d, *J* = 9 Hz), 148.43, 149.55, 151.53, 153.37 (d, *J* = 249 Hz), 166.30, 166.31, 167.96, 170.39 and 176.83; Anal. Calcd for C_33_H_33_FN_4_O_8_S: C, 59.63; H, 5.00; N, 8.43; S, 4.82. Found: C, 59.71; H, 5.01; N, 8.41; S, 4.81. MS (APCl) calcd for C_33_H_32_FK_2_N_4_O_8_S [M + 2 K-3 H]^−^: 739.1, found: 738.5.

#### 1-cyclopropyl-7-[4-[4-[2,4-dioxo-5-(3,4,5-trimethoxyphenyl) methylene] thiazolidin-3-yl]butanoyl]piperazin-1-yl]-6-fluoro-4-oxo-quinoline-3-carboxylic acid 3 g

Yellow powder; yield: 0.135 g (77%); mp: 252 °C; 1 H-NMR (400 MHz, DMSO-*d*_*6*_) δ ppm: 1.17–1.22 (2 H, m, cyclopropyl-H), 1.28–1.33 (2 H, m, cyclopropyl-H), 1.88–1.92 (2 H, m, -CH2C*H*_2_CH_2_-CO), 2.47 (2 H, t, *J* = 6.4 Hz, -CH_2_ CH_2_C*H*2-CO), 3.30–3.35 (4 H, m, Piperazine-H), 3.65–3.72 (4 H, m, 4-Piperazine-H), 3.74 (2 H, t, *J* = 6.4 Hz, N -C*H*_2_CH_2_CH_2_-CO), 3.74 (3 H, s, OCH_3_ ), 3.80–3.83 (1 H, m, cyclopropyl-H), 3.84 (6 H, s, OC*H*3), 6.95 (2 H, s, Aromatic-H ), 7.58 (1 H, d, *J*_*H−F*_ = 7.2 Hz, H2), 7.88 (1 H, s, -olefinic-H), 7.89 (1 H, d, *J*_*H−F*_ = 12 Hz, H5), 8.59 (1 H, s, H2), 15.22 (1 H, s, COOH); ^13^C-NMR (100 MHz, DMSO-*d*_*6*_): 8.04, 22.09, 28.82, 37.29, 41.88, 44.91, 44.96, 49.92, 56.61, 60.71, 106.92, 107.35, 108.28, 111.41 (d, *J* = 22 Hz), 119.29, 121.0, 128.97, 133.37, 139.69, 140.26, 145.06, 148.45, 149.55, 153.37, 166.03, 166.31, 167.88, 170.41 and 176.84; Anal. Calcd for C_34_H_35_FN_4_O_9_S: C, 58.78; H, 5.08 N, 8.06; S, 4.82. Found: C, 58.95; H, 5.09; N, 8.05; S, 4.81.

#### 1-cyclopropyl − 7-[4-[4-[5-(4-chlorophenyl)methylene]-2,4-dioxo-thiazolidin-3-yl] butanoyl] piperazin-1-yl] -6-fluoro-4-oxoquinoline-3-carboxylic acid 3 h

White powder; yield: 0.094 g (59%); mp: 273 °C; ^1^H-NMR (400 MHz, DMSO-*d*_*6*_) δ ppm: 1.15–1.22 (2 H, m, cyclopropyl-H), 1.30–1.36 (2 H, m, cyclopropyl-H), 1.92 (2 H, m, -CH_2_*CH*_*2*_CH_2_-CO), 2.47 (2 H, t, *J* = 6.4 Hz, -CH_2_CH_2_C*H*_2_-CO), 3.30–3.40 (4 H, m, Piperazine-H), 3.65–370 (4 H, m, Piperazine-H), 3.75 (2 H, t, *J* = 6.4 Hz, N -C*H*_2_CH_2_ CH_2_-CO), 3.80–3.85 (1H, m, cyclopropyl-H), 7.57 (1H, d, *J*_H−F_ = 7.0 Hz, H-8), 7.60 (2 H, d, *J* = 8 Hz, Aromatic-H), 7.64 (2 H, d, *J* = 8 Hz, Aromatic-H), 7.91(1H, s, N-C*H*=), 7.98 (1H, d, *J*_H−F_ = 14.0 Hz, H-5), 8.66 (1H, s, H2), 15.13 (1H, s, COOH); 8.03, 22.94, 29.81, 36.31, 41.94, 44.99, 49.58, 49.83, 106.88, 107.25, 111.51 (d, *J* = 23 Hz), 119.24, 122.90, 129.89, 131.72, 132.12, 132.36, 135.65, 39.64, 145.41, 148.50, 153.38 (d, *J* = 247 Hz), 166.19, 166.51, 167.67, 170.52 and 176.86; Anal. Calcd for C_31_H_28_FClN_4_O_6_S: C, 58.26; H, 4.42; N, 8.77; S, 5.02. Found: C, 58.06; H, 4.41; N, 8.79; S, 5.01; MS (APCl) calcd for C_31_H_28_ Cl_3_FN_4_O_6_S [M + 2Cl]^−^: 708.1, found: 707.5.

#### 1-cyclopropyl-6-fluoro-7-[4-[4-[5-(4-fluorophenyl)methylene]-2,4-dioxo-thiazolidin-3-yl]butanoyl]piperazin-1-yl]-4-oxoquinoline-3-carboxylic acid 3i

White powder; yield: 0.101 g (65%); mp: 272 °C; 1 H-NMR (400 MHz, DMSO-*d*_*6*_) δ ppm: 1.16–1.23 (2 H, m, cyclopropyl-H), 1.30–1.35 (2 H, m, cyclopropyl-H), 1.94 (2 H, m, -CH_2_C*H*_2_CH_2_-CO), 2.48 (2 H, d, *J* = 6 Hz,-CH_2_ CH_2_ C*H*_2_-CO), 3.32–3.39 (4 H, m, Piperazine-H), 3.65–3.71 (4 H, m, Piperazine-H), 3.74 (2 H, t, *J* = 6 Hz, N -C*H*_2_ CH_2_CH_2_-CO), 3.82–3.85 (1 H, m, cyclopropyl-H), 7.38 (2 H, t, *J*_H−H =_ 8 Hz, Aromatic-H), 7.57 (1 H, d, *J*_H−F_ = 7.0 Hz, H-8), 7.68 (2 H, t, *J*_H−H =_ 8 Hz, Aromatic-H), 7.89 (1 H, s, N-C*H*=), 7.92 (1 H, d, *J*_H−F_ = 13.2 Hz, H5), 8.66 (1 H, s, H2), 15.08 (1 H, s, COOH); ^13^C-NMR (100 MHz, DMSO-d6): 8.05, 23.05, 29.88, 36.30, 41.95, 44.94, 49.69, 49.89, 106.88, 107.38, 111.52 (d, *J* = 23 Hz), 116.86, 117.08, 119.24, 121.87, 130.20, 131.98, 132.91, 139.65, 145.27, 148.42, 153.36 (d, *J =* 247 Hz), 164.67, 166.24, 167.77, 170.42 and 176.86; Anal. Calcd for C_31_H_28_F_2_N_4_O_6_S: C, 59.80; H, 4.53; N, 9.00; S, 5.15. Found: C, 59.83; H, 4.52; N, 9.02; S, 5.14; MS (APCl) calcd for C_31_H_27_F_2_N_4_O_6_S [M-H]^−^: 621.2, found: 621.6.

#### 1-cyclopropyl-6-fluoro-7-[4-[4-[5-(1-naphthylmethylene)-2,4-dioxo-thiazolidin-3-yl]butanoyl]piperazin-1-yl]-4-oxo-quinoline-3-carboxylic acid 3j

White powder; yield: 0.098 g (60%); mp: 219 °C; ^1^H-NMR (400 MHz, DMSO-*d*_*6*_) δ ppm: 1.14–1.20 (2 H, m, cyclopropyl-H), 1.28–1.32 (2 H, m, cyclopropyl-H), 1.96 (2 H, m, -CH_2_*CH*_*2*_CH_2_-CO), 2.49 (2 H, t, *J*_H−F_ = 6.4 Hz, -CH_2_CH_2_C*H*_2_), 3.32–3.42 (4 H, m, Piperazine-H), 3.70 (2 H, t, *J*_H−F_ = 6.4 Hz, N -C*H*_2_CH_2_ CH_2_-CO), 3.72–3.78 (4 H, m, Piperazine-H), 3.80–3.83 (1H, m, cyclopropyl-H), 7.54 (1H, d, *J*_H−F_ = 7.0 Hz, H-8),7.62–7.71 (4 H, m, Aromatic-H), 7.92 (1H, d, *J*_H−F_ = 14.0 Hz, H-5), 8.02–8.11 (3 H, m, Aromatic-H), 8.53 (1H, s, N-C*H*=) 8.64 (1H, s, H2), 15.08 (1H, s, COOH), ^13^C-NMR (100 MHz, DMSO-d6): ^13^C-NMR (100 MHz, DMSO-d6): 8.03, 23.02, 29.20, 36.28, 41.90, 44.96, 49.66, 106.90, 107.32, 111.52 (d, J = 23 Hz), 119.31, 121.85, 123.83, 125.69, 126.09, 126.85, 127.31, 127.92, 129.36, 129.99, 130.71, 131.36, 133.77, 139.62, 145.40, 148.49, 153.29 (d, J = 247 Hz), 165.85, 166.45, 168.21, 170.42 and 176.67; Anal. Calcd for C_35_H_31_FN_4_O_6_S: C, 64.21; H, 4.77; N, 8.56; S, 4.90. Found: C, 64.04; H, 4.77; N, 8.53; S, 4.90.

#### 1-cyclopropyl-6-fluoro-7-[4-[4-[5-(4-nitrophenyl)methylene]-2,4-dioxo-thiazolidin-3-yl]butanoyl]piperazin-1-yl]-4-oxoquinoline-3-carboxylic acid 3k

Yellow powder; yield: 0.110 g (68%); mp: 299–300 °C; ^1^H -NMR (400 MHz, DMSO-*d*_*6*_) ppm: 1.16–1.20 (2 H, m, cyclopropyl-H), 1.28–1.33 (2 H, m, cyclopropyl-H), 1.93 (2 H, m ,-CH_2_C*H*_2_CH_2_-CO), 2.48 (2 H, t, *J* = 7.0 Hz,-C*H*_2_ CH_2_ C*H*_2_-CO), 3.32–3.39 (4 H, m, Piperazine-H), 3.68–3.74 (4 H, m, Piperazine-H), 3.76 (2 H, t, *J* = 6 Hz, N -C*H*_2_ CH_2_ CH_2_-CO), 3.80–3.84 (1H, m, cyclopropyl-H), 7.56 (1H, d, *J*_H−F_ = 7.0 Hz, H-8), 7.83 (2 H, d, *J* = 8 Hz, Aromatic-H), 7.92 (1H, d, *J*_H−F_ = 14.0 Hz, H-5), 8.03 (2 H, d, *J* = 8 Hz, Aromatic-H), 8.44 (1H, s, -olefinic-H), 8.66 (1H, s, H2), 15.20 (1H, s, COOH), ^13^C-NMR (100 MHz, DMSO-*d*_*6*_) δ: 8.04, 27.76, 29.814, 36.23, 42.75, 44.93, 45.48, 49.59, 50.02, 106.89, 107.19, 111.55 (d, *J* = 23 Hz), 119.31, 122.95, 129.88, 130.73, 133.67, 132.21, 135.21, 139.62, 145.29, 148.51, 153.32 (d, *J*_*C−F*_ = 252 Hz), 166.19, 167.67, 169.52, 173.52 and 176.86; Anal. Calcd for C_31_H_28_FN_5_O_8_S: C, 57.31; H, 4.34; N, 10.78; S, 4.94 Found: C, 57.16; H, 4.35; N, 10.81; S, 4.93; MS (APCl) calcd for C_31_H_29_FN_5_O_8_S [M + H]^+^: 650.2, found: 650.1.

#### 1-Cyclopropyl-7-(4-(4-(2,4-dioxothiazolidin-3-yl)butanoyl)piperazin-1-yl)-6-fluoro-4-oxo-1,4-dihydroquinoline-3-carboxylic acid 3 L

White powder; yield: 0.070 g (54%); mp: 230 °C; ^1^H-NMR (400 MHz, DMSO-*d*_*6*_) δ ppm: 1.03–1.15 (2 H, m, cyclopropyl-H), 1.26–1.33 (2 H, m, cyclopropyl-H), 1.73 (2 H, m ,-CH_2_C*H*_2_CH_2_-CO), 2.11 (2 H, t, *J*_H−F_ = 7.0 Hz,-C*H*_2_ CH_2_ C*H*_2_-CO), 2.35 (2 H, t, *J*_H−F_ = 6.4 Hz, -COCH_2_CH_2_C*H*_2_), 2.49 (2 H, S, C*H*_*2*_), 3.35–343 (4 H, m, Piperazine-H), 3.57-63 (4 H, m, Piperazine-H), 3.56–3.63 (4 H, m, Piperazine-H), 3.78–3.82 (1H, m, cyclopropyl-H), 7.54 (1H, d, *J*_H−F_ = 7.0 Hz, H-8), 7.84 (1H, d, *J*_H−F_ = 13.2 Hz, H5), 8.87 (1H, s, H2), 15.08 (1H, s, COOH); ^13^C-NMR (100 MHz, DMSO-d6): 7.01, 22.33, 29.15, 32.90, 40.60, 43.38, 45.63, 45.70, 48.68, 48.75, 104.00, 105.93, 110.36, 122.98, 140.54, 142.00, 148.15, 152.99, 169.13, 170.95, 171.76, 173.70 and 174.99; Anal. Calcd for C_24_H_25_FN_4_O_6_S: C, 55.81; H, 4.88; N, 10.85; S, 6.21. Found, 55.64; H, 4.88; N, 10.81; S, 6.19.

### Pharmacological screening

#### Screening of anti-cancer activity in national cancer institute (NCI)

The anti-cancer potential of the tested compounds was evaluated at the National cancer institute (NCI), USA, against 9 panels of 60 various cell lines derived from nine human tumours available usually at NCI library. The procedures for the screening were described in details in NCI website (dttp://www.dtp.nci.nih.gov.) and were done according to NCI protocols^60 ^. The anticancer screening was carried out at single dose of 10^−5^ M, NCI, Bethesda, USA. The results obtained as growth inhibition (%).

#### Evaluation of IC_50_ of compounds 3a,** b**,** 3i**,** 3j**, **3 L** against LOX IMVI human melanoma and A498 renal cancer cell lines

The IC_50_ of compounds **3a-b**, **3i-l** against LOX IMVI, A498 and W138 cell lines was calculated using a reported MTT assay protocols^62^. All cell lines used were purchased from vacsera cell culture library, Tissue Culture Unit, Cairo, Egypt, with ATCC origin. Tissue Culture Unit. The concentrations resulting in inhibition of 50% cell growth were executed for three times and the mean was calculated.

Cells were cultured using DMEM (Invitrogen/Life Technologies) supplemented with 10% FBS (Hyclone, ), 10 mg/ml of insulin (Sigma), and 1% penicillin-streptomycin. Plate cells (cells density 1.2–1.8 × 10,000 cells/well) in a volume of 100mL complete growth medium + 100 mL of the tested compound per well in a 96-well plate for 24 h before the MTT assay. In a typical experiment, 100 mL of serial 10-fold diluted sterile tested compounds were added to final concentrations of 0.01e100 mM using culture media as negative control. After 24 h of culture incubation and supernatants discarded. LOX IMVI and A498 cell lines were trypsinized and washed with Ca/Mg free PBS (pH 7.2). We removed cultures from incubator into laminar flow hood or other sterile work area. Cells in the log phase of growth should be employed and final cell number should not exceed 106 cells/cm2. Each test should include a blank containing complete medium without cells. Reconstitute each vial of MTT [M-5655] to be used with 3 ml of medium or balanced salt solution without phenol red and serum. Add reconstituted MTT in an amount equal to 10% of the culture medium volume. Return cultures to incubator for 2–4 h depending on cell type and maximum cell density. After the incubation period, remove cultures from incubator and dissolve the resulting formazan crystals by adding an amount of MTT Solubilization Solution [M-8910] equal to the original culture medium volume. Spectrophotometrically absorbance was measured at a wavelength of 570 nm. Measure the background absorbance of multi-well plates at 690 nm and subtract from the 450 nm measurement. Results from all experiments were recorded and the percentage of viable cells was calculated.

#### Evaluation of topoisomerase I/II inhibition

Compounds **3a-b** and **3i-l** were selected for topoisomerases inhibition assay in comparison to cisplatin and doxorubicin using the Elisa kit of human DNA topoisomerase following the described protocols^17^.

Compounds 3a-b, 3i-l, cisplatin and doxorubicin was evaluated for topoisomerase Iα and topoisomerase IIβ inhibitory activity utilizing the human DNA topoisomerase Elisa kit. Two folds of serial dilution was accomplished after standards and the tested compounds were dissolved in sample diluent. Horseradish peroxidase (HRP-avidin) and biotin-conjugated antibody were diluted 10 times each. Each well received 100 µL of each concentration of the standard or test chemicals, which were then added and incubated at 37 °C for 60 min. After each well’s liquid had been carefully removed, 100 µL of a solution of biotin-conjugated antibody had been added, and each well had been incubated for 1 h at 37 °C. The microtiter plate was rinsed three times and given room to aspirate. After that, the plate was incubated at 37 °C for 60 min with 100 µL of HRP-avidin solution added to each well. After that, the plate was aspirated and cleaned 5 times. Each well received 90 µL of TMB substrate, and the plate was incubated for 30 min. at 37 °C in a light-protected environment. Finally, stop solution (50 µL) was loaded, and within 5 min, the absorbance at 450 nm was measured spectrophotometrically.

#### Cell cycle analysis and measurement of apoptotic potential

The effect of compound **3i** on cell cycle progression of LOX IMVI cell line was evaluated using Propidium Iodide Flow Cytometry Kit to measure the DNA content according to the reported protocols^25^. The LOX IMVI cells used in this work were obtained from the American Type Culture Collection. Cells were cultured in DMEM (Invitrogen/Life Technologies) supplemented with 10% FBS (Hyclone), 10 µg/ml insulin (Sigma), and 1% penicillin-streptomycin. The remaining chemicals and reagents were all from Sigma or Invitrogen. Growth media was removed, cells washed with warm PBS and aspirated. Adhered cells were detached from media by addition of 5 ml 10% trypsin/EDTA and incubated 2 min in the incubator. The suspended cells harvested, aspirated to a falcon tube, centrifuged at 2000 rpm for 5 min. Cell count performed after suspending the cells into a fresh media and stained with trypan blue, counted and used for preparation of the appropriate number of cells.

LOX IMVI cells were treated by the IC_50_ concentration of compound **3i (**25.4 ± 1.43). The influence of compound **3i** to induce apoptosis in LOX IMVI cell line was estimated in comparison to the conventional doxorubicin and untreated cells as positive and negative controls, respectively. Following the manufacturer’s instructions, the Annexin V-FITC Apoptosis Detection Kit (Bio Vision Research Products, USA) was used to analyze cell apoptosis. Briefly, 500 µL of 1X Binding buffer was used to resuspend 1-5 × 10^5^ cells that had been harvested by centrifugation. Propidium iodide (PI, 50 mg/ml) and Annexin V-FITC, each administered in 5 µL, were also added. The cells were first incubated for 5 minutes at room temperature in the dark before being analyzed using the Annexin V-FITC binding flow cytometric technique (Ex = 488 nm; Em = 530 nm) with a FITC signal detector (typically FL1) and PI staining with a phycoerythrin emission signal detector (typically FL2). Before exposing adherent cells to Annexin V-FITC, we gently trypsinized and gave them a single wash in serum-containing medium (A.3–5). Cell synchronisation is done using Double-thymidine synchronization method and the used Media is double modifed eagle media (DMEM).

#### Western plotting analysis of Bax, PARP-1 and caspase-3 levels

To evaluate the expression level of the apoptosis modulatory markers Bax, caspase 3 and PARP-1, western blot was performed as formerly described^26^. Three different groups of LOX IMVI cells were permitted to grow to reach 70–80% confluence in 75 cm^3^ flasks. The first group of LOX IMVI cells was treated with compound 3i. The second group was treated with doxorubicin as a positive control. The third group was treated with the growth medium without any active pharmacophore to serve as a negative control. The media was taken out after 48 h, and cells underwent three rounds of ice-cold PBS washing. RIPA lysis buffer [consisting of Tris-HCl pH 7.6 (25 mM), NaCl (150 mM), EDTA (5 mM), NP-40 or Triton X-100 (1%), sodium deoxycholate (1%), SDS1 (1%)] with the proper phosphatase and a protease inhibitor was used to create the whole-cell lysate for 30 min. After centrifuging the mixture at 12,000 rpm for 20 min and sonicating it for five minutes, the supernatant was collected, split into aliquots, and kept at -80 °C. The Biuret method was used to determine protein content^27,28^. Proteins in each matched cell lysate were denatured in 2 Laemmli buffer containing 5% β--mercaptoethanol at 95 °C for 5 min. For SDS-PAGE electrophoresis, 20 µg of protein per lane were loaded at 100 volts through a 6% stacking gel, then at 125 volts through a 10% resolving gel for around 2 h, and then transferred to a PVDF membrane using a semidry transfer apparatus (made by Cleaver Scientific) for 25 min. The PVDF membrane was first incubated for one hour at 4 °C in TBS buffer containing 0.1% Tween (TBST) and 5% fat-free milk, then overnight at 4 °C with monoclonal anti-Bax, anti-PARP-1, and anti-caspase-3 as primary antibodies (Cell Signalling Technology) at a dilution of 1:500. Membranes were first treated overnight, then three times washed in TBST buffer, then incubated at room temperature for 60 min with the corresponding HRP (horse radish peroxidase)-linked secondary antibodies (Dako). Then the substrate for the chemiluminescent Western ECL (Perkin Elmer, Waltham, MA) was added to the blot as described by the manufacturer by incubating the membranes with equal volumes of ECL solution A and ECL solution B for 1 min. A CCD camera-based imager (Chemi Doc imager, Biorad, USA) eas then employed to capture the chemiluminescent signals, and Image Lab (Biorad) was used to evaluate bands intensities. Semi-quantified of the detected bans using ImageJ/NIH software (National Institute of mental health, Bethesda, Maryland, USA) was used for statistical analysis. It was verified that each lane had equivalent protein loading by stripping and reblotting each membrane against a monoclonal anti-ß-actin antibody(Sigma) at 4 °C at a dilution of 1:500 [65] (for details, see supplementary data).

#### Anti-bacterial activity screening

Anti-bacterial activity of the recently synthesized compounds **3a**-**k** was evaluated at AUMC (Assiut University Mycological Centre), Assiut, Egypt. Assays were done against Gram-negative strains of *K. pneumoniae (ATCC 100310*,* E. coli (ATCC 25922)*,* Pseudomonas aeruginosa (ATCC 27853)* and the Gram-positive *S. aureus (ATCC 6538) strain* utilizing standard agar cup diffusion method.

The isolates from the four bacterial species examined were kept in TSB (Trypticase Soya Broth, Becton and Dickinson) with 10% glycerol at at -70 °C and then subcultured on TSA (Trypticase Soya Agar, Becton and Dickinson) and TSB for 24 h at 37 °C former to inoculation.

Bacterial isolates were then plated on sterile petri plates with 1 × 10^8^ CFU/mL (0.5 McFarland turbidity) and 20 mL of Mueller Hinton Agar media (Oxoid) was then loaded. To ensure that the microorganisms were evenly dispersed, the plates were gently rotated before being left to harden on a level surface. Four equally spaced spherical-shaped wells (10 mm diameter) were painstakingly drilled using a sterilized cork bore after media solidification. All tested compounds under test underwent two-fold serial dilutions in a sequential manner. Three replications of 100 µL of each concentration were added to a well using a micropipette. Plates were then incubated for 24-hour at 37 ºC. The average of the inhibition zones was then calculated. To determine the MICs, the natural logarithm of each diluted test substance’s concentration was plotted against the average area of the related inhibition zone. Following that, a regression line was created and the intercept on the ‘ln concentration” axis was determined, and the antilogarithm was calculated to indicate the MIC value^34,63^.

## Electronic supplementary material

Below is the link to the electronic supplementary material.

Supplementary Material 1

## Data Availability

The authors declare that the data supporting the findings of this study are available within the paper and its Supplementary Information files.
