# Supplementary material for: Design, synthesis and mechanistic study of N-4-Piperazinyl Butyryl Thiazolidinedione derivatives of ciprofloxacin with Anticancer Activity via Topoisomerase I/II inhibition
Source: Sci Rep. 2024 Oct 15;14:24101. doi: 10.1038/s41598-024-73793-y (PMC11480511; doi:10.1038/s41598-024-73793-y)
Supplement: Supplementary file 1 — Supplementary Material 1 [file 41598_2024_73793_MOESM1_ESM.pdf]

Supporting information

Design, Synthesis and Mechanistic Study of N-4-Piperazinyl Butyryl Thiazolidinedione Derivatives of Ciprofloxacin with Anticancer Activity via Topoisomerase I/II Inhibition.

Hossameldin A. Aziz[1,2] , Ahmed M. El-Saghier[3], Mohamed badr[4], Bakheet E. M. Elsadek[5], Gamal El-Din A. Abuo-Rahma[2,6]*, Mai E. Shoman*[2]

[1]Department of Pharmaceutical Chemistry, Faculty of Pharmacy, New Valley University, New Valley, Egypt
[2]Department of Medicinal Chemistry, Minya University, Minia, Egypt
[3]Department of Chemistry, Faculty of Science, Sohag University, Sohag, Egypt
[4]Department of Biochemistry, Faculty of Pharmacy, Menoufia University, Menoufia, Egypt
[5]Department of Biochemistry, Faculty of pharmacy, Al-Azhar University, Assiut, Egypt
[6]Department of Pharmaceutical Chemistry, Deraya University, New Minia, Minia, Egypt

*Correspondence:
Gamal El-Din A. Abuo-Rahma, e-mail: gamal.abourahma@mu.edu.eg, Mobile: +201003069431.
Mai E. Shoman, e-mail: Mai_shoman@mu.edu.eg, Mobile: +201116757931.

Figure S 1: 1HNMR spectrum of compound 1a (400 MHz, CDCl3)	4
Figure S 2: 1HNMR spectrum of compound 1b (400 MHz, CDCl3)	4
Figure S 3: 1HNMR spectrum of compound 1c (400 MHz, DMSO-d6)	5
Figure S 4: 1HNMR spectrum of compound 1d (400 MHz, DMSO-d6)	5
Figure S 5: 1HNMR spectrum of compound 1e (400 MHz, CDCl3)	6
Figure S 6: 1HNMR spectrum of compound 1f (400 MHz, CDCl3)	6
Figure S 7: 1HNMR spectrum of compound 1g (400 MHz, CDCl3)	7
Figure S 8: 1HNMR spectrum of compound 1h (400 MHz, DMSO-d6)	7
Figure S 9: 1HNMR spectrum of compound 1i (400 MHz, CDCl3)	8
Figure S 10: 1HNMR spectrum of compound 1j (400 MHz, CDCl3)	8
Figure S 11: 1HNMR spectrum of compound 1k (400 MHz, CDCl3)	9
Figure S 12: 1HNMR spectrum of compound 2 (400 MHz, DMSO-d6)	9
Figure S 13: 13CNMR spectrum of compound 2 (100 MHz, DMSO-d6)	10
Figure S 14: IR of compound 2	10
Figure S 15: 1HNMR spectrum of compound 3a (400 MHz, DMSO-d6)	11
Figure S 16: 13CNMR spectrum of compound 3a (100 MHz, DMSO-d6)	11
Figure S 17: IR of compound 3a	12
Figure S 18: 1HNMR spectrum of compound 3b (400 MHz, DMSO-d6)	12
Figure S 19: 13CNMR spectrum of compound 3b(100 MHz, DMSO-d6)	13
Figure S 20: IR of compound 3b	13
Figure S 21: 1HNMR spectrum of compound 3c (400 MHz, DMSO-d6)	14
Figure S 22: 13CNMR spectrum of compound 3c(100 MHz, DMSO-d6)	14
Figure S 23: IR of compound 3c	15
Figure S 24: 1HNMR spectrum of compound 3d (400 MHz, DMSO-d6)	15
Figure S 25: 13CNMR spectrum of compound 3d(100 MHz, DMSO-d6)	16
Figure S 26: IR of compound 3d	16
Figure S 27: 1HNMR spectrum of compound 3e (400 MHz, DMSO-d6)	17
Figure S 28: 13CNMR spectrum of compound 3e(100 MHz, DMSO-d6)	17
Figure S 29: IR of compound 3e	18
Figure S 30: 1HNMR spectrum of compound 3f (400 MHz, DMSO-d6)	18
Figure S 31: 13CNMR spectrum of compound 3f(100 MHz, DMSO-d6)	19
Figure S 32: 1HNMR spectrum of compound 3g (400 MHz, DMSO-d6)	19
Figure S 33: 13CNMR spectrum of compound 3g(100 MHz, DMSO-d6)	20
Figure S 34: 1HNMR spectrum of compound 3h aliphatic region (400 MHz, DMSO-d6)	20
Figure S 1: 1HNMR spectrum of compound 3h aromatic region (400 MHz, DMSO-d6)	21
Figure S 36: 13CNMR spectrum of compound 3h(100 MHz, DMSO-d6)	21
Figure S 37: 1HNMR spectrum of compound 3i (400 MHz, DMSO-d6)	22
Figure S 38: 13CNMR spectrum of compound 3i(100 MHz, DMSO-d6)	22
Figure S 39: 1HNMR spectrum of compound 3j (400 MHz, DMSO-d6)	23
Figure S 40: 13CNMR spectrum of compound 3j (100 MHz, DMSO-d6)	23
Figure S 41: 1HNMR spectrum of compound 3k (400 MHz, DMSO-d6)	24
Figure S 42: 1HNMR spectrum of compound 3l (400 MHz, CF3COOH)	24
Figure S 43: 13CNMR spectrum of compound 2l (100 MHz, CF3COOH)	25
Figure S 44: One dose growth (%) and mean graph for compound 2	25
Figure S 45: One dose growth (%) and mean graph for compound 3a	26
Figure S 46: One dose growth (%) and mean graph for compound 3b	27
Figure S 47: One dose growth (%) and mean graph for compound 3c	28
Figure S 48: One dose growth (%) and mean graph for compound 3d	29
Figure S 49: One dose growth (%) and mean graph for compound 3e	30
Figure S 50: One dose growth (%) and mean graph for compound 3f	31
Figure S 51: One dose growth (%) and mean graph for compound 3g	32
Figure S 52: One dose growth (%) and mean graph for compound 3h	33
Figure S 53: One dose growth (%) and mean graph for comound 3i	34
Figure S 54: One dose growth (%) and mean graph for compound 3j	35
Figure S 55: One dose growth (%) and mean graph for compound 3k	36
Figure S 56: One dose growth (%) and mean graph for compound 3l	37

**Figure S57: IC 50 of compounds 3a, 3e, 3i, 3j, 3k, 3l, cisplatin and doxorubicin against LOX-IMVI and A 498 cancer cell lines**	**40**
**Table S1: Calculation of IC50 of compounds 3a and 3j against LOX-IMVI**	**41**
**Table S2: Calculation of IC50 of compounds 3i and 3e against LOX-IMVI**	**41**
**Table S3: Calculation of IC50 of compounds 3k and 3lagainst LOX-IMVI**	**42**
**Table S4: Calculation of IC50 of compounds cisplatin and doxorubicin against LOX-IMVI**	**42**
**Table S5: Calculation of IC50 of compounds 3a and 3j against A498 cell line**	**43**
**Table S6: Calculation of IC50 of compounds 3i and 3e against A498 cell line**	**44**
**Table S7: Calculation of IC50 of compounds 3k and 3l against A498 cell line**	**44**
**Table S8: Calculation of IC50 of compounds cisplatin and doxorubicin against A498 cell line**	**45**
**Table S9: Topoisomerase I/II inhibitory activity by compounds 3a-3b, 3i-l, cisplatin and doxorubicin**	**46**
**Figure S 58: topoisomerase I inhibition induced by compounds 3a (BB), 3b (B2CH3), 3i (BF), 3j (BN), 3k (BPNO2), 3l (BTZD), cisplatin and doxorubicin (conc. in µg/mL)**	**47**
**Figure S59: topoisomerase II inhibition induced by compounds 3a (BB), 3b (B2CH3), 3i (BF), 3j (BN), 3k (BPNO2), 3l (BTZD), cisplatin and doxorubicin (conc. in µg/mL)**	**47**
**Table S10: the cell cycle analysis of Melanoma LOX IMVI cell treated with compound 3i, doxorubicin against negative control**	**48**
**Table S11: the apoptosis assay of Melanoma LOX IMVI cell treated with IC50 concentration of compound 3i, doxorubicin against negative control**	**48**
**Figure S60: the apoptosis and necrosis assay of Melanoma LOX IMVI induced by IC50 concentration of compound 3i, doxorubicin against negative control**	**48**
**Table S12: Effects of compound 3i on the protein expression level of Bax, caspases 3 activity and on PARP-1 inhibition**	**51**
**Figure S61: Effects of compound 3i (BF3) on the protein expression level of Bax, PARP-1 and caspases 3 activity in LOX IMVI cell line**	**51**
**Figure S64:** 2D and 3D illustration of **etoposide** docked into the active site of topoisomerase II enzyme (PDB: 3QX3).	**53**

1-Chemistry

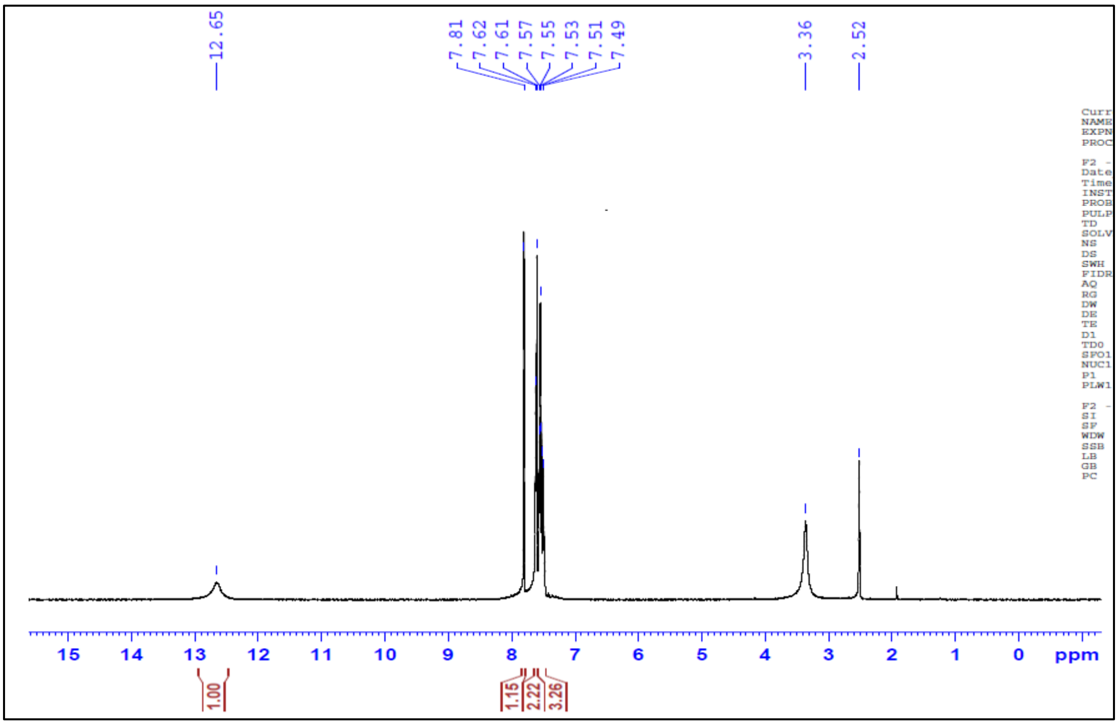

Figure S1: ^{1}HNMR spectrum of compound **1a** (400 MHz, $CDCl_3$).

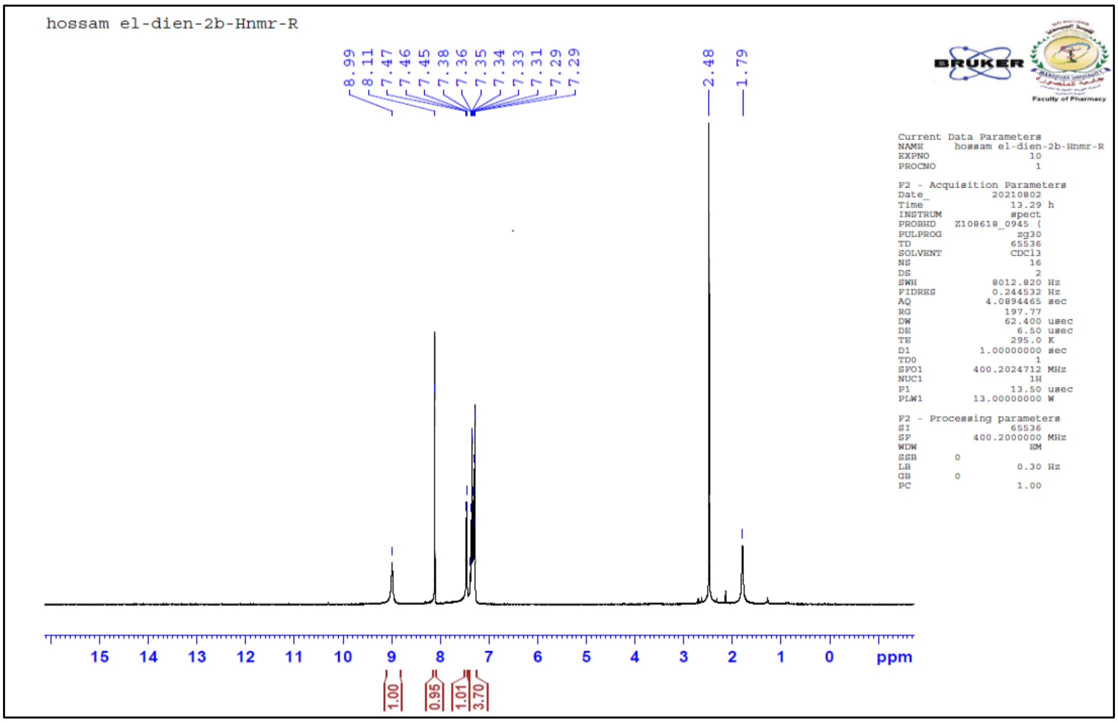

Figure S2: ^{1}HNMR spectrum of compound **1b** (400 MHz, $CDCl_3$).

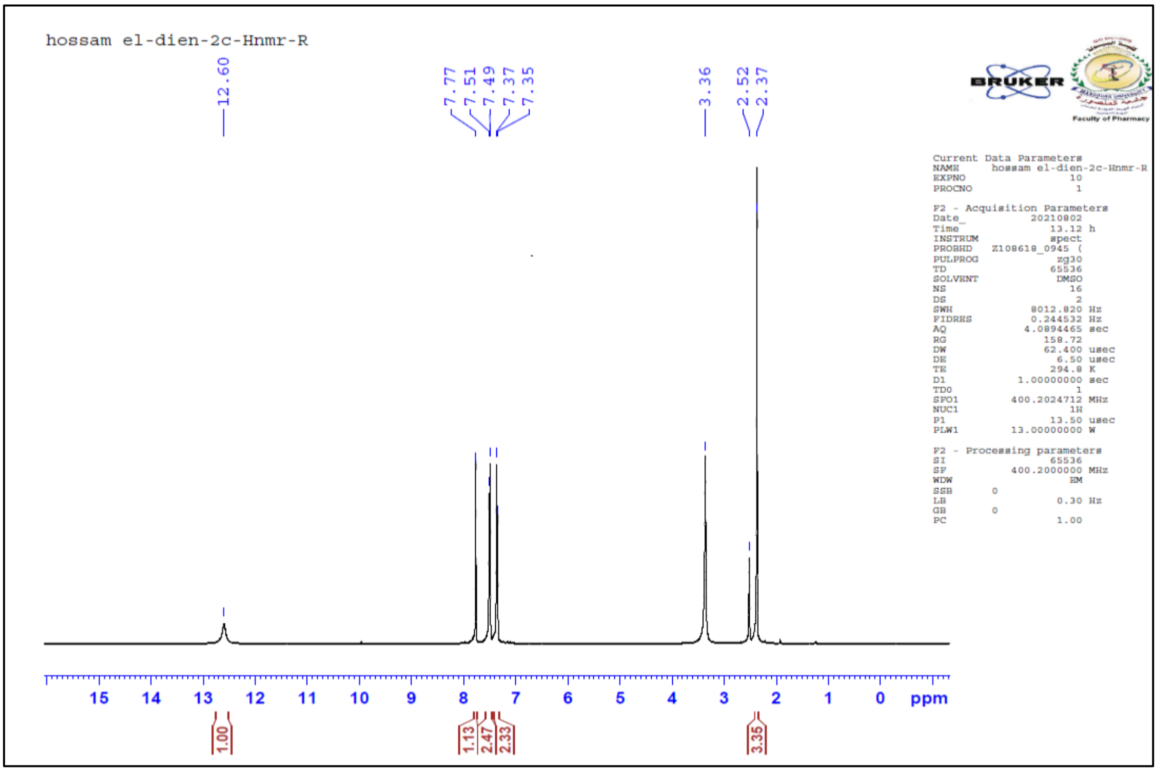

Figure S3: ^{1}HNMR spectrum of compound **1c** (400 MHz, DMSO-*d*6).

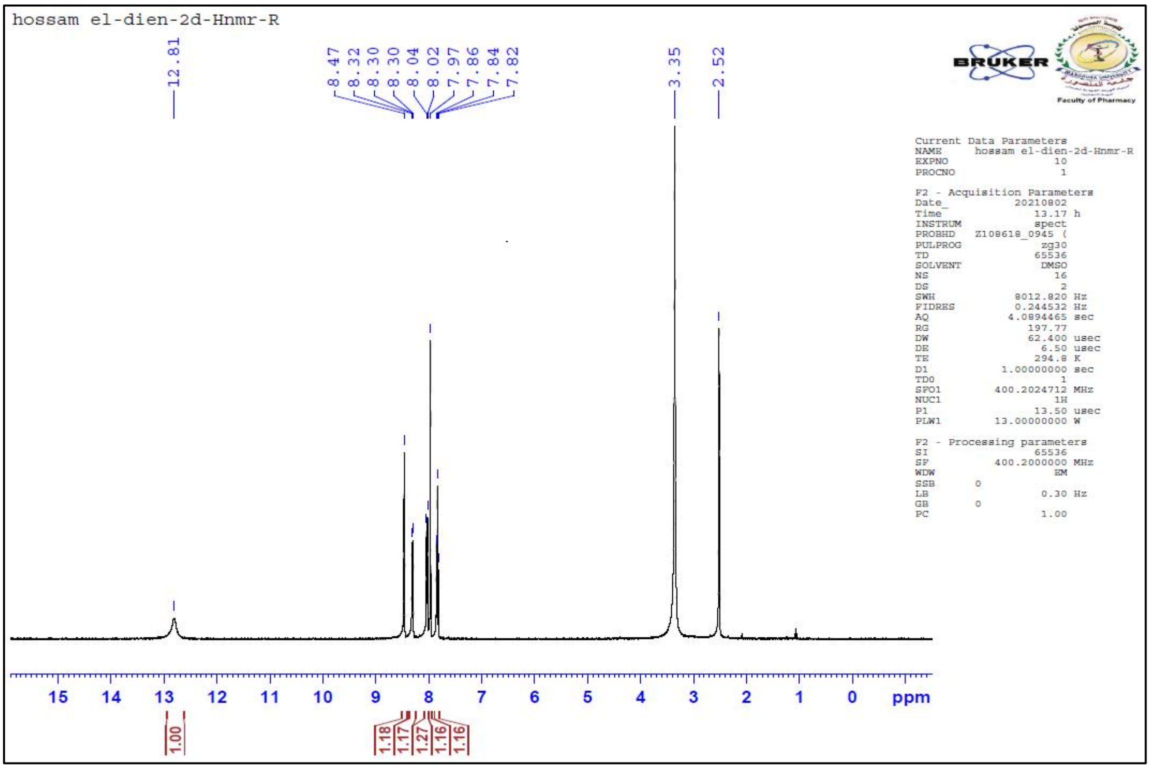

Figure S4: ^{1}HNMR spectrum of compound **1d** (400 MHz, DMSO-*d*6).

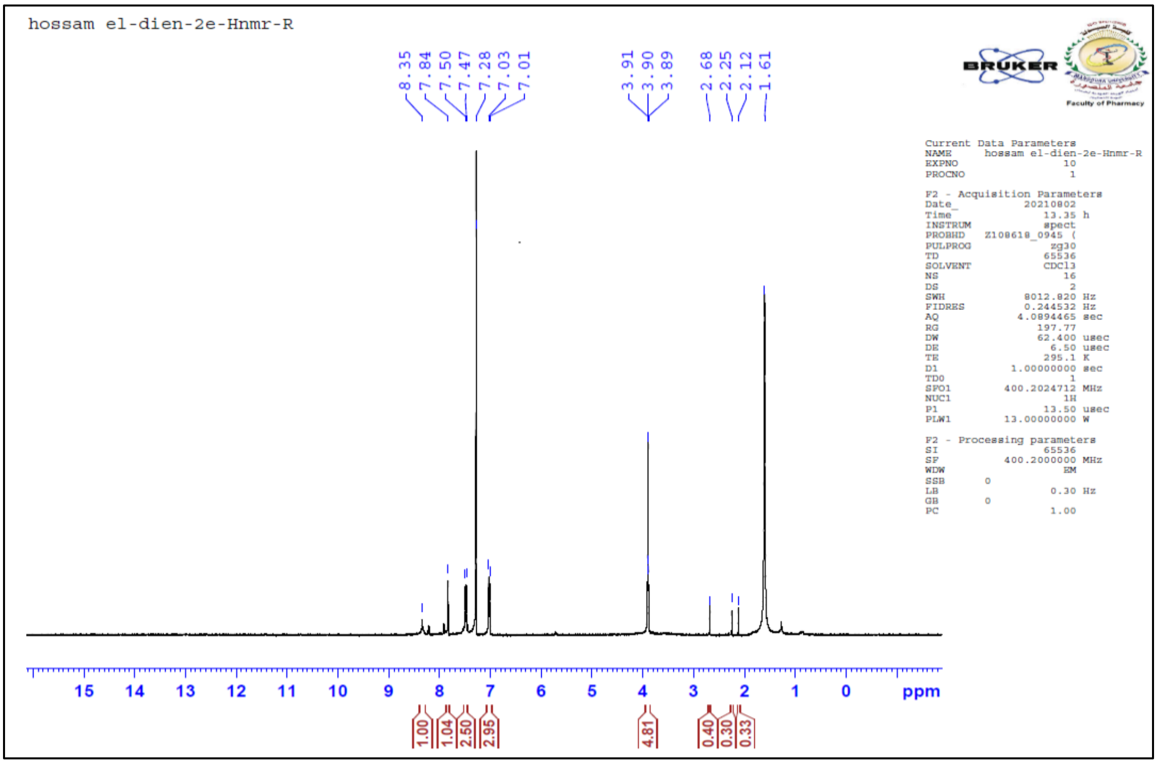

Figure S5: ¹HNMR spectrum of compound **1e** (400 MHz, $CDCl_3$).

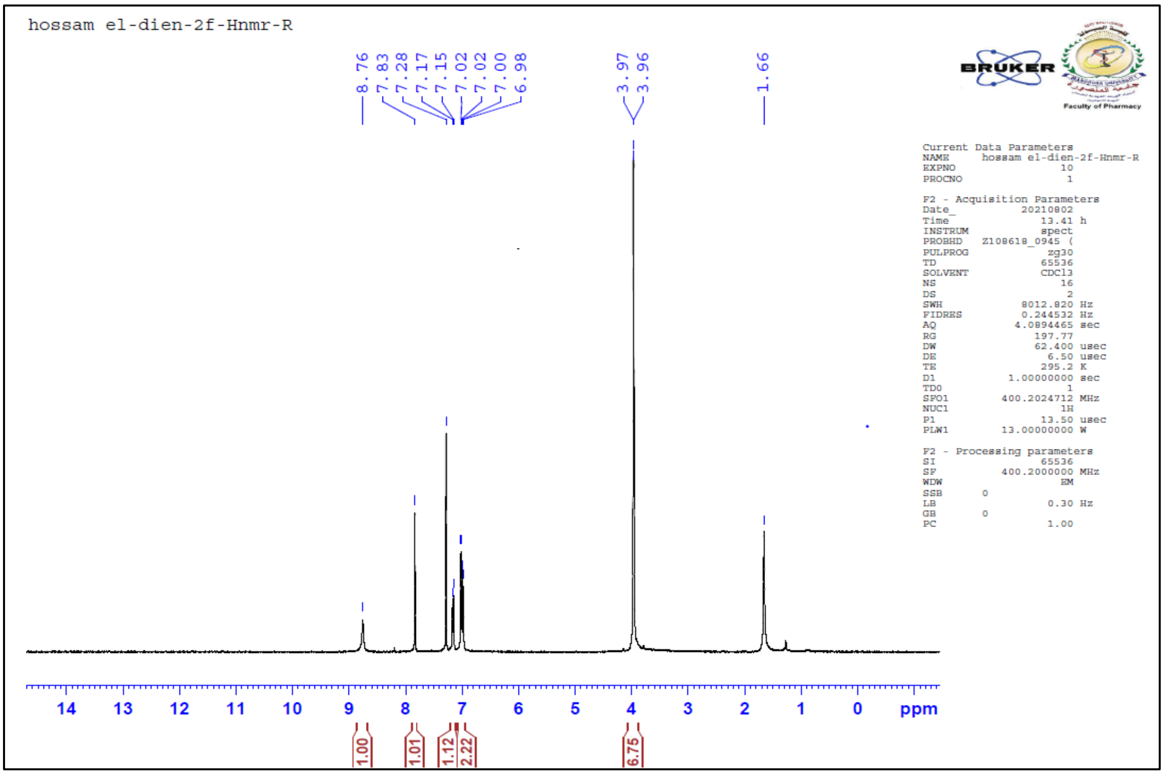

Figure S6: ¹HNMR spectrum of compound **1f** (400 MHz, $CDCl_3$).

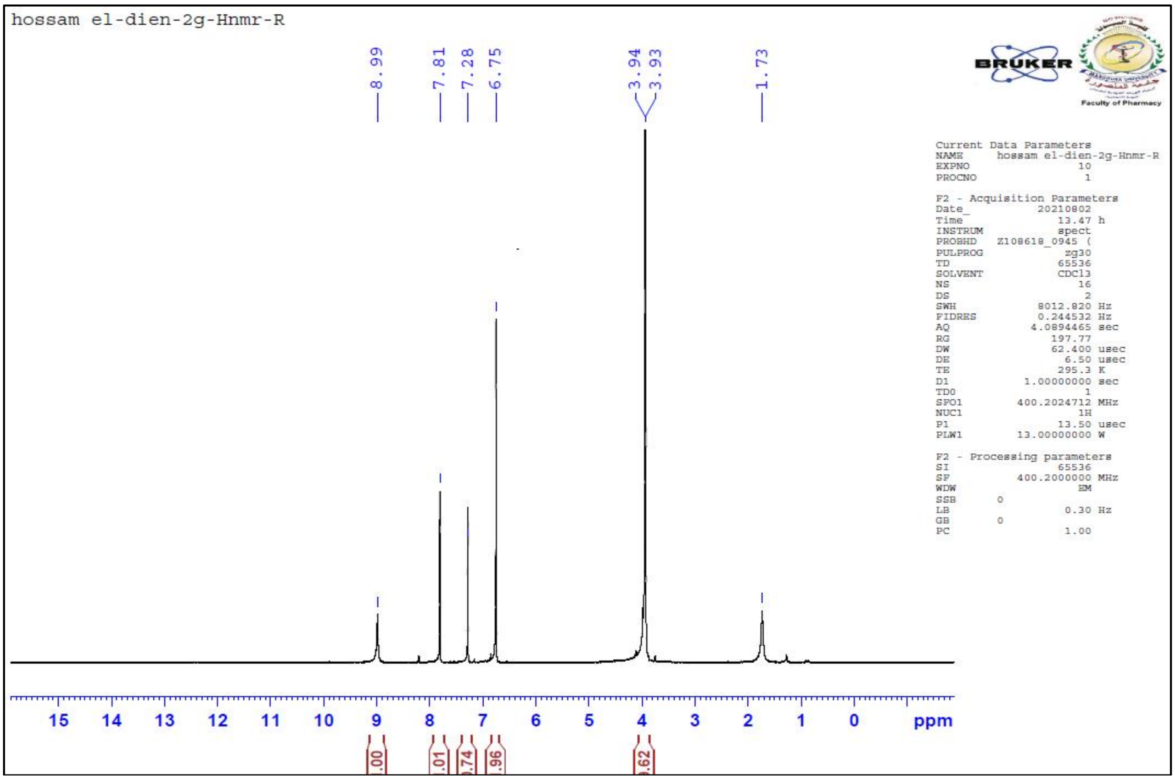

Figure S7: ^{1}HNMR spectrum of compound **1g** (400 MHz, $CDCl_3$)

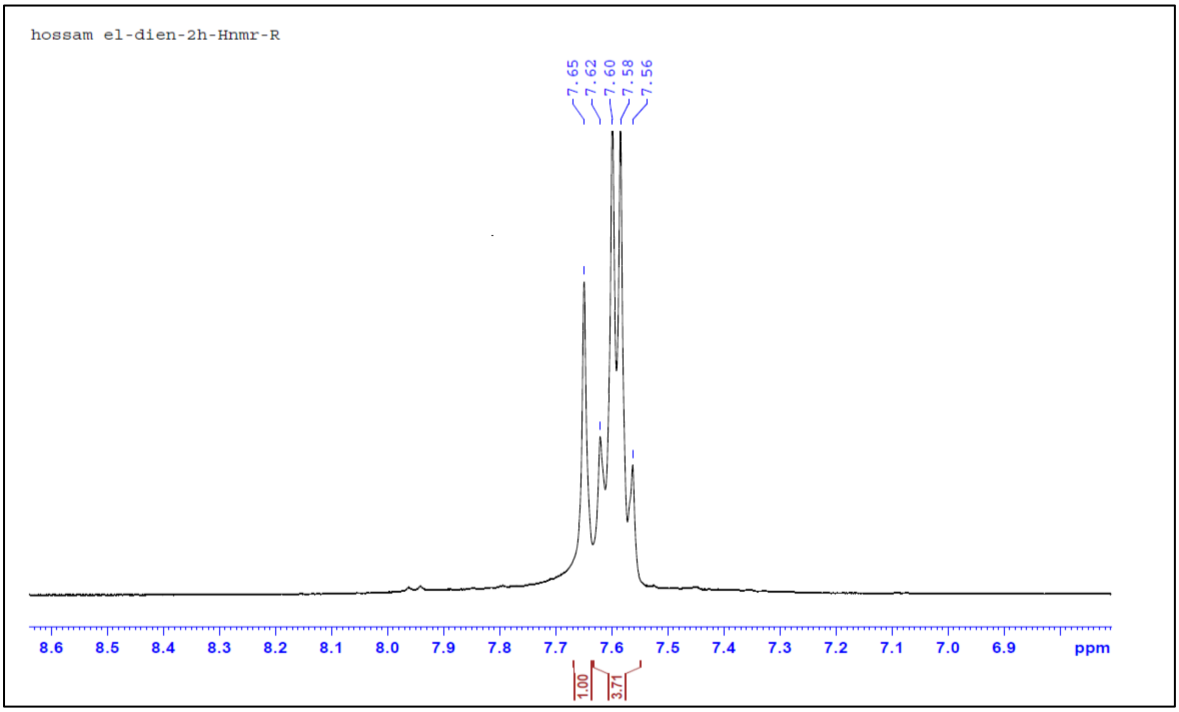

Figure S8: ^{1}HNMR spectrum of compound **1h** (400 MHz, DMSO-d_6)

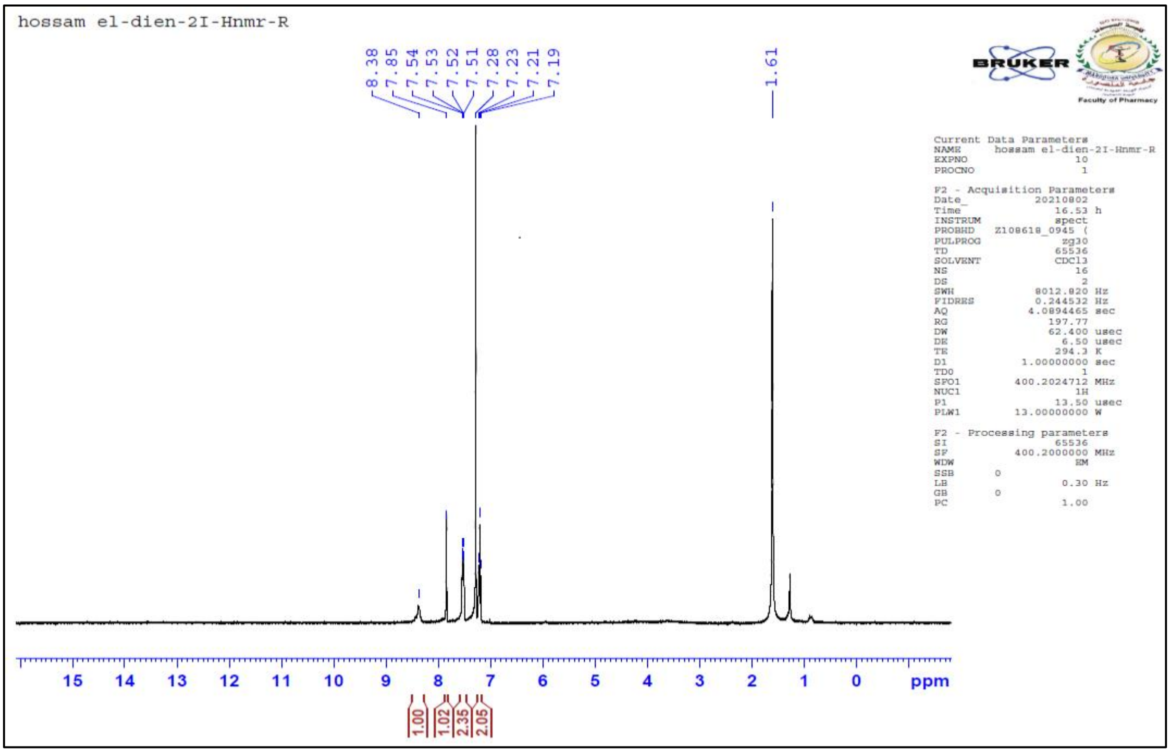

Figure S9: ^{1}HNMR spectrum of compound **1i** (400 MHz, $CDCl_3$)

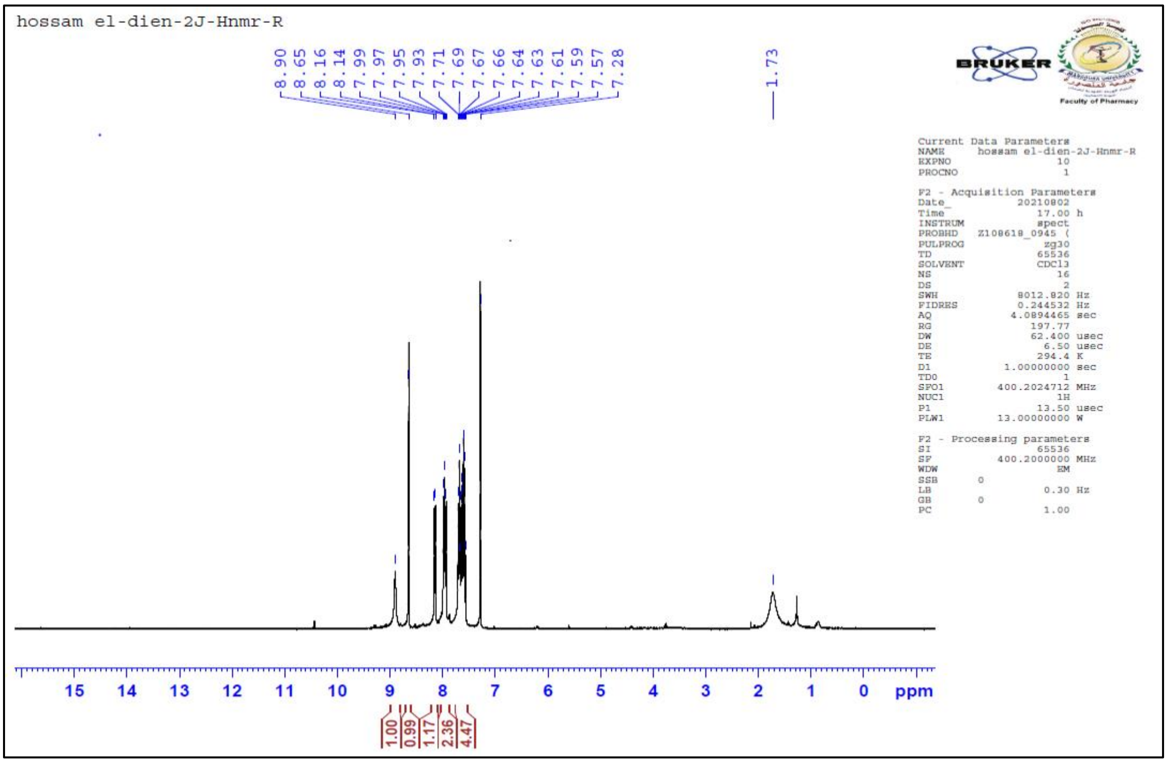

Figure S10: ^{1}HNMR spectrum of compound **1j** (400 MHz, $CDCl_3$)

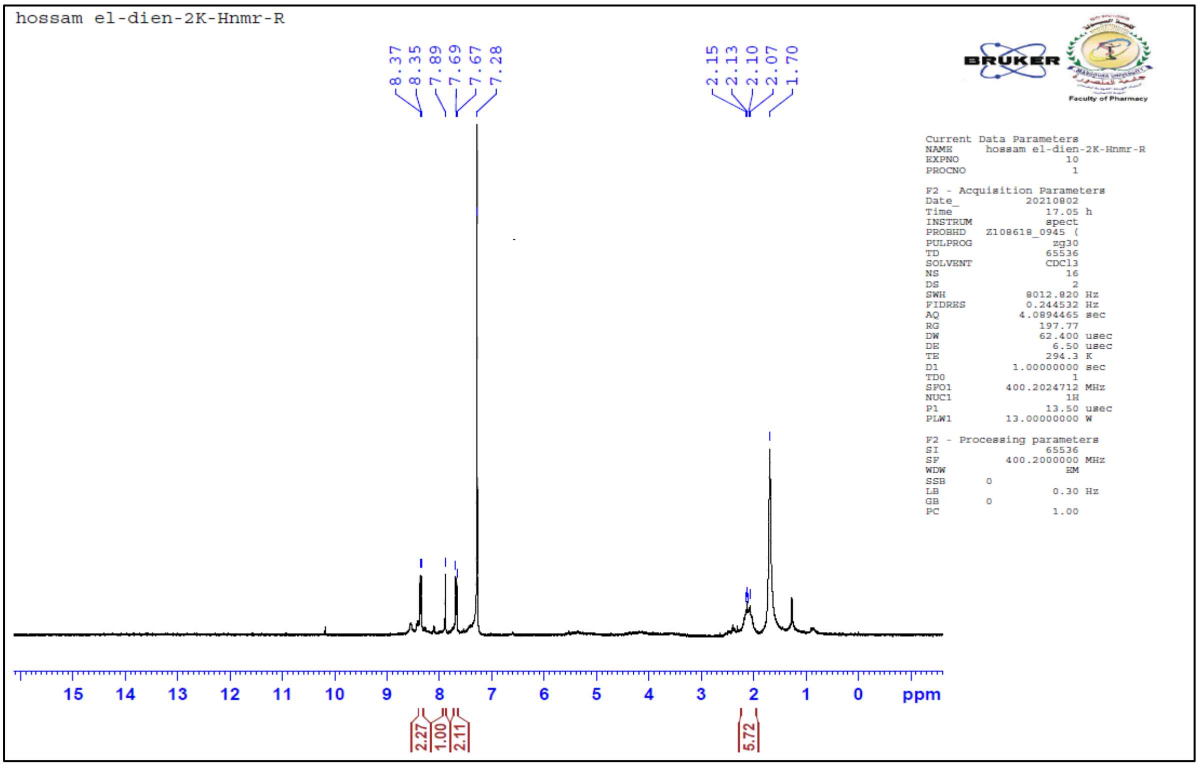

Figure S11: ^{1}HNMR spectrum of compound **1k** (400 MHz, $CDCl_3$)

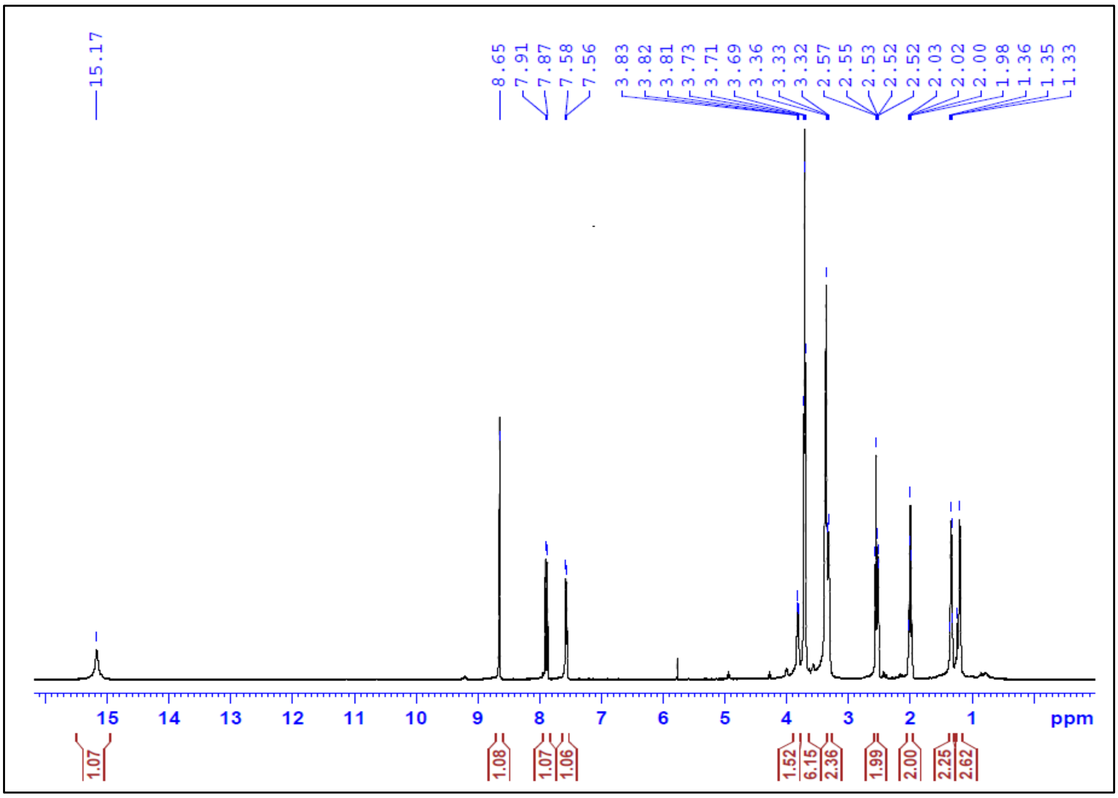

Figure S12: ^{1}HNMR spectrum of compound **2** (400 MHz, DMSO-d_6)

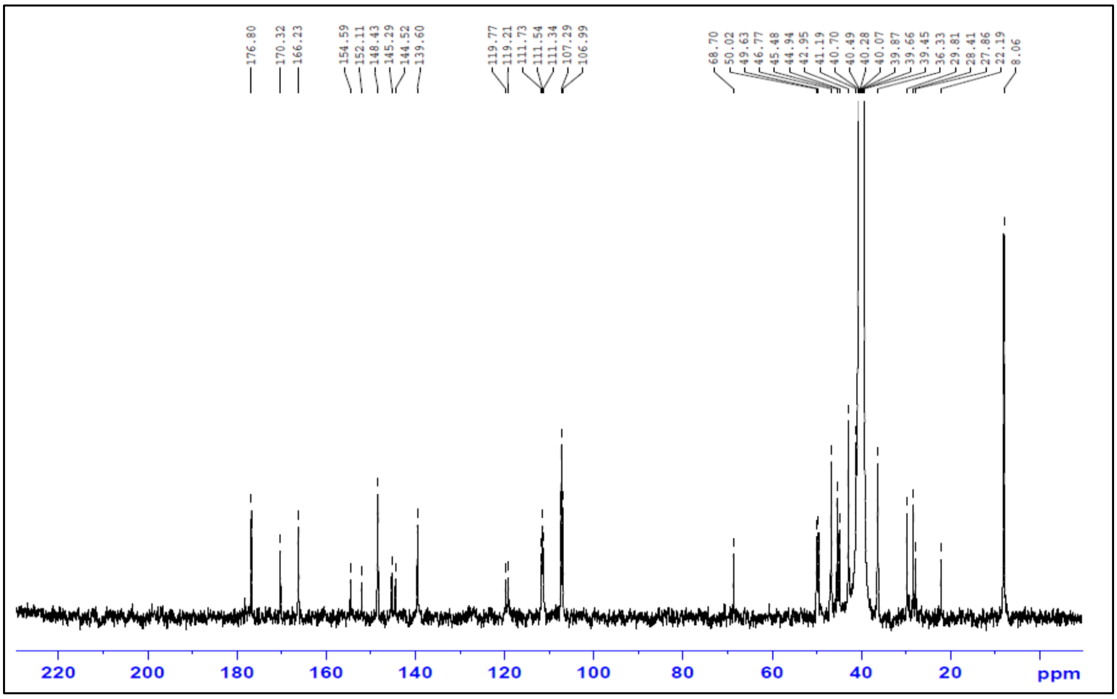

Figure S13: ^{13}CNMR spectrum of compound **2** (100 MHz, DMSO-*d*6)

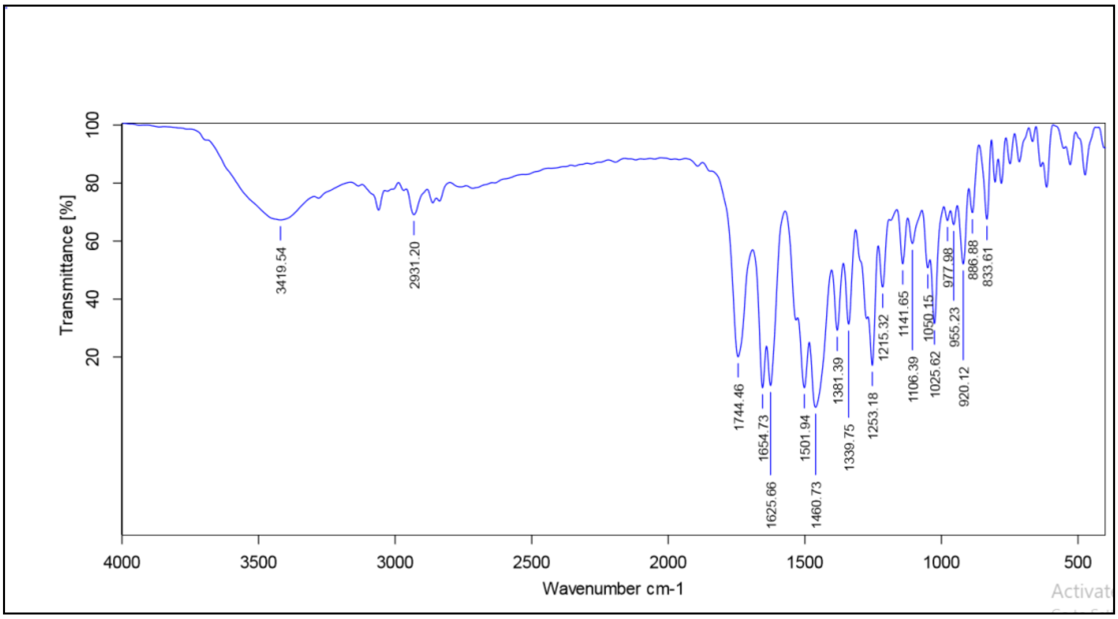

Figure S14: IR spectrum of compound **2**

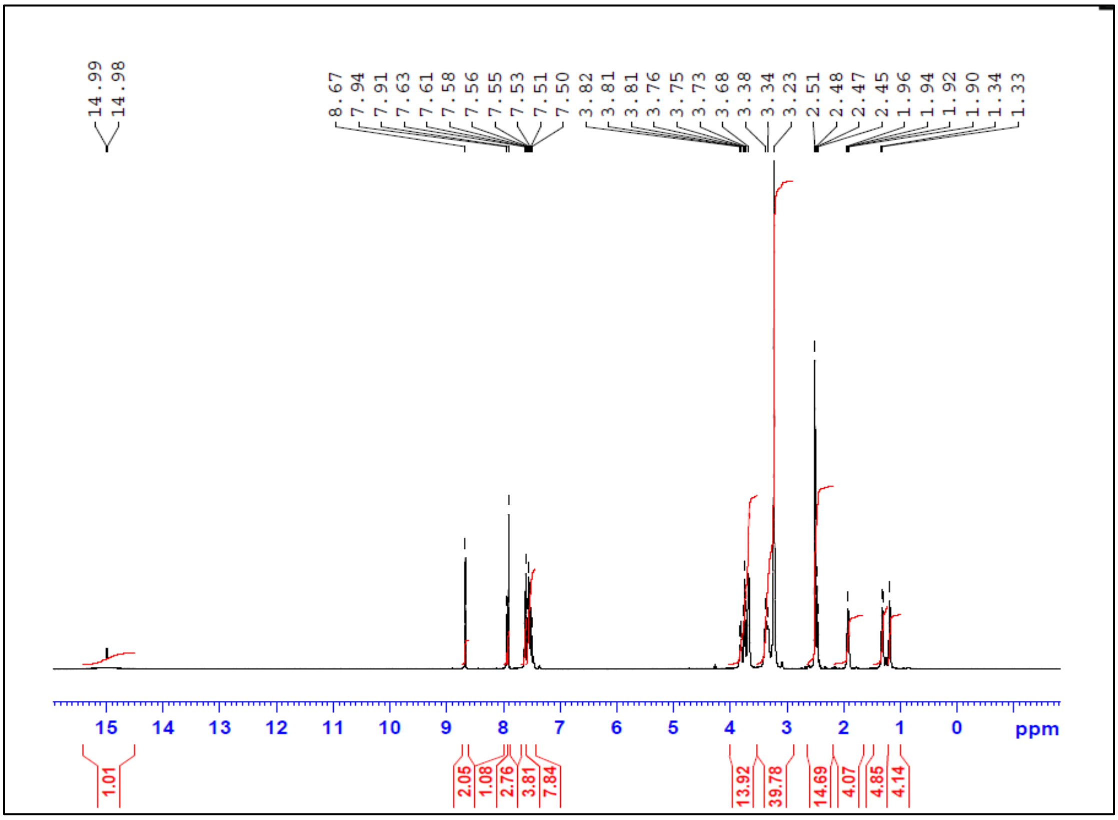

Figure S15: ^{1}HNMR spectrum of compound **3a** (400 MHz, DMSO-d_6)

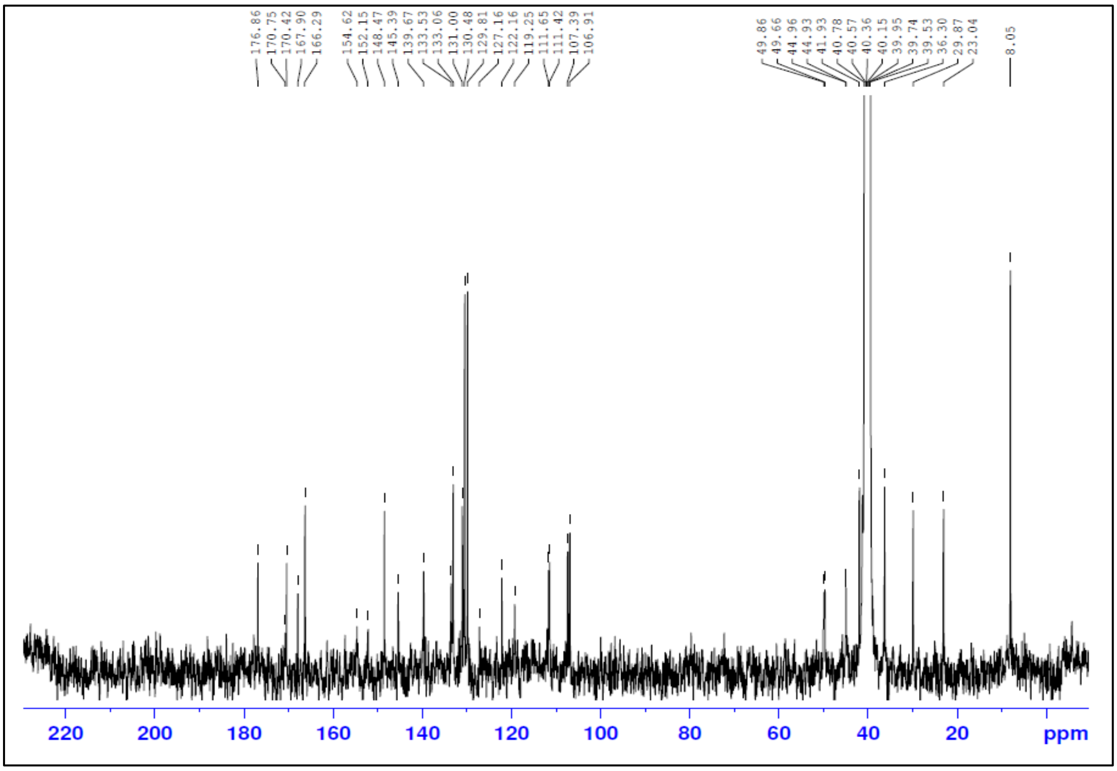

Figure S16: ^{13}CNMR spectrum of compound 3a (100 MHz, DMSO-d_6)

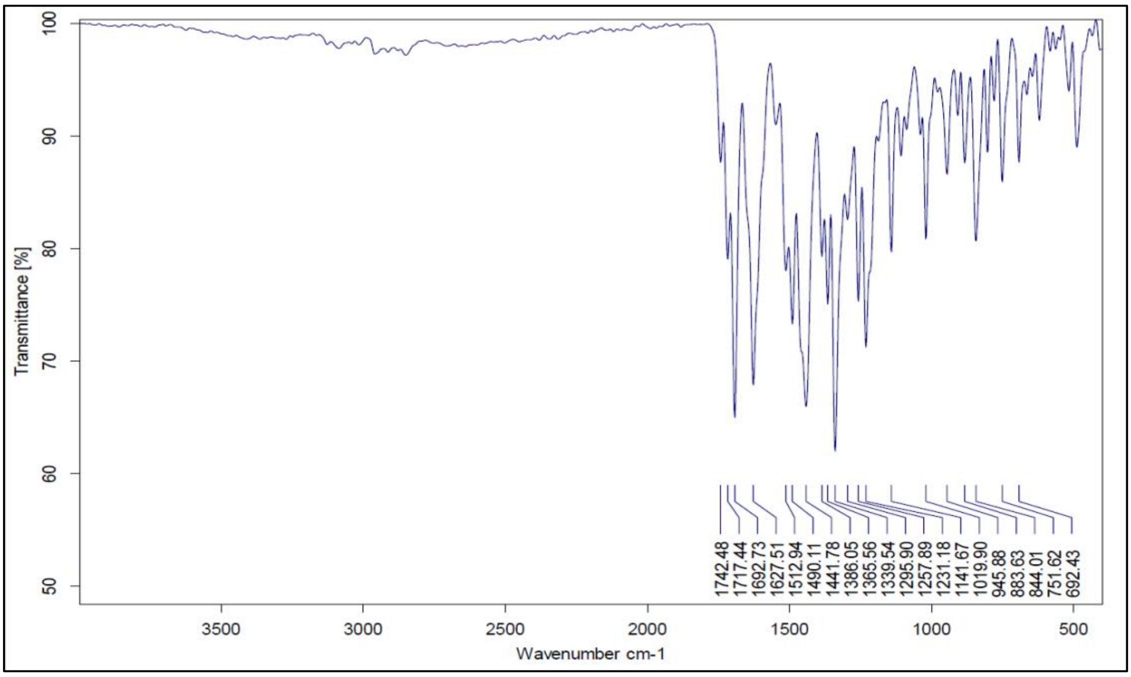

Figure S17: IR spectrum of compound **3a.**

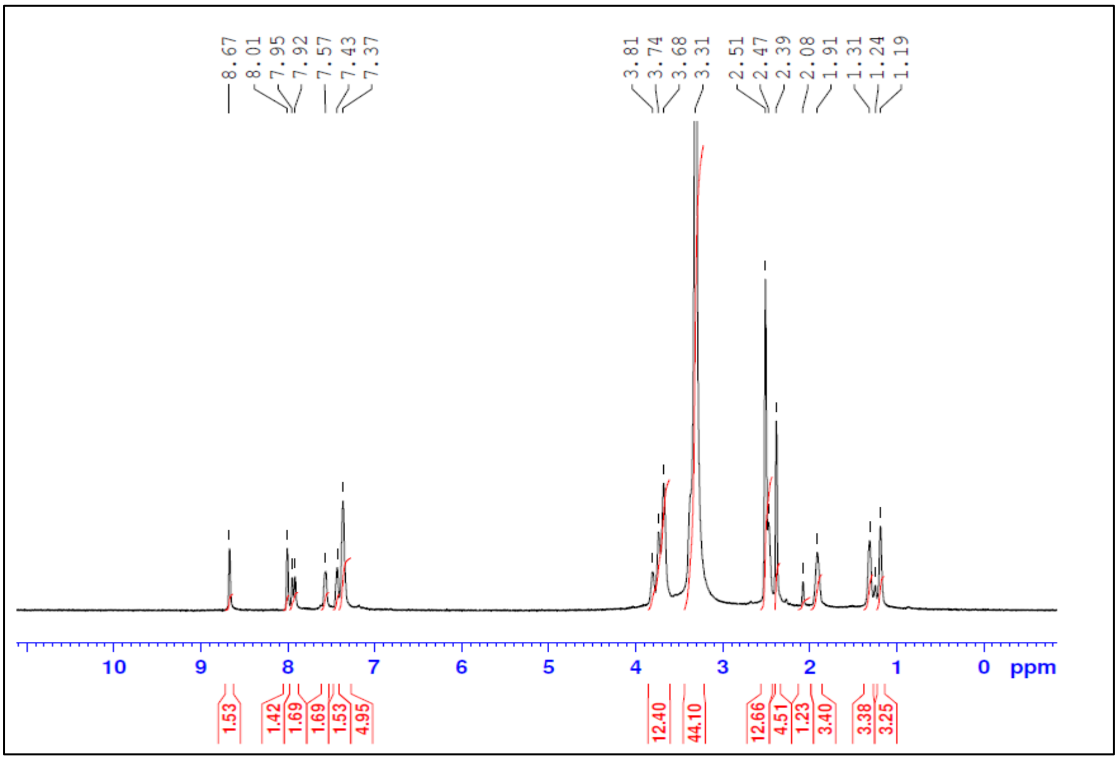

Figure S18: ^{1}HNMR spectrum of compound **3b** (400 MHz, DMSO-d_6).

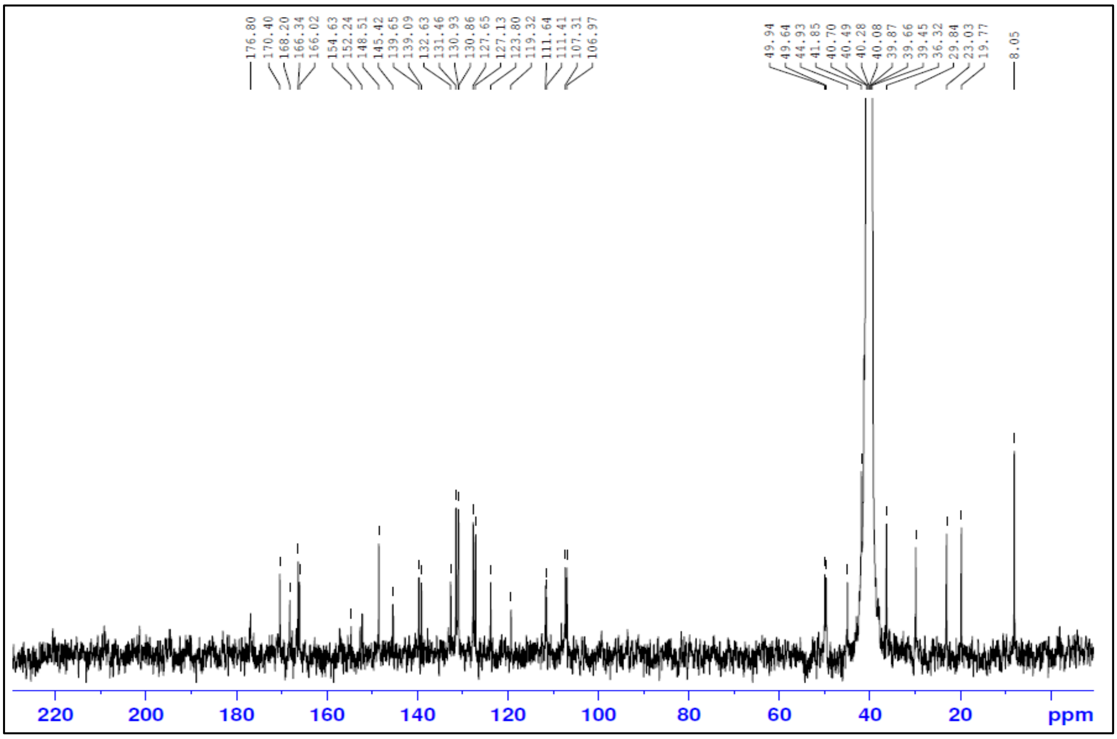

Figure S19: ^{13}CNMR spectrum of compound **3b** (100 MHz, DMSO-d_6).

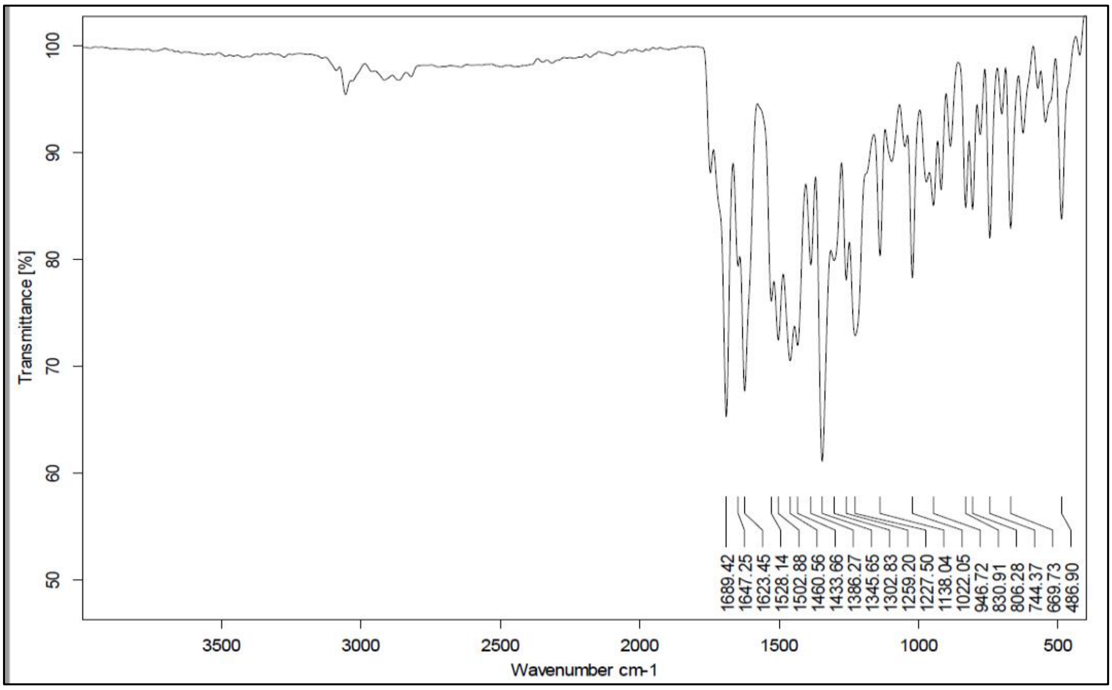

Figure S20: IR spectrum of compound **3b.**

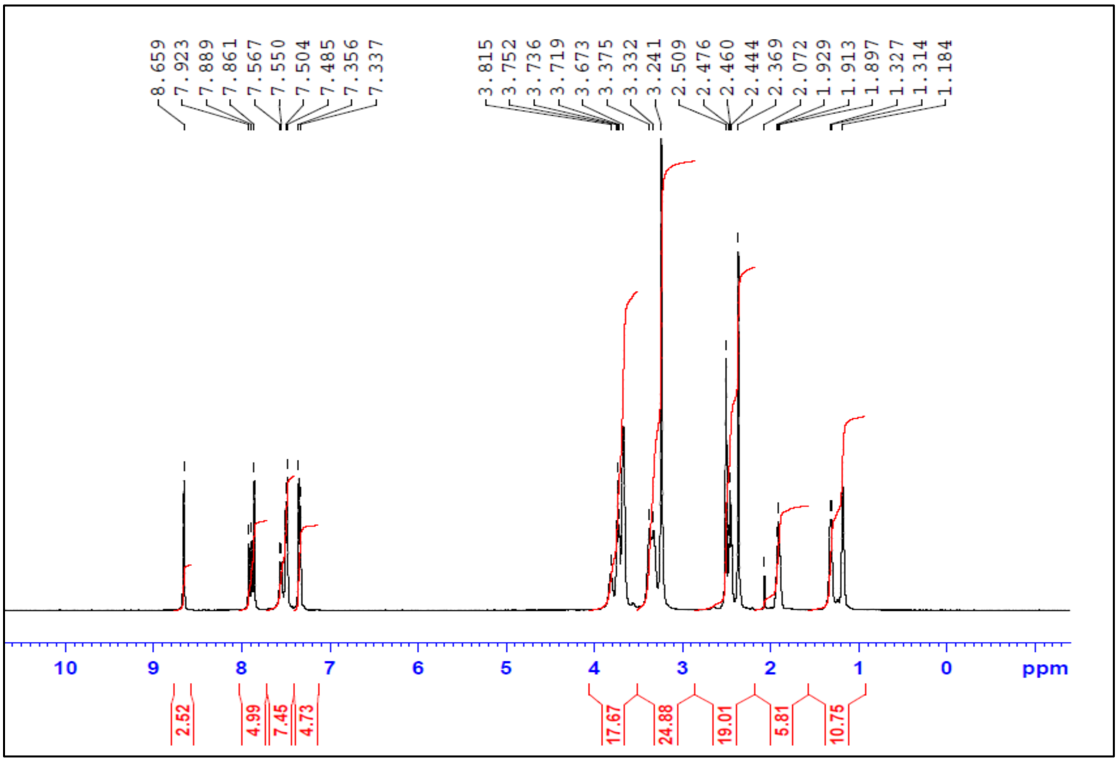

Figure S21: ^{1}HNMR spectrum of compound **3c** (400 MHz, DMSO-*d*6)

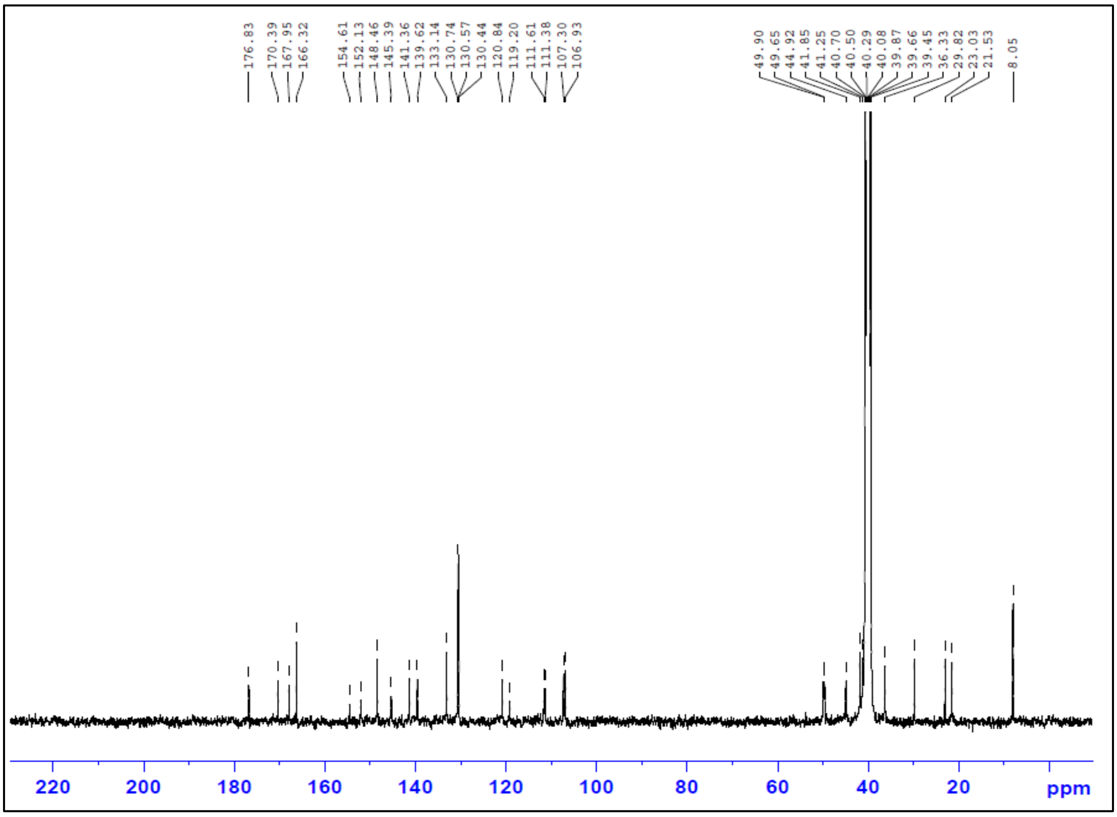

Figure S22: ^{13}CNMR spectrum of compound **3c** (100 MHz, DMSO-*d*6)

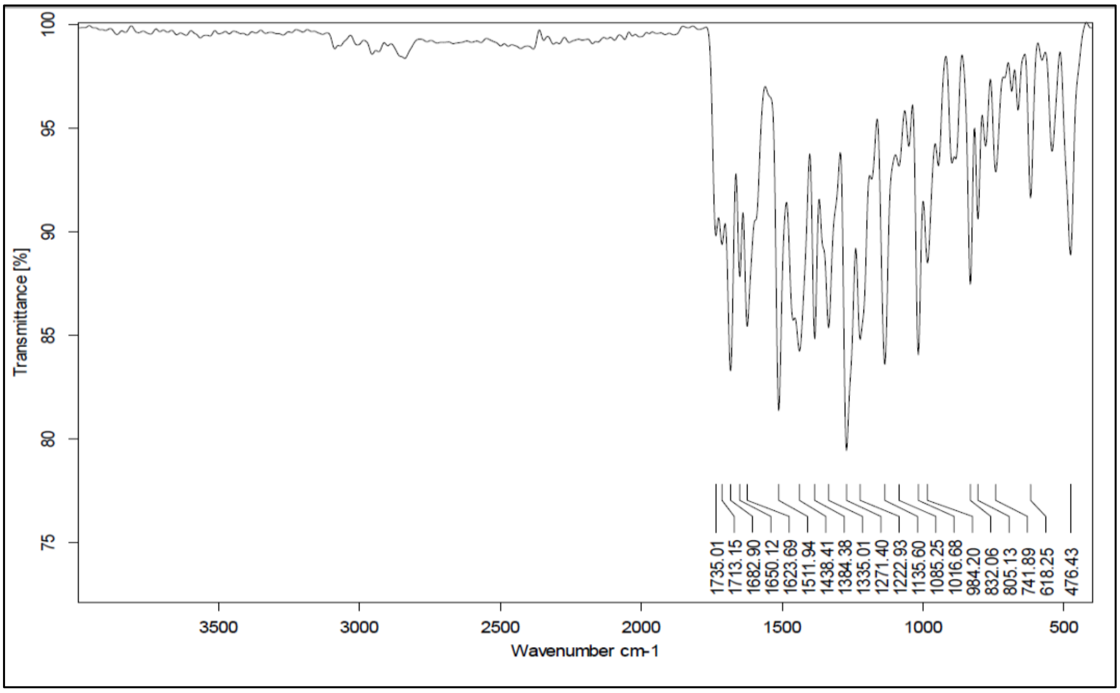

Figure S23: IR spectrum of compound **3c.**

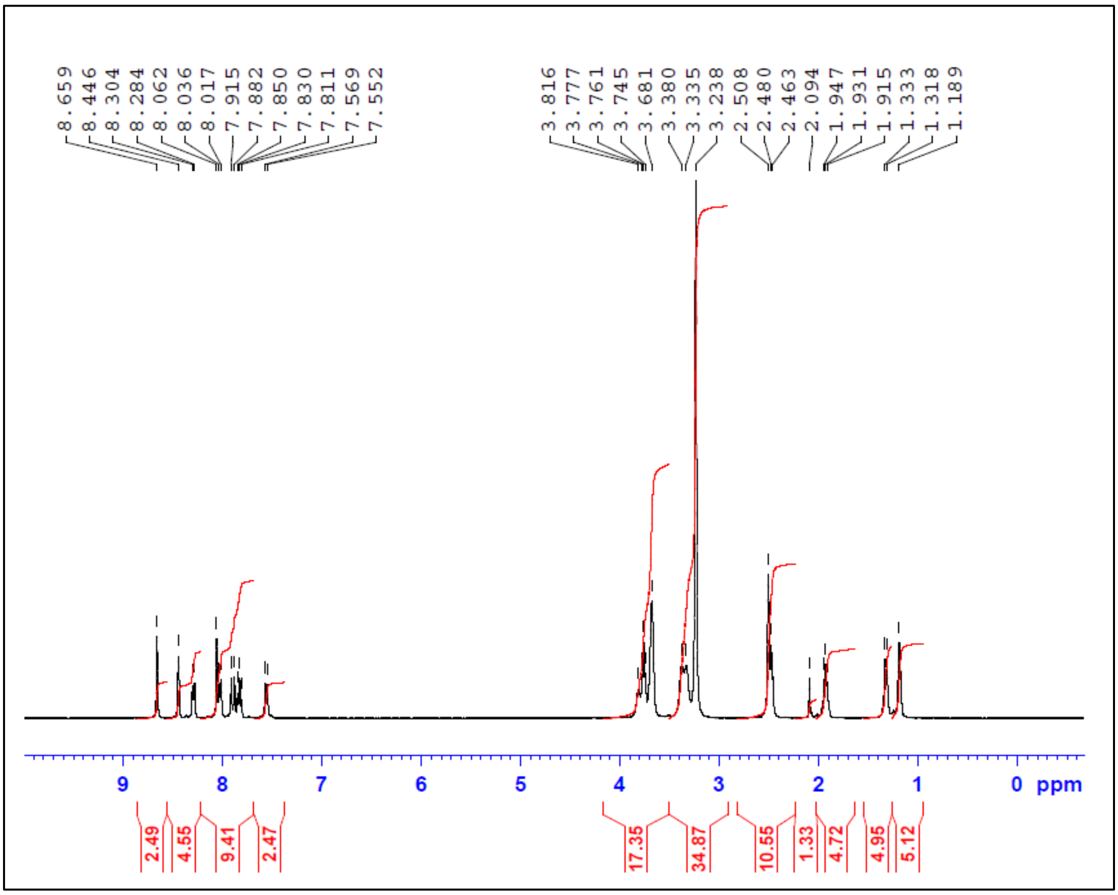

Figure S24: ^{1}HNMR spectrum of compound **3d** (400 MHz, DMSO-d_6).

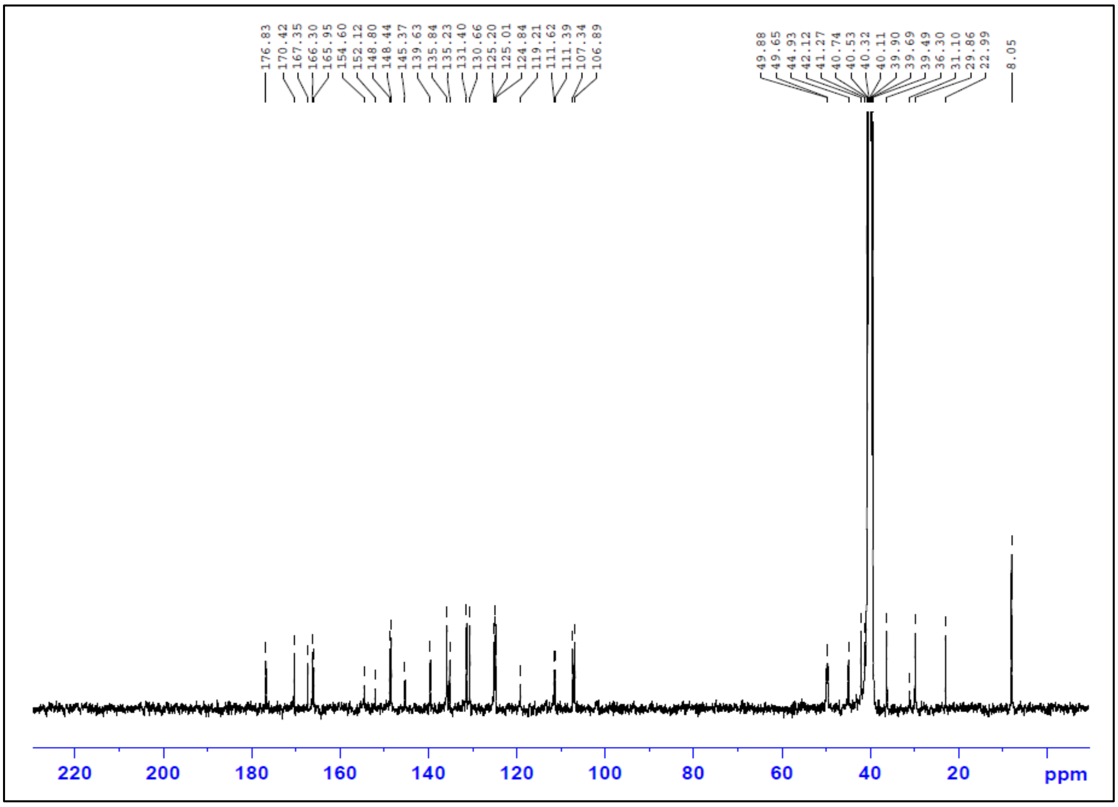

Figure S25: ^{13}CNMR spectrum of compound **3d** (100 MHz, DMSO-d_6)

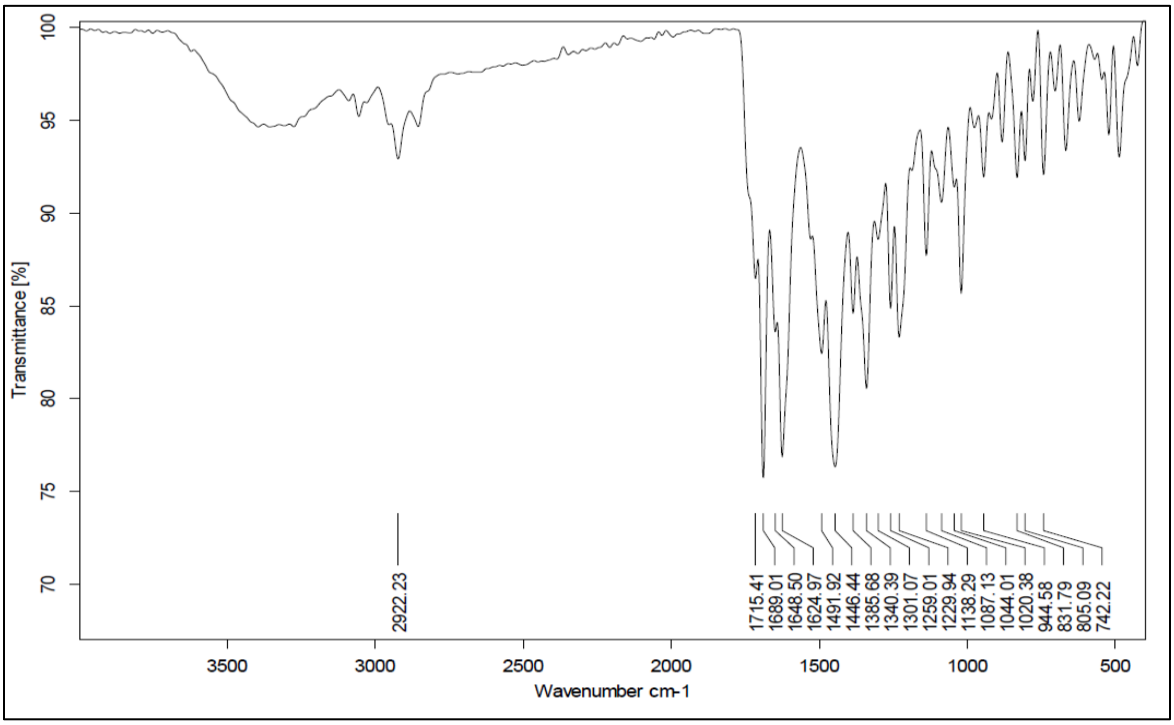

Figure S26: IR spectrum of compound **3d.**

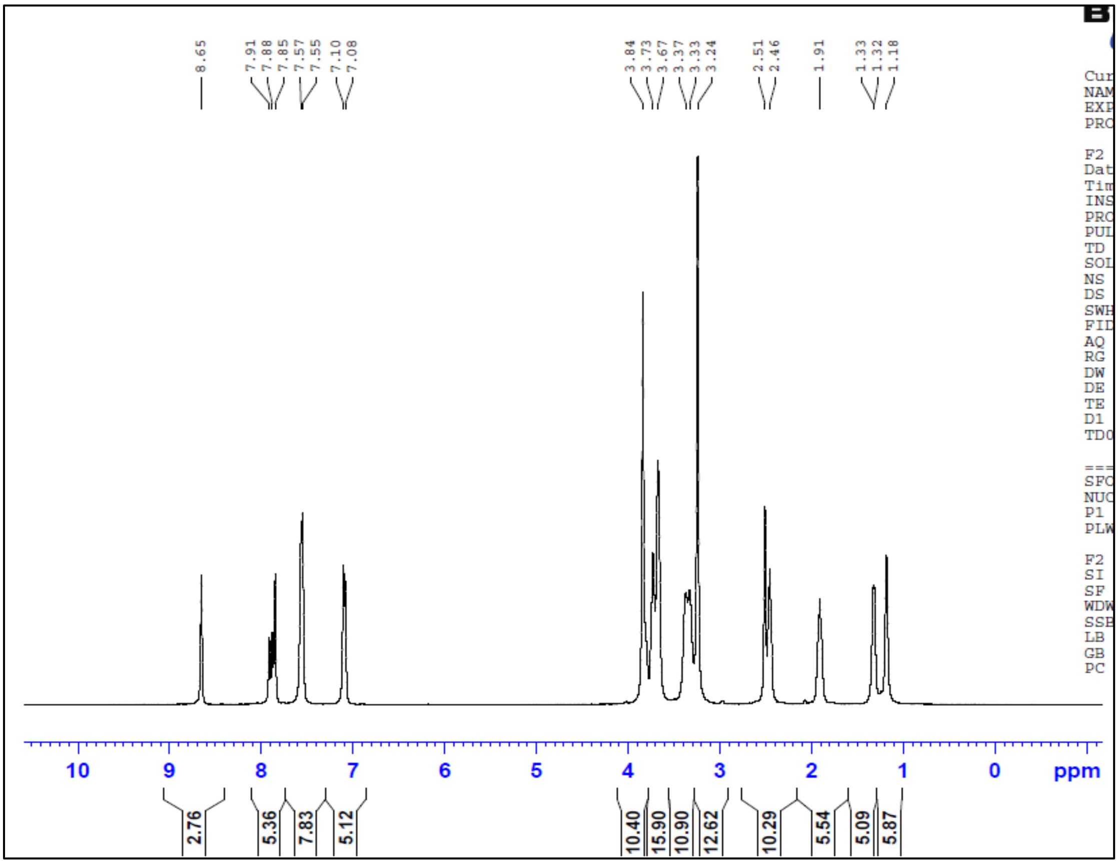

Figure S27: ^{1}HNMR spectrum of compound **3e** (400 MHz, DMSO-d_6).

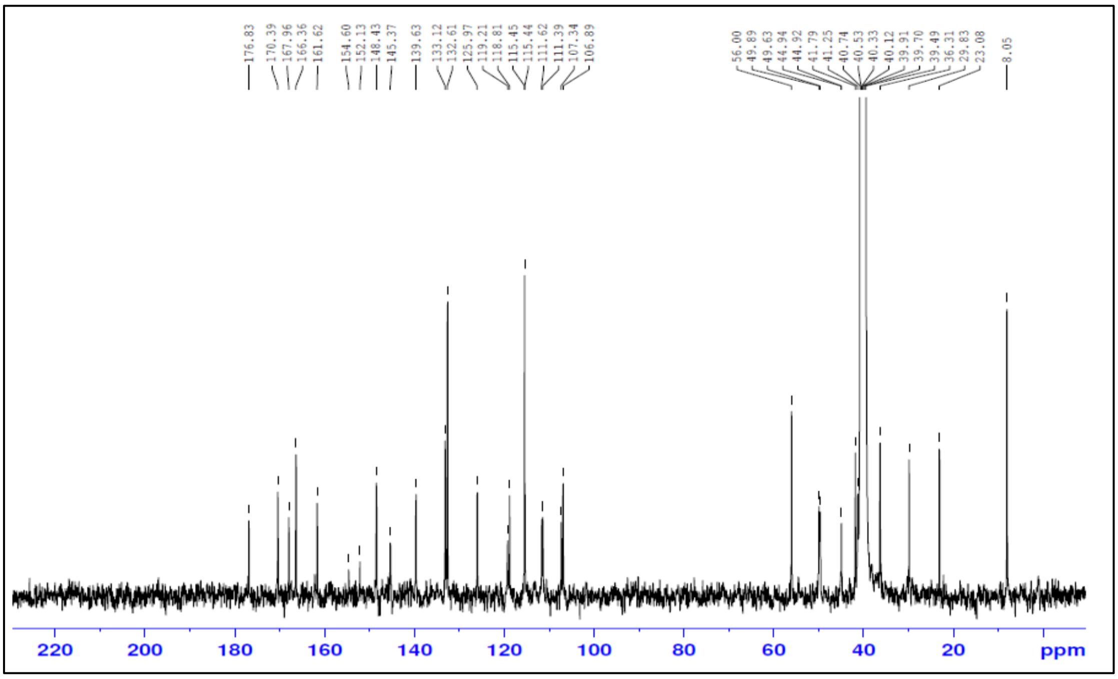

Figure S28: ^{13}CNMR spectrum of compound **3e** (100 MHz, DMSO-d_6).

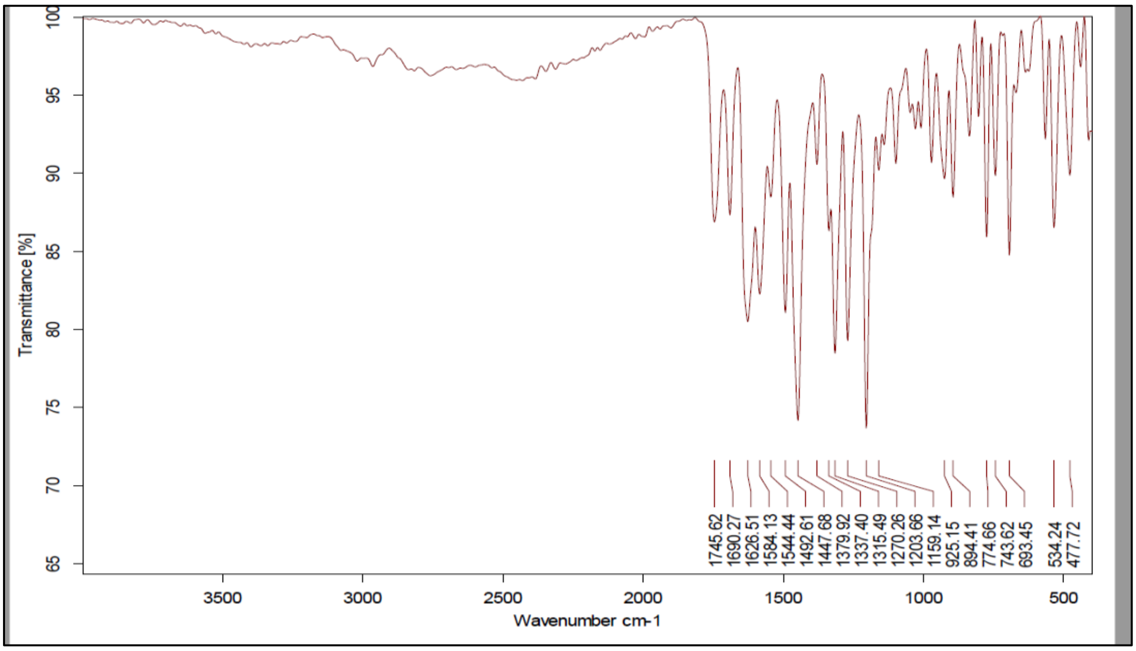

Figure S29: IR spectrum of compound **3e.**

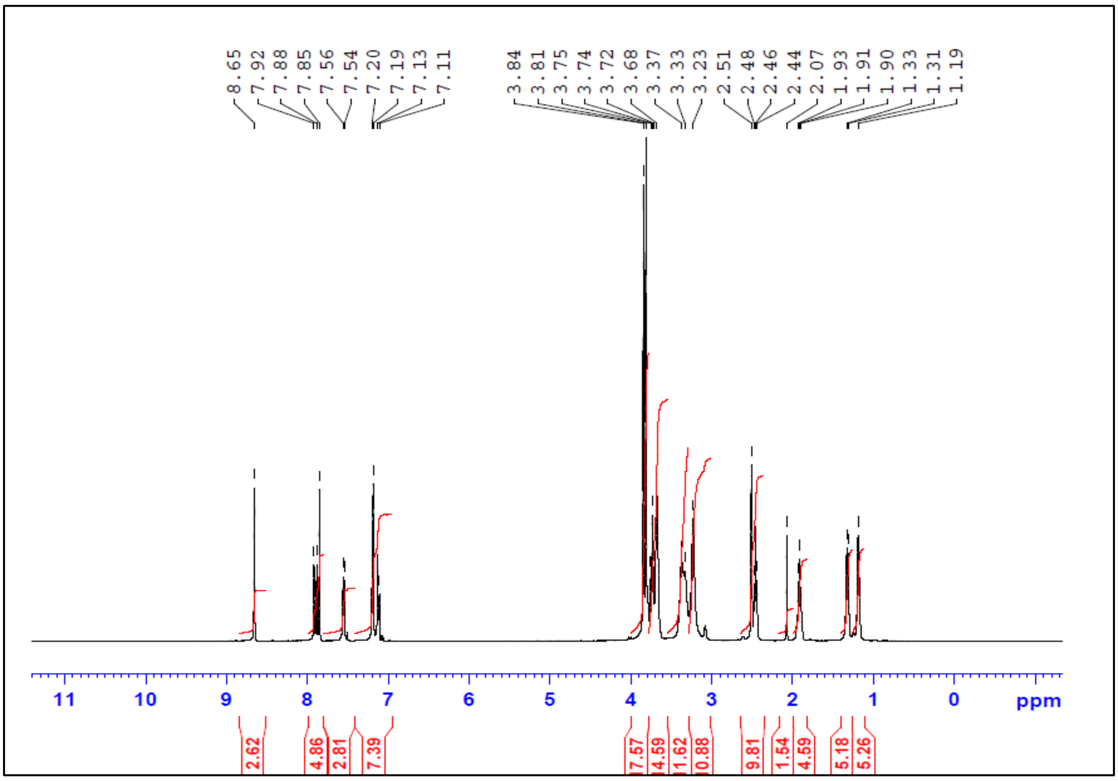

Figure S30: ^{1}HNMR spectrum of compound **3f** (400 MHz, DMSO-d_6).

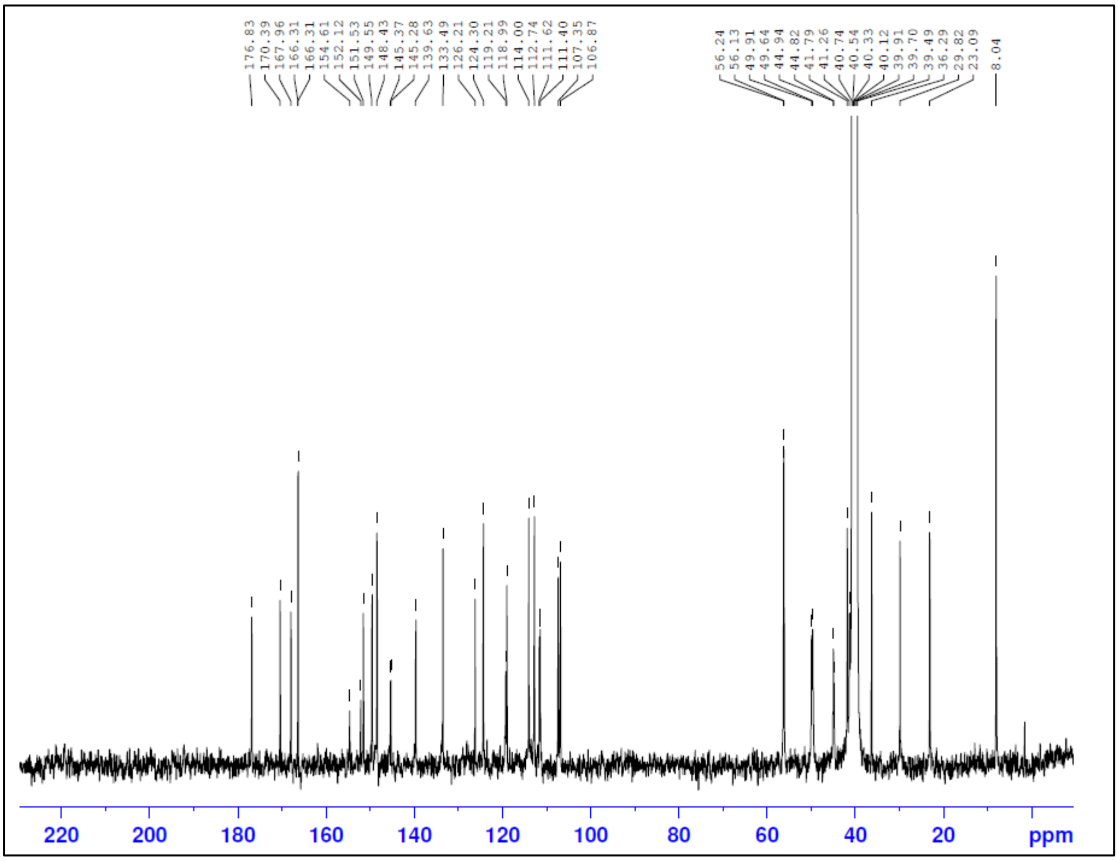

Figure S31: ^{13}CNMR spectrum of compound **3f** (100 MHz, DMSO-*d*6).

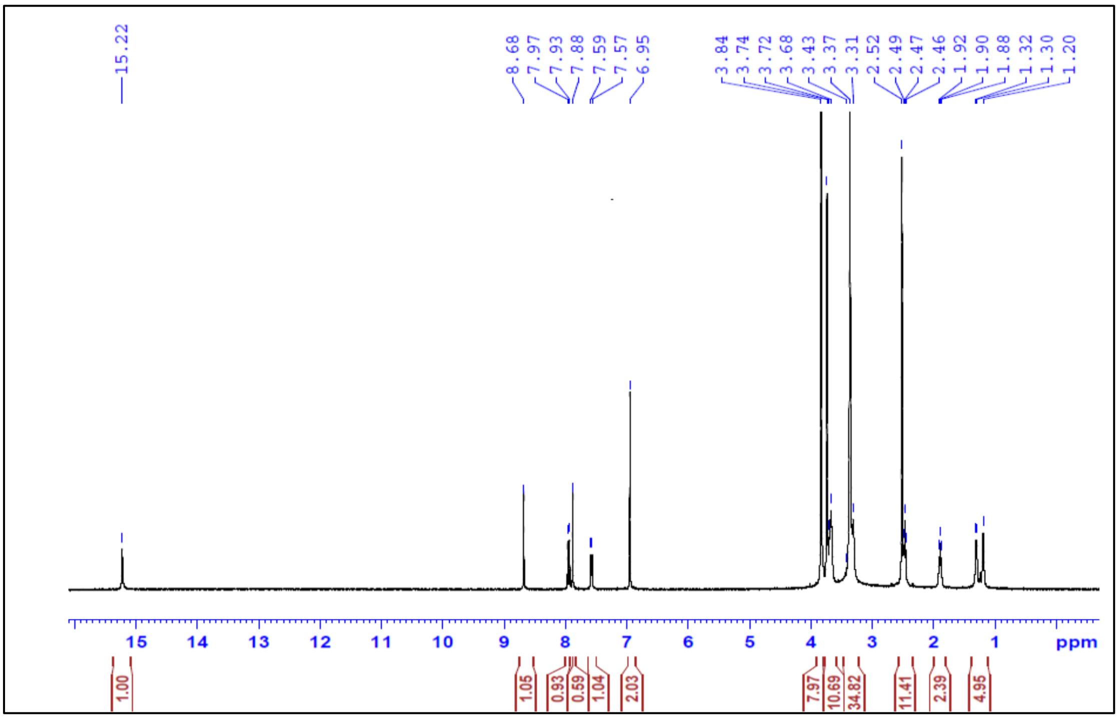

Figure S32: ^{1}HNMR spectrum of compound **3g** (400 MHz, DMSO-*d*6).

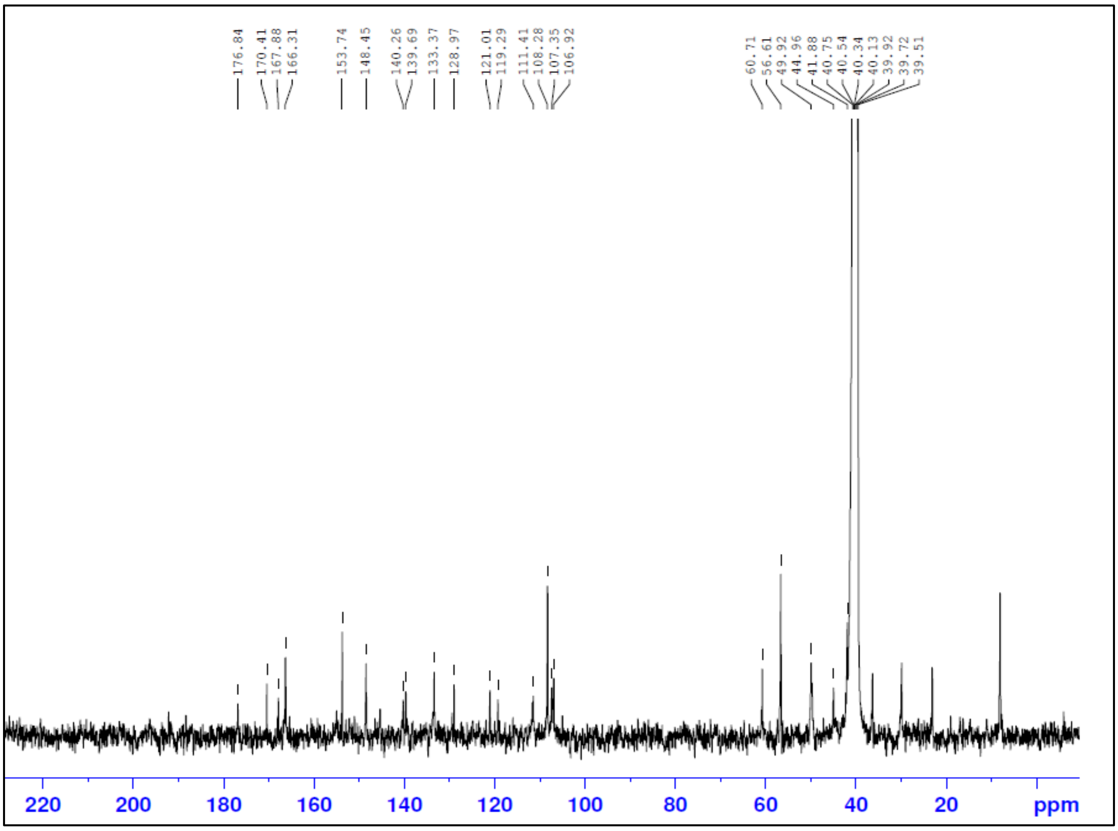

Figure S33: ^{13}CNMR spectrum of compound **3g** (100 MHz, DMSO-d_6).

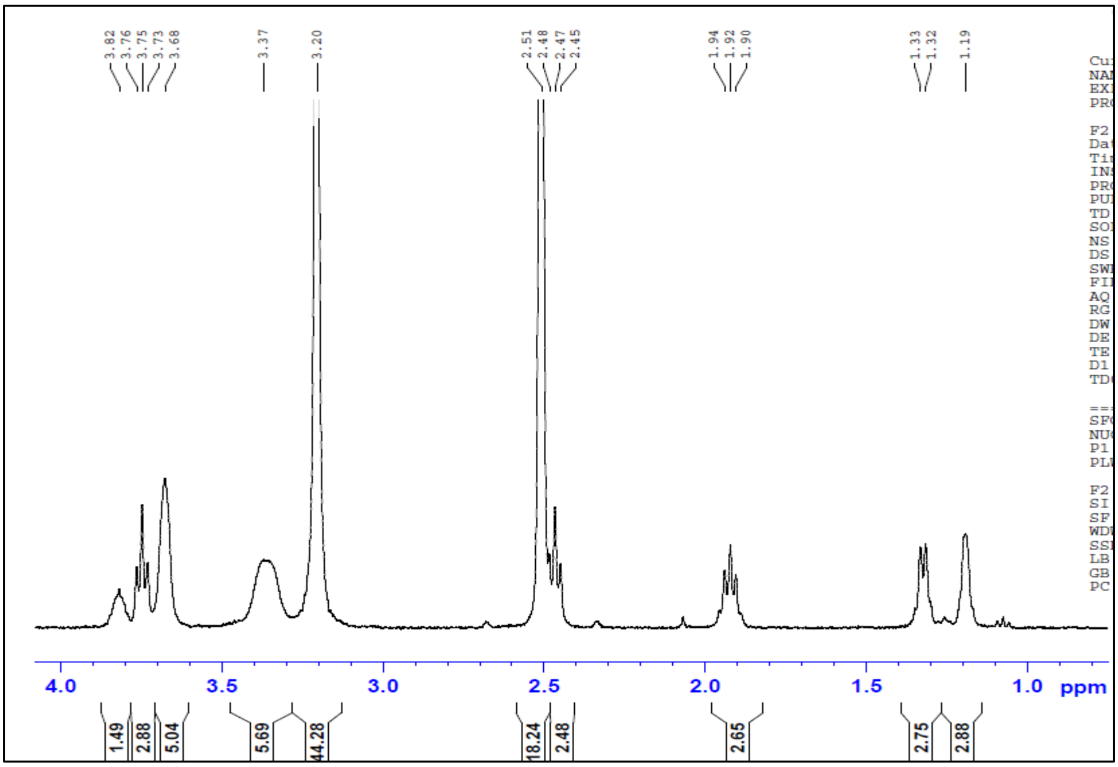

Figure S34: ^{1}HNMR spectrum of compound **3h** (400 MHz, DMSO-d_6).

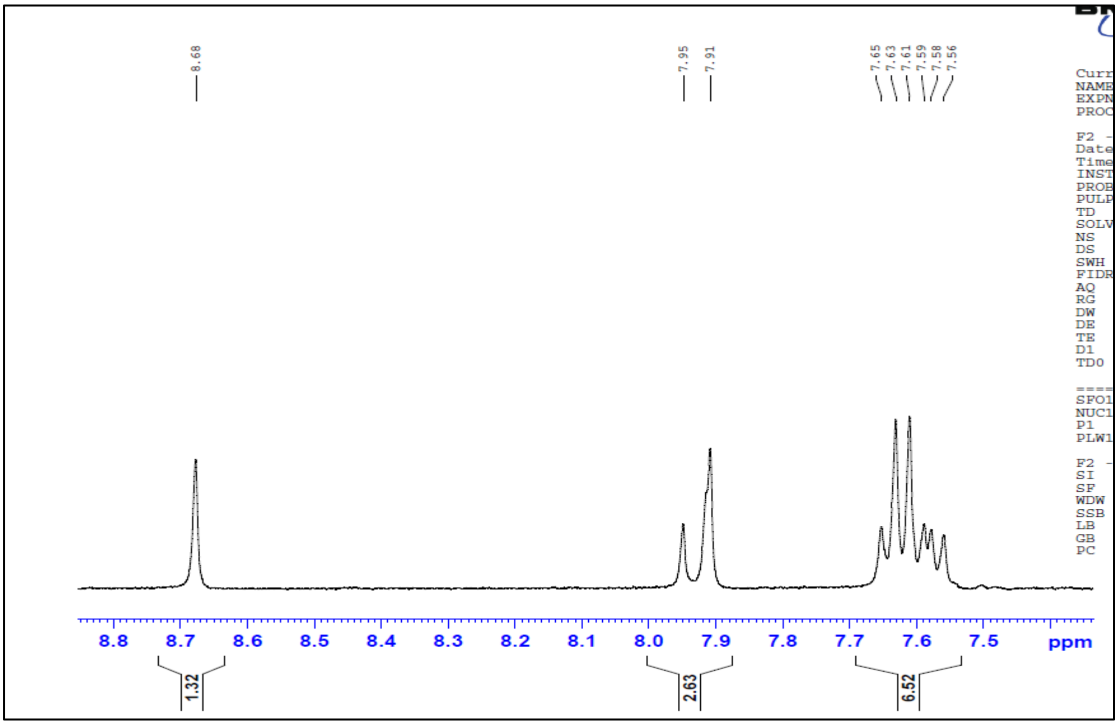

Figure S35: ^{1}HNMR spectrum of compound **3h aromatic region** (400 MHz, DMSO-*d*6).

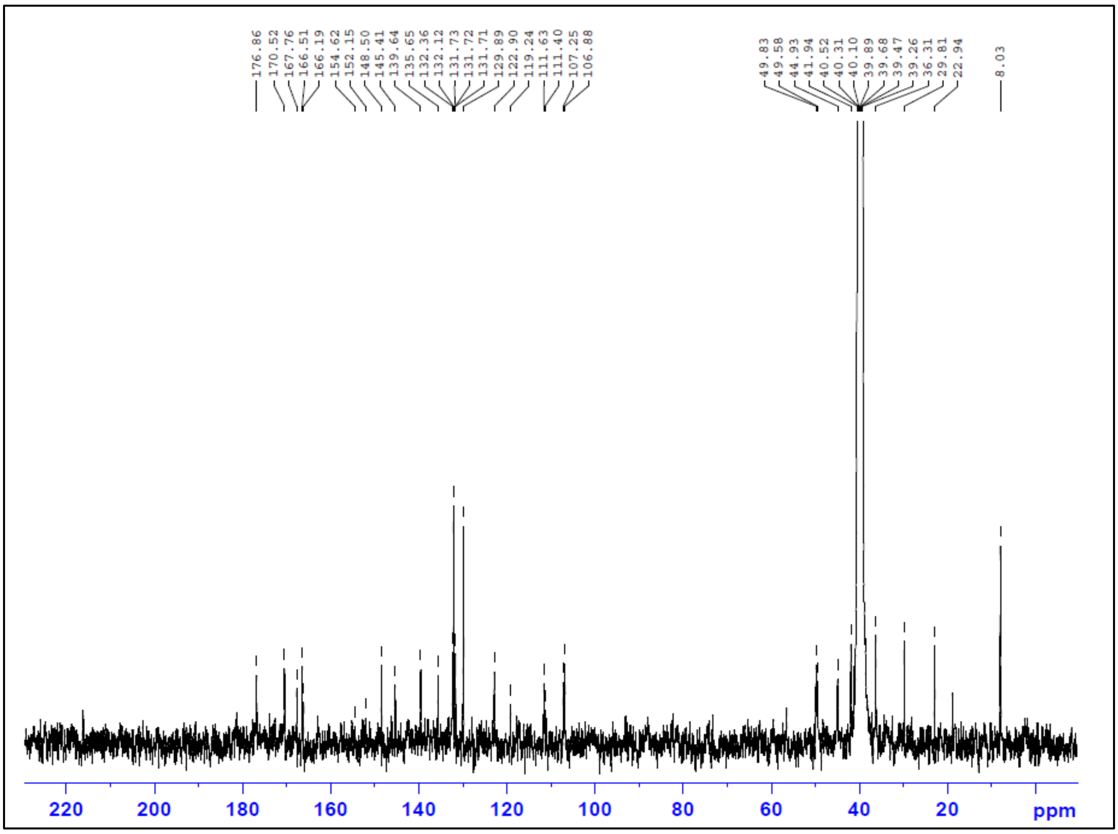

Figure S36: ^{13}CNMR spectrum of compound **3h** (100 MHz, DMSO-*d*6).

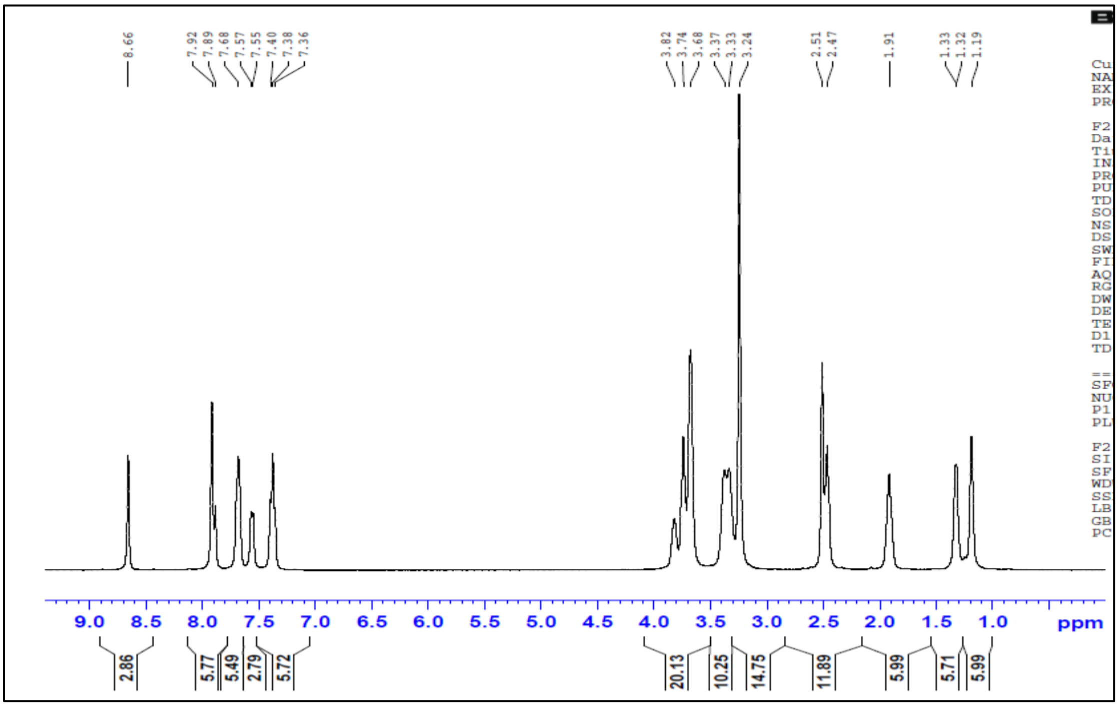

Figure S37: ^{1}HNMR spectrum of compound **3i** (400 MHz, DMSO-*d*6).

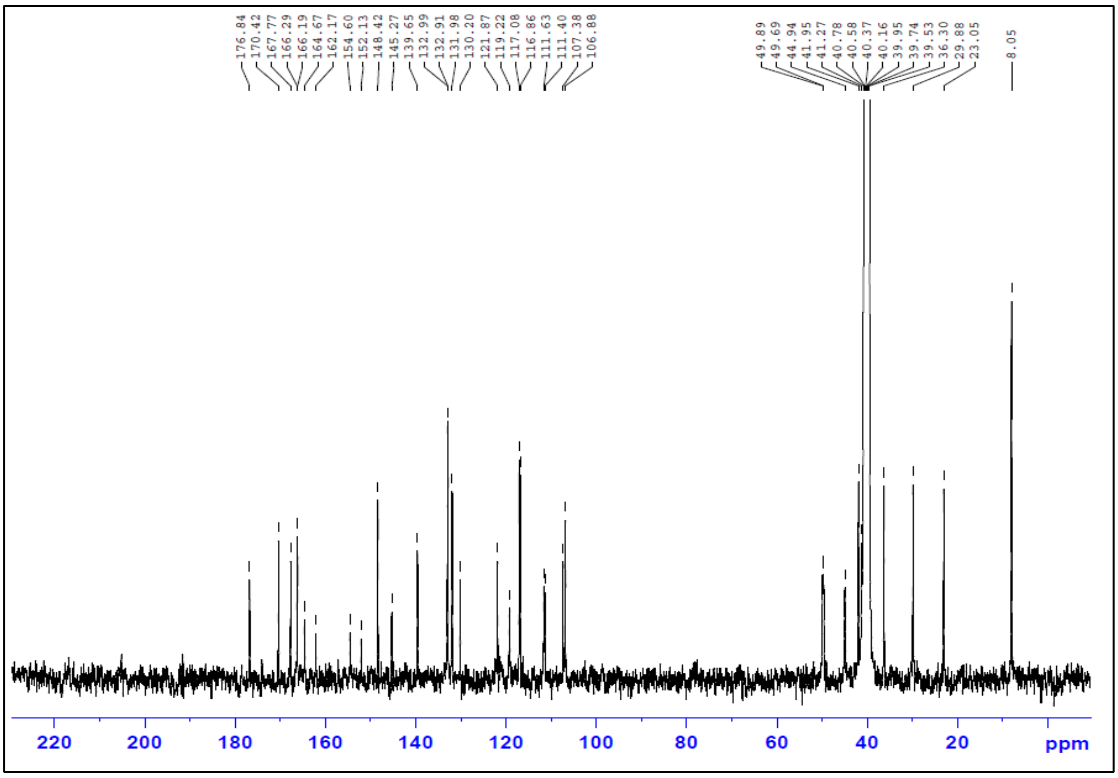

Figure S38: ^{13}CNMR spectrum of compound **3i** (100 MHz, DMSO-*d*6).

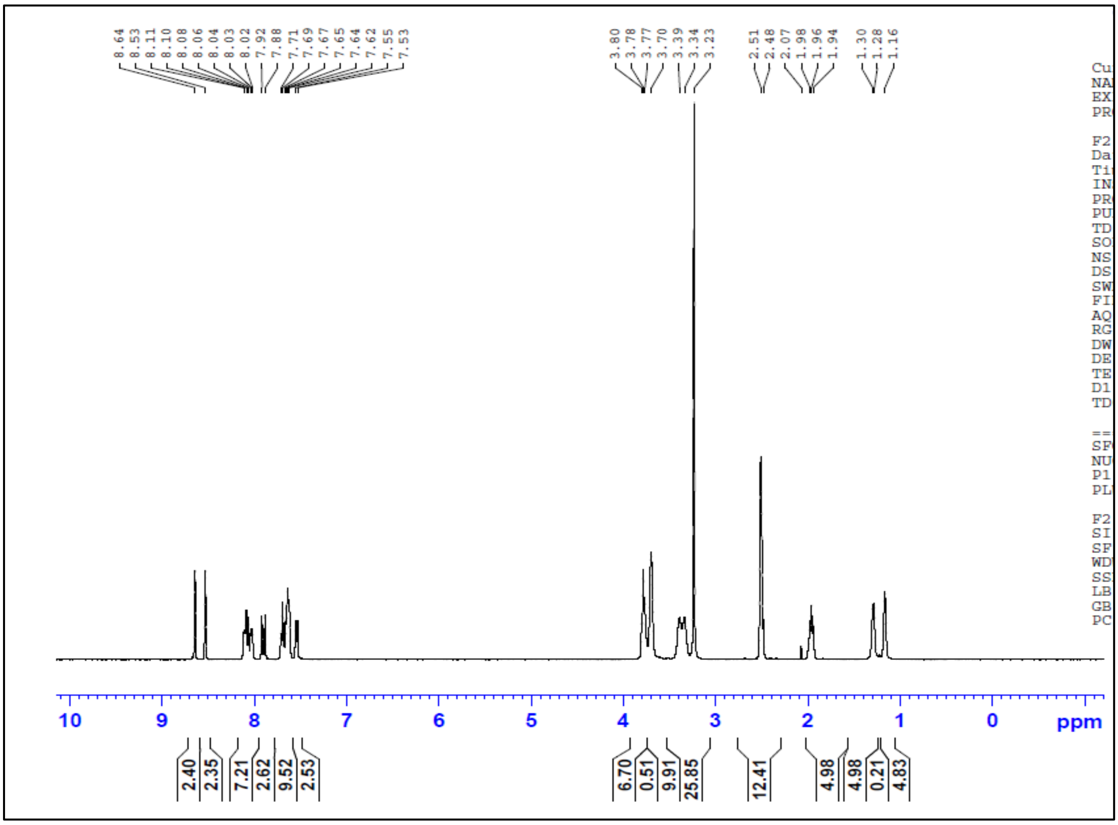

Figure S39: ^{1}HNMR spectrum of compound **3j** (400 MHz, DMSO-*d*6).

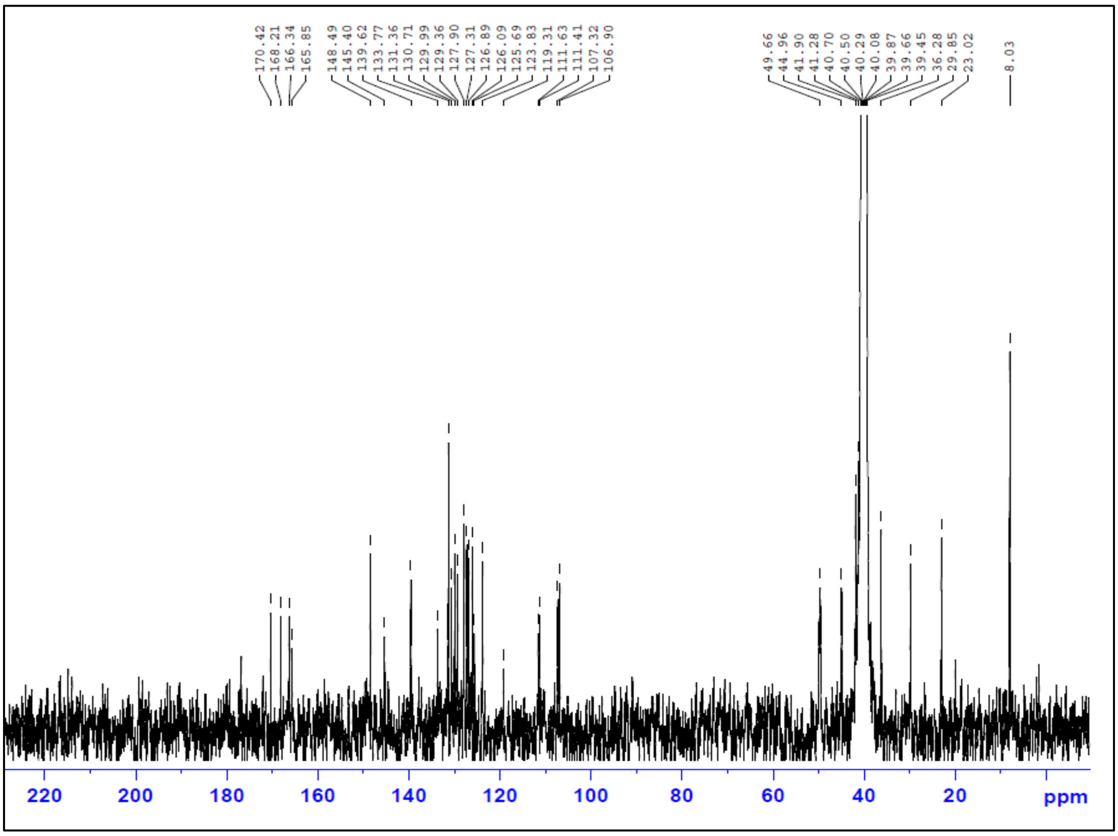

Figure S 40: ^{13}CNMR spectrum of compound **3j** (100 MHz, DMSO-*d*6).

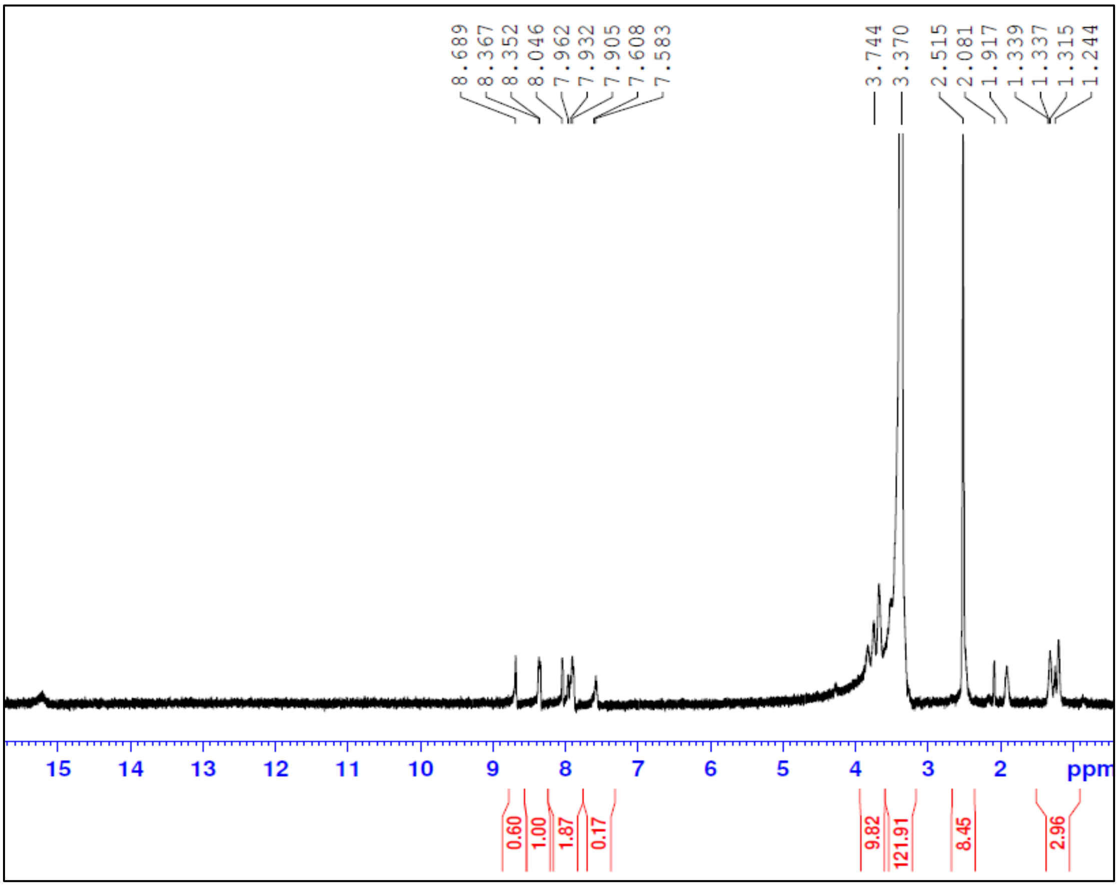

Figure S 41: ^{1}HNMR spectrum of compound **3k** (400 MHz, DMSO-*d*6).

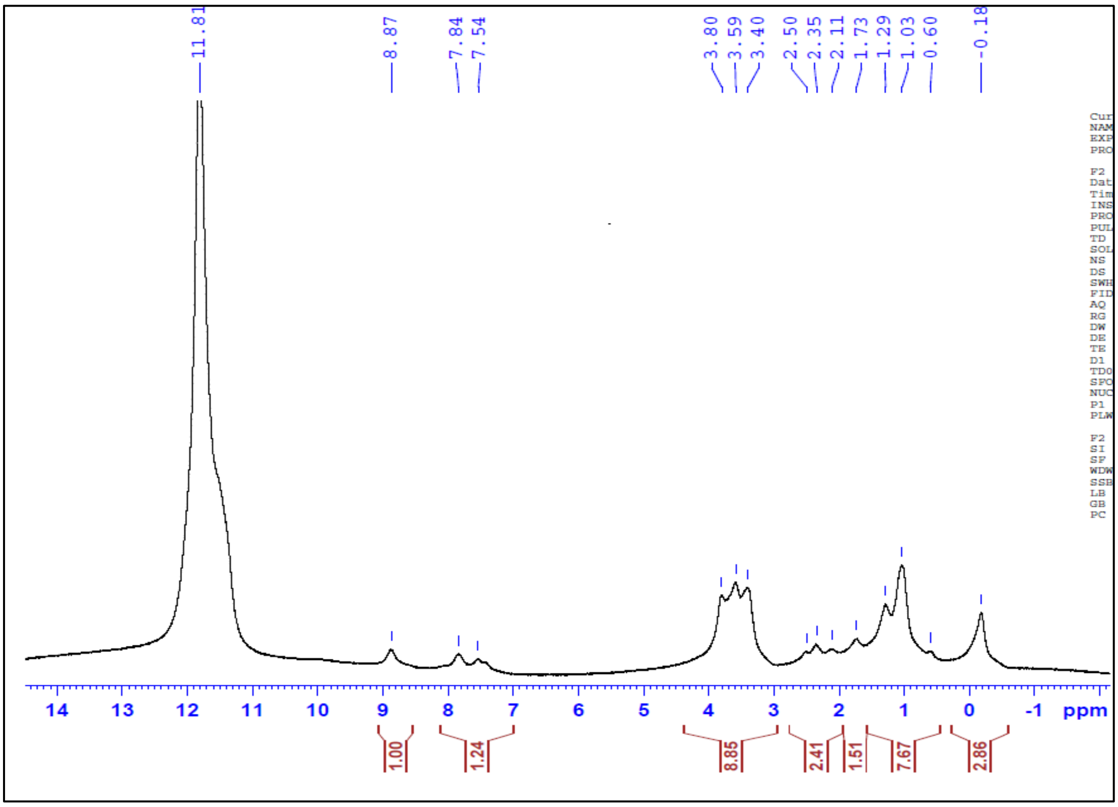

Figure S42: ^{1}HNMR spectrum of compound **3l** (400 MHz, CF_3COOH).

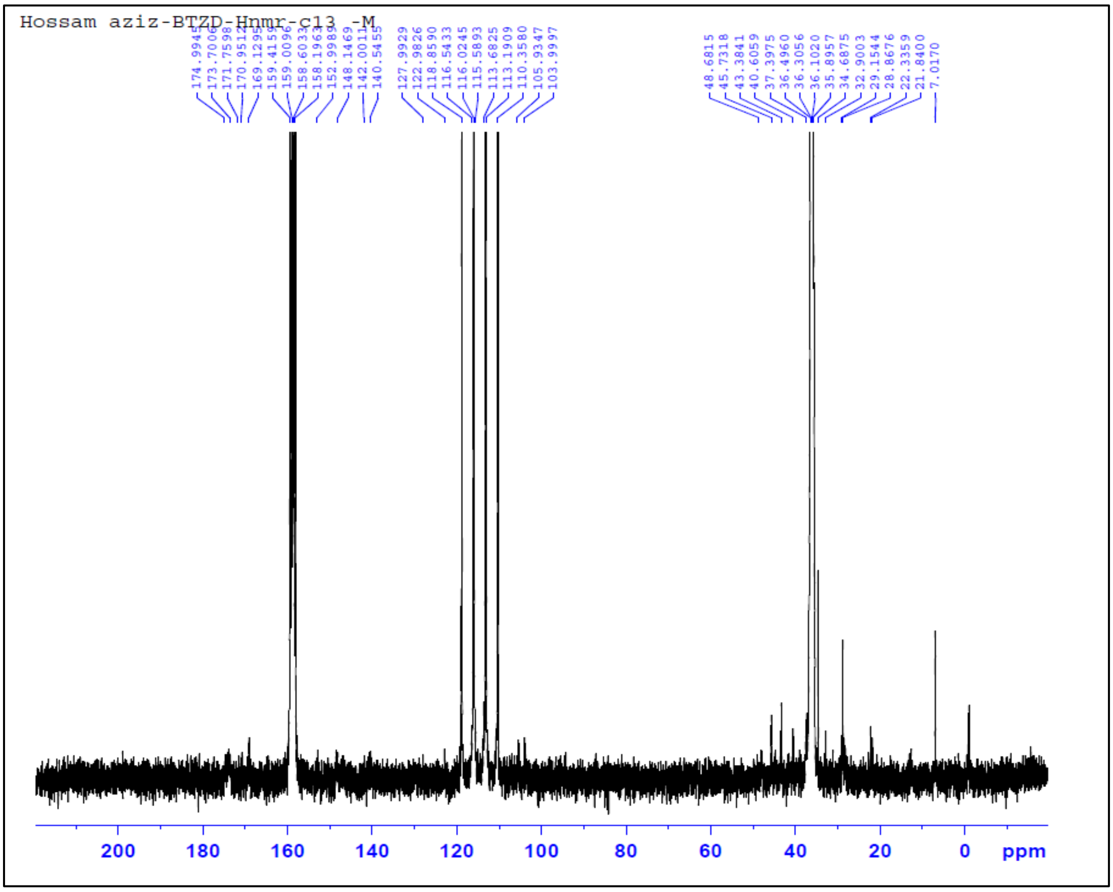

Figure S 43: ^{13}CNMR spectrum of compound **3l** (100 MHz, CF_3COOH).

Biology

Screening of anticancer activity of compounds **2** and **3a-3l**

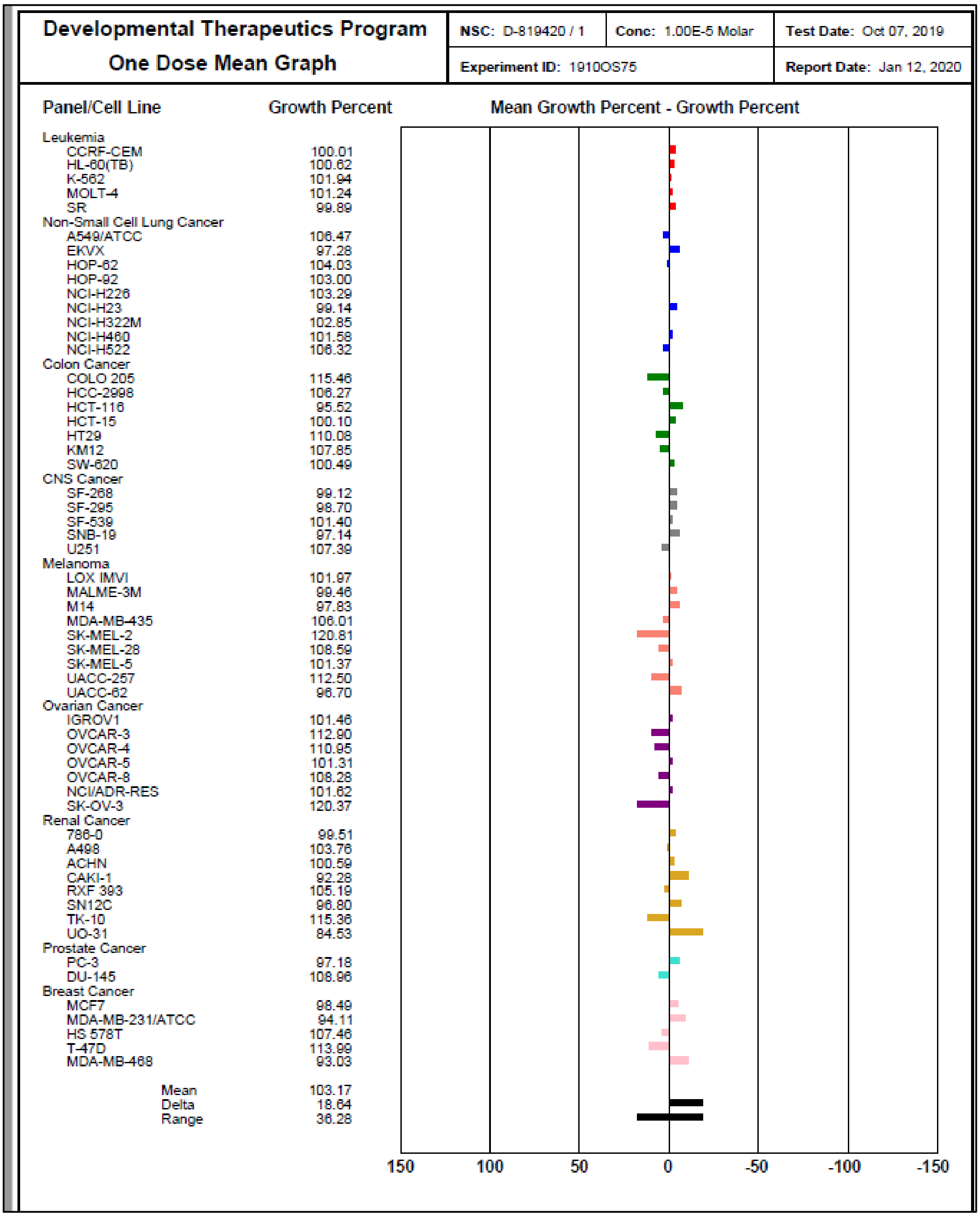

Figure S44: One dose growth (%) and mean graph for compound **2.**

Developmental Therapeutics Program One Dose Mean Graph	NSC: D-814888 / 1	Conc: 1.00E-5 Molar	Test Date: May 06, 2019
	Experiment ID: 1905OS01		Report Date: Aug 07, 2019

Panel/Cell Line	Growth Percent
Leukemia	
CCRF-CEM	90.22
HL-60(TB)	100.60
K-562	77.50
MOLT-4	87.37
RPMI-8226	88.64
SR	70.10
Non-Small Cell Lung Cancer	
A549/ATCC	85.76
EKVX	103.62
HOP-62	103.99
HOP-92	105.83
NCI-H23	89.83
NCI-H322M	90.61
NCI-H460	96.28
NCI-H522	72.63
Colon Cancer	
COLO 205	113.76
HCC-2998	98.00
HCT-116	80.65
HCT-15	96.46
HT29	105.96
KM12	96.07
SW-620	103.92
CNS Cancer	
SF-268	87.59
SF-295	94.41
SF-539	91.16
SNB-19	90.22
SNB-75	70.93
U251	97.23
Melanoma	
LOX IMVI	46.75
MALME-3M	69.58
M14	91.77
MDA-MB-435	92.15
SK-MEL-2	89.91
SK-MEL-28	91.05
SK-MEL-5	75.37
UACC-257	78.92
UACC-62	71.38
Ovarian Cancer	
IGROV1	91.45
OVCAR-3	96.33
OVCAR-4	97.48
OVCAR-5	109.68
OVCAR-8	84.57
NCI/ADR-RES	95.61
SK-OV-3	89.13
Renal Cancer	
786-0	78.79
A498	58.60
ACHN	85.55
CAKI-1	89.07
RXF 393	106.29
SN12C	97.42
TK-10	115.29
UO-31	64.05
Prostate Cancer	
PC-3	103.16
DU-145	109.54
Breast Cancer	
MCF7	44.07
MDA-MB-231/ATCC	74.75
HS 578T	91.23
BT-549	92.75
T-47D	91.94
MDA-MB-468	87.06
Mean	88.98
Delta	44.91
Range	71.22

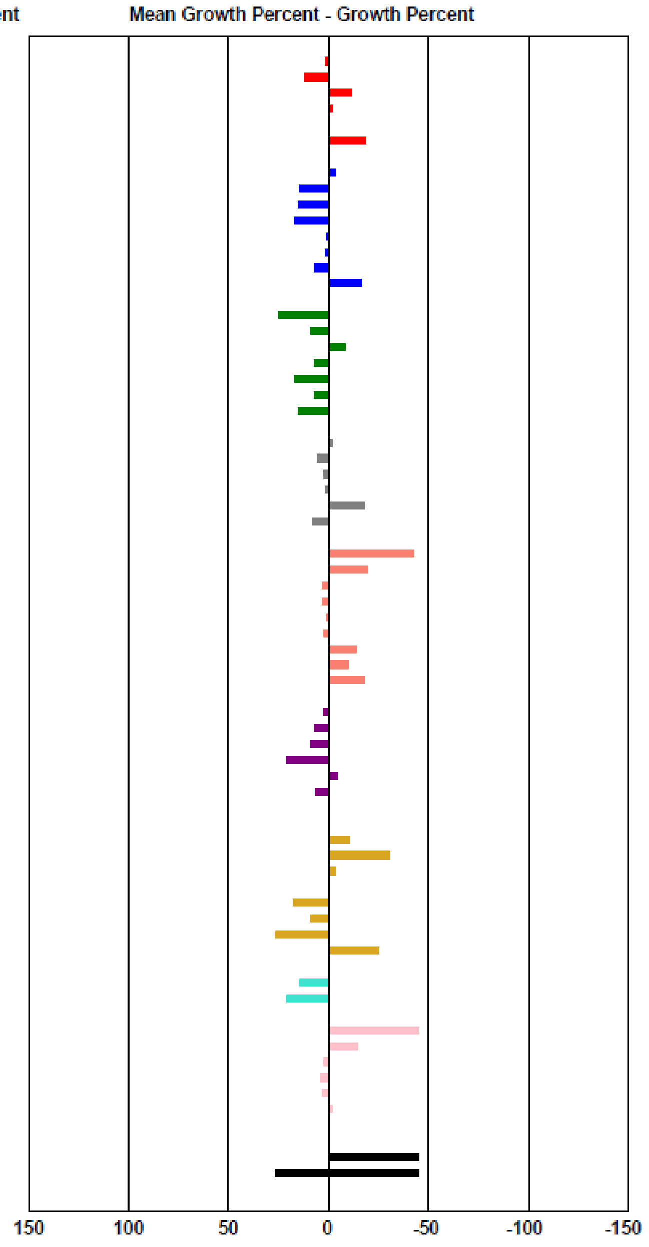

Figure S45: One dose growth (%) and mean graph for compound **3a.**

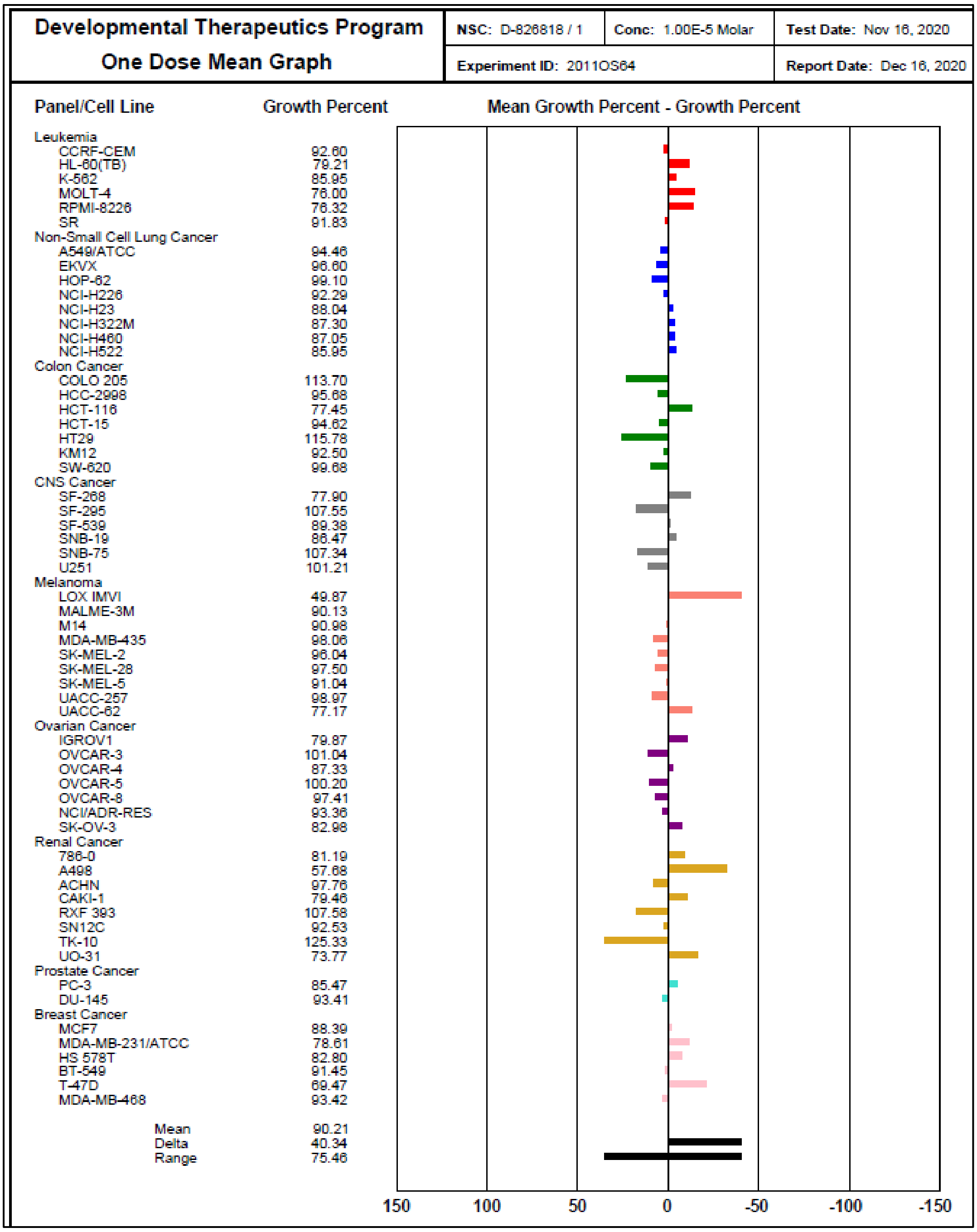

Figure S46: One dose growth (%) and mean graph for compound **3b.**

Developmental Therapeutics Program	NSC: D-819419 / 1	Conc: 1.00E-5 Molar	Test Date: Oct 07, 2019
One Dose Mean Graph	Experiment ID: 1910OS75		Report Date: Jan 12, 2020

Panel/Cell Line	Growth Percent
Leukemia	
CCRF-CEM	97.10
HL-60(TB)	93.76
K-562	101.66
MOLT-4	100.27
SR	102.74
Non-Small Cell Lung Cancer	
A549/ATCC	106.63
EKVX	97.62
HOP-62	107.12
HOP-92	105.18
NCI-H226	104.00
NCI-H23	98.18
NCI-H322M	108.58
NCI-H460	104.70
NCI-H522	98.30
Colon Cancer	
COLO 205	114.09
HCC-2998	104.95
HCT-116	104.36
HCT-15	106.50
HT29	109.43
KM12	104.19
SW-620	103.03
CNS Cancer	
SF-268	96.32
SF-295	97.76
SF-539	99.10
SNB-19	100.91
U251	103.00
Melanoma	
LOX IMVI	96.52
MALME-3M	101.11
M14	105.76
MDA-MB-435	109.49
SK-MEL-2	105.71
SK-MEL-28	106.56
SK-MEL-5	96.19
UACC-257	106.41
UACC-62	95.76
Ovarian Cancer	
IGROV1	96.84
OVCAR-3	106.95
OVCAR-4	110.64
OVCAR-5	101.70
OVCAR-8	105.79
NCI/ADR-RES	103.13
SK-OV-3	116.65
Renal Cancer	
786-0	99.09
A498	94.67
ACHN	97.68
CAKI-1	98.52
RXF 393	92.83
SN12C	100.50
TK-10	115.63
UO-31	83.52
Prostate Cancer	
PC-3	98.88
DU-145	105.85
Breast Cancer	
MCF7	101.68
MDA-MB-231/ATCC	101.59
HS 578T	107.81
T-47D	112.30
MDA-MB-468	108.89
Mean	102.70
Delta	19.18
Range	33.13

Mean Growth Percent - Growth Percent

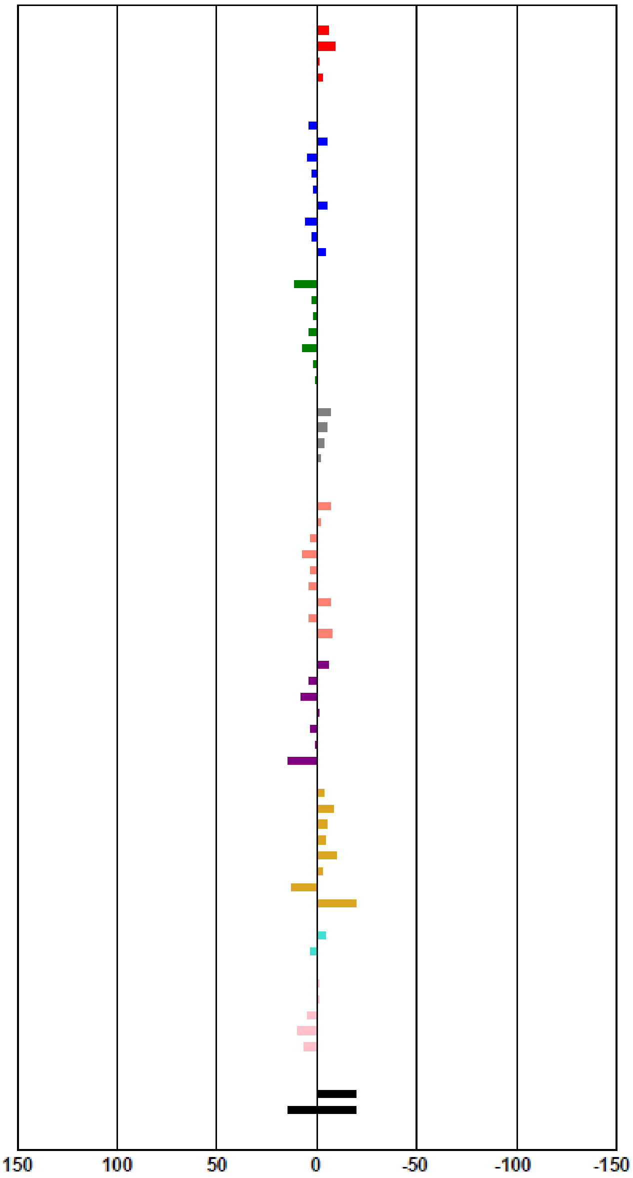

Figure S47: One dose growth (%) and mean graph for compound **3c**

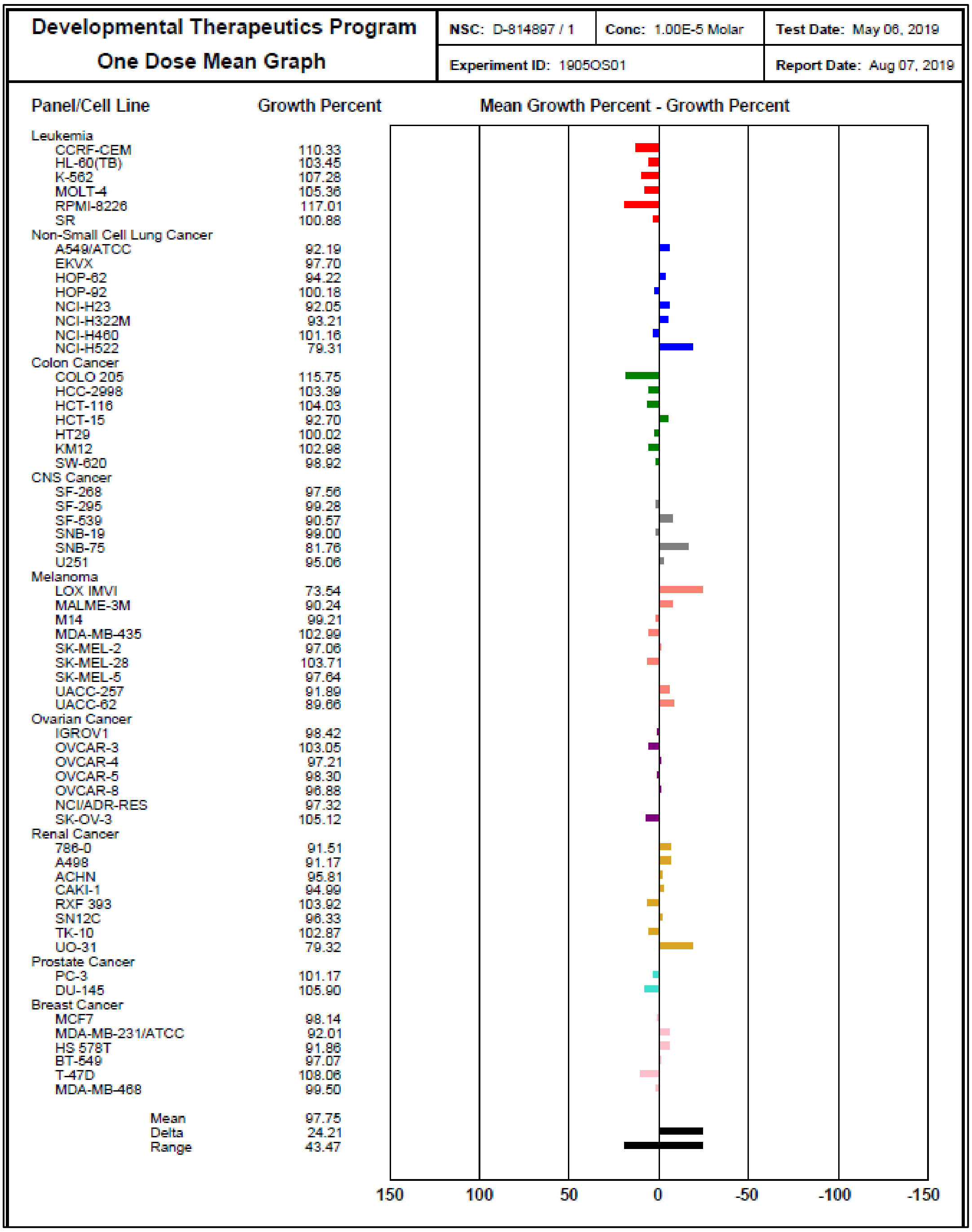

Figure S48: One dose growth (%) and mean graph for compound **3d.**

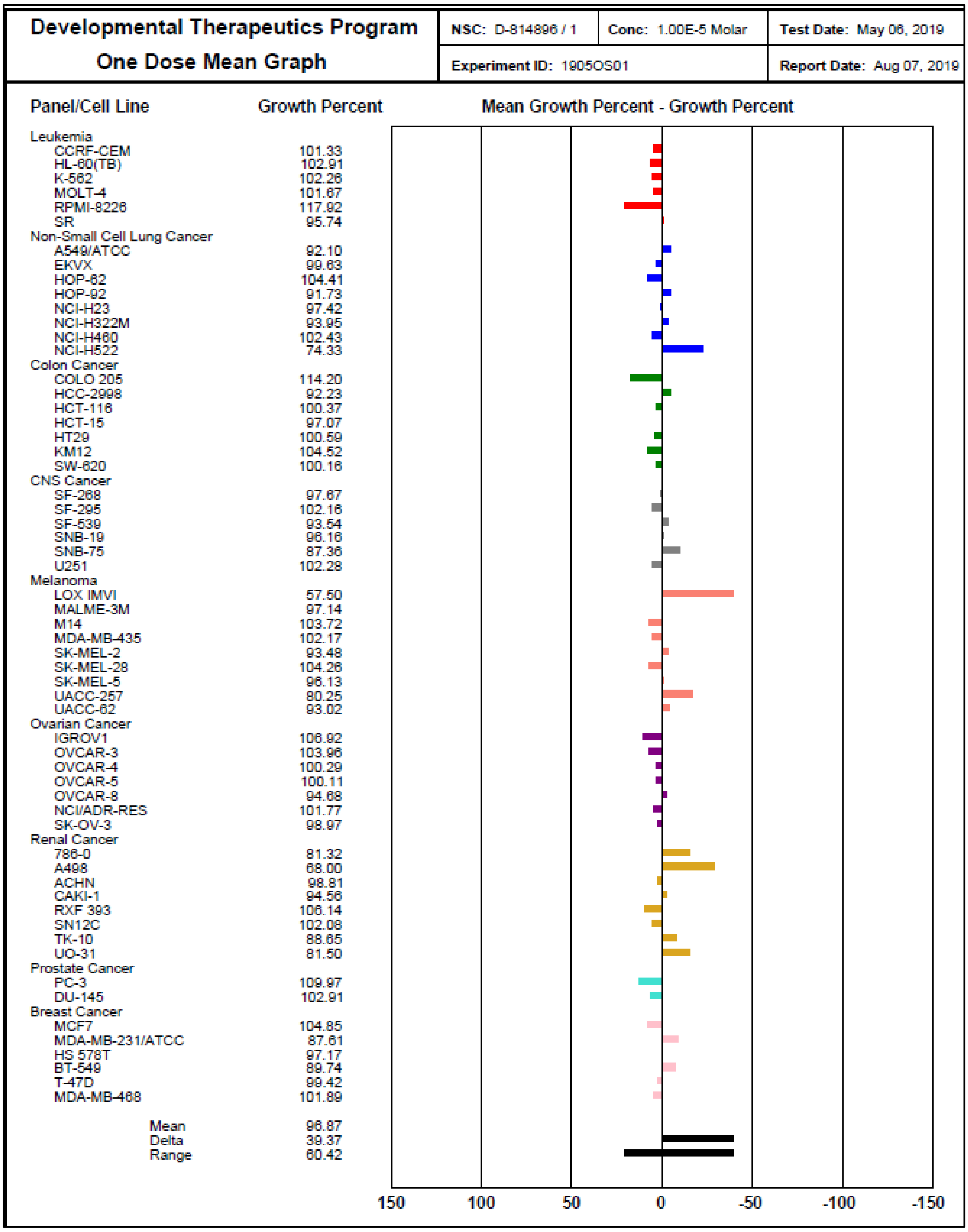

Figure S49: One dose growth (%) and mean graph for compound **3e**.

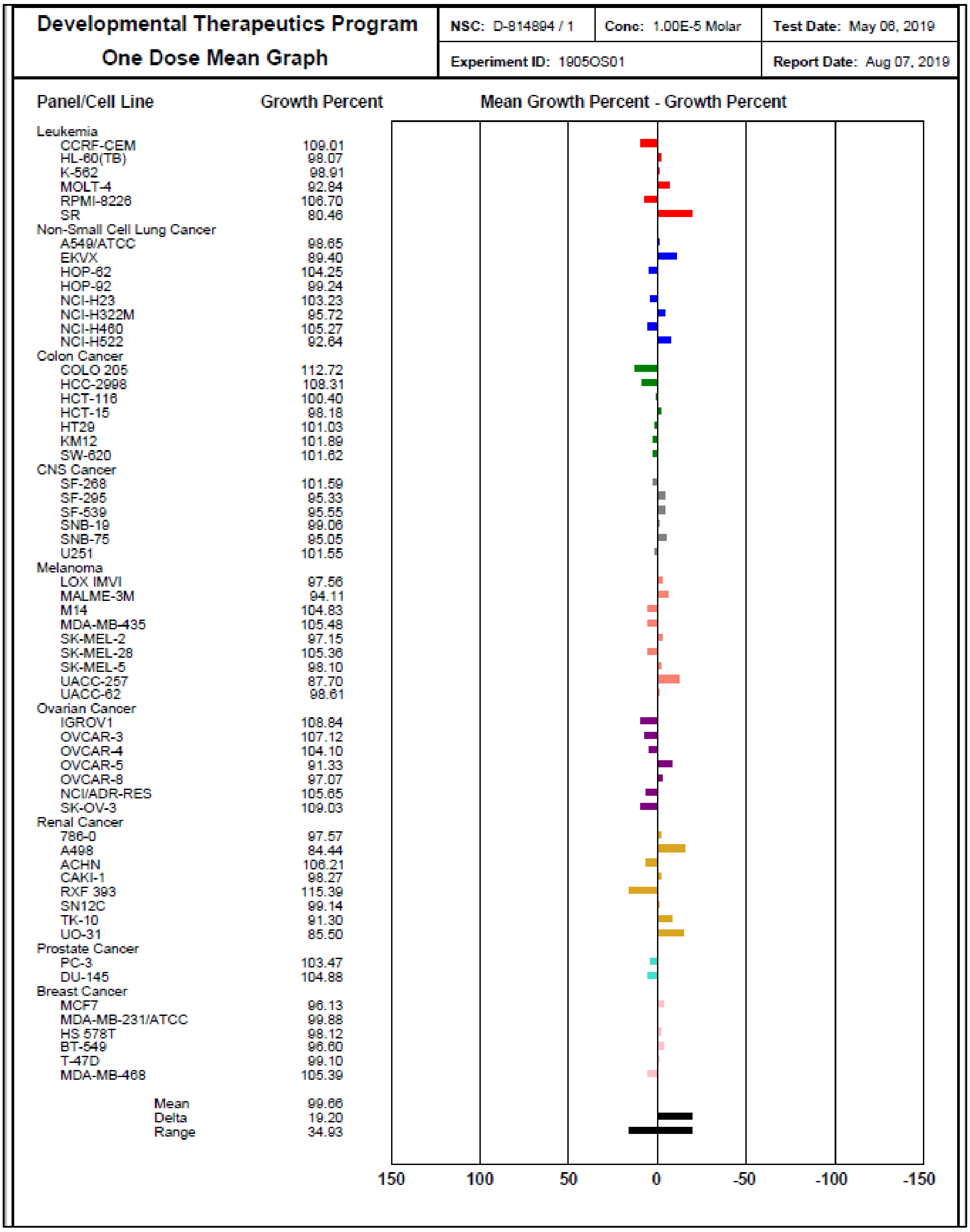

Figure S50: One dose growth (%) and mean graph for compound **3f**.

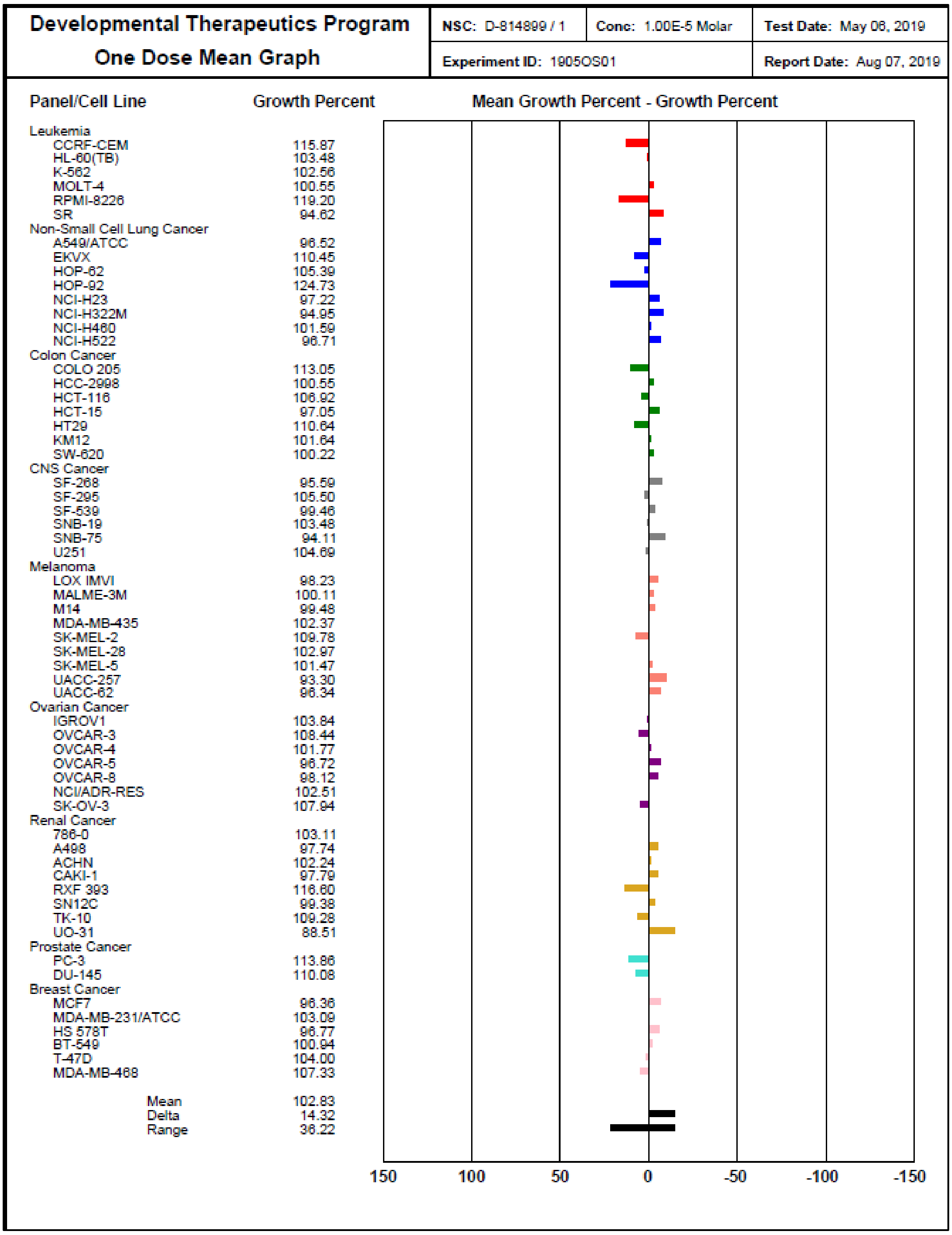

Figure S51: One dose growth (%) and mean graph for compound **3g**.

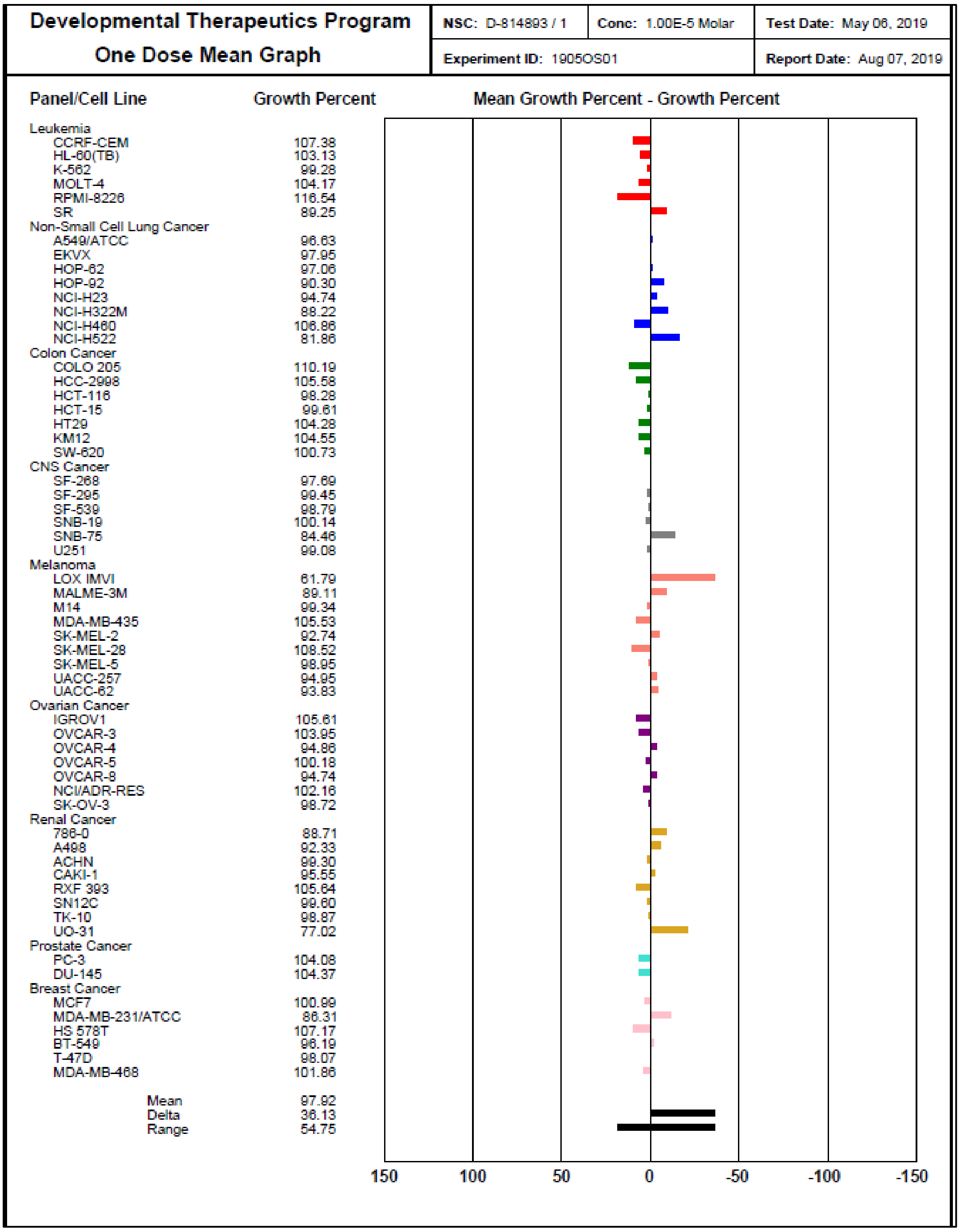

Figure S52: One dose growth (%) and mean graph for compound 3h.

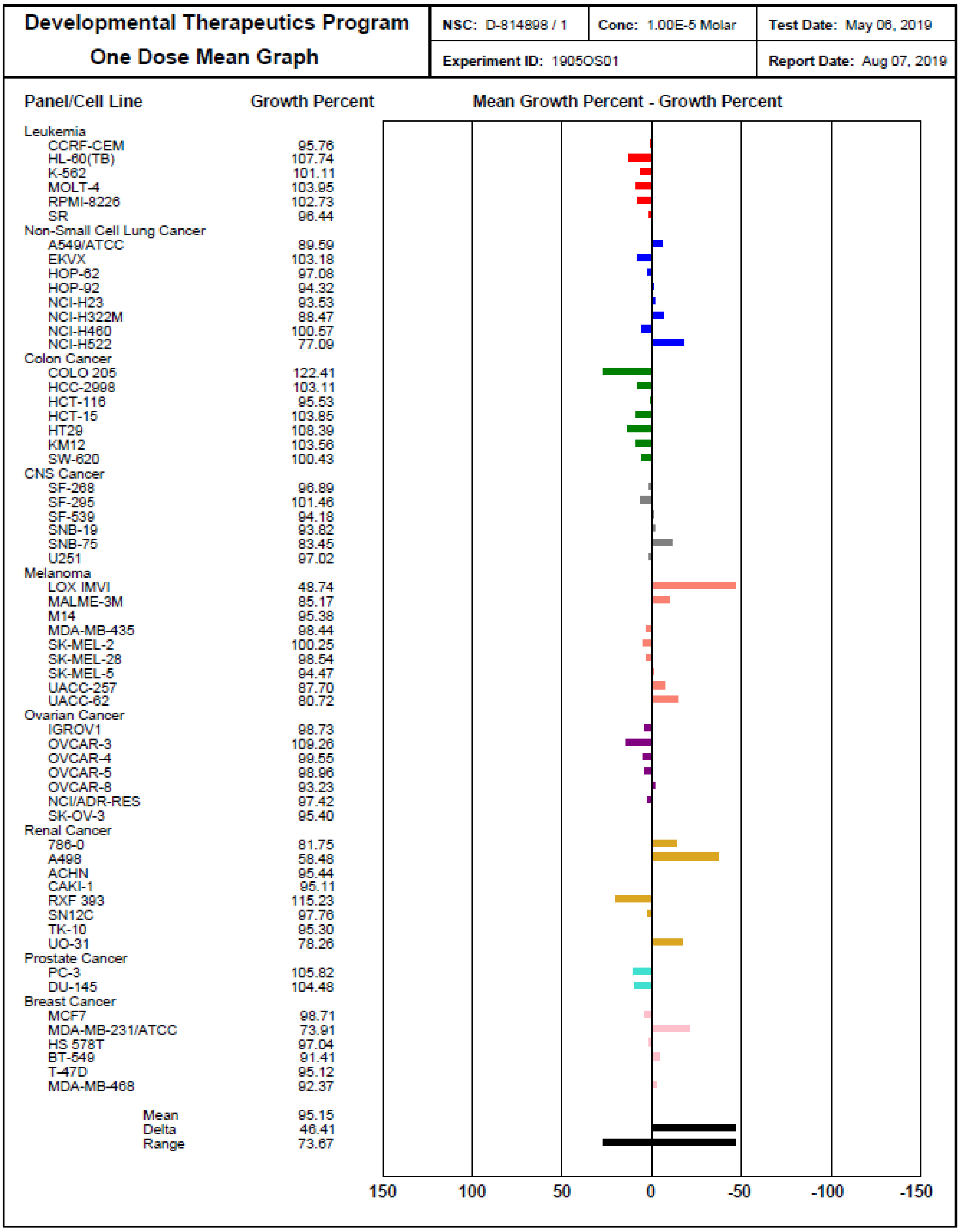

Figure S53: One dose growth (%) and mean graph for compound **3i**

Developmental Therapeutics Program

One Dose Mean Graph

NSC: D-814895 / 1	Conc: 1.00E-5 Molar	Test Date: May 06, 2019
Experiment ID: 1905OS01		Report Date: Aug 07, 2019

Panel/Cell Line	Growth Percent
Leukemia	
CCRF-CEM	100.83
HL-60(TB)	100.81
K-562	89.07
MOLT-4	82.54
RPMI-8226	93.97
SR	85.91
Non-Small Cell Lung Cancer	
A549/ATCC	90.65
EKVX	90.98
HOP-62	84.87
HOP-92	86.68
NCI-H23	86.87
NCI-H322M	73.41
NCI-H460	96.09
NCI-H522	81.46
Colon Cancer	
COLO 205	102.31
HCC-2998	102.79
HCT-116	89.39
HCT-15	94.38
HT29	97.19
KM12	95.28
SW-620	99.42
CNS Cancer	
SF-268	70.07
SF-295	97.27
SF-539	90.11
SNB-19	79.89
SNB-75	71.56
U251	92.40
Melanoma	
LOX IMVI	61.46
MALME-3M	86.88
M14	83.32
MDA-MB-435	83.21
SK-MEL-2	101.25
SK-MEL-28	95.50
SK-MEL-5	53.87
UACC-257	80.96
UACC-62	79.64
Ovarian Cancer	
IGROV1	74.11
OVCAR-3	100.80
OVCAR-4	83.56
OVCAR-5	90.95
OVCAR-8	91.01
NCI/ADR-RES	88.47
SK-OV-3	89.04
Renal Cancer	
786-0	81.45
A498	49.14
ACHN	88.47
CAKI-1	82.02
RXF 393	88.16
SN12C	75.90
TK-10	105.58
UO-31	51.90
Prostate Cancer	
PC-3	102.45
DU-145	78.05
Breast Cancer	
MCF7	58.29
MDA-MB-231/ATCC	74.81
HS 578T	69.71
BT-549	79.51
T-47D	92.33
MDA-MB-468	75.29
Mean	85.14
Delta	36.00
Range	56.44

Mean Growth Percent - Growth Percent

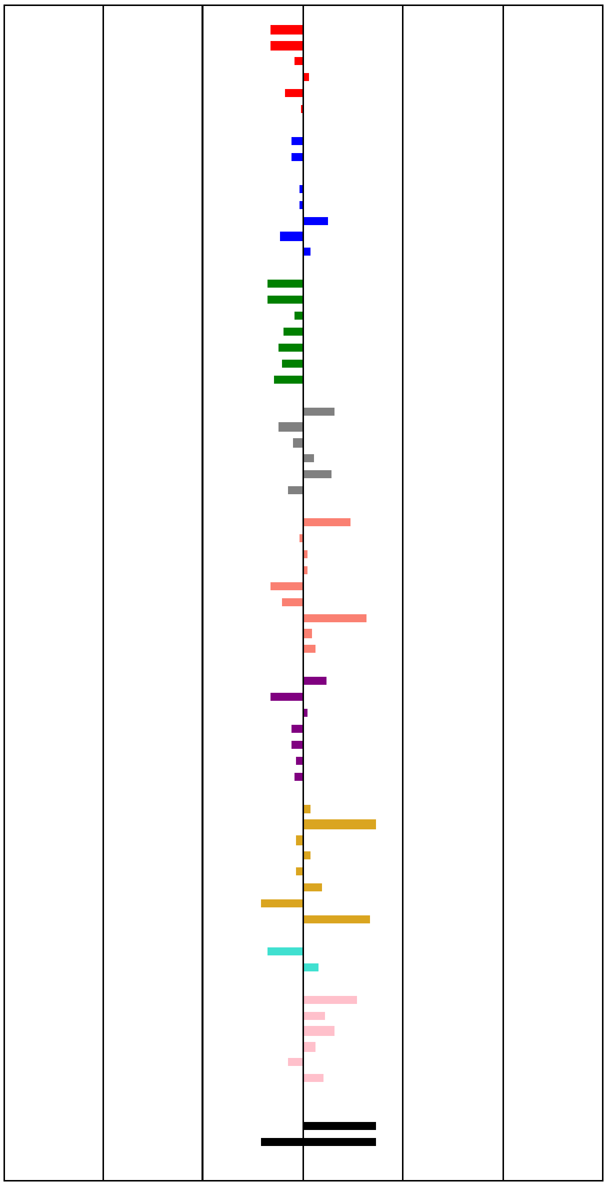

Figure S54: One dose growth (%) and mean graph for compound **3j**

Developmental Therapeutics Program One Dose Mean Graph	NSC: D-827499 / 1	Conc: 1.00E-5 Molar	Test Date: Jan 04, 2021
	Experiment ID: 2101OS70		Report Date: Feb 01, 2021

Panel/Cell Line	Growth Percent
**Leukemia**	
HL-60(TB)	133.59
K-562	105.83
MOLT-4	106.84
RPMI-8226	91.33
SR	90.34
**Non-Small Cell Lung Cancer**	
A549/ATCC	94.52
EKVX	75.55
HOP-62	80.27
HOP-92	99.84
NCI-H226	94.21
NCI-H23	93.82
NCI-H322M	107.80
NCI-H460	107.96
**Colon Cancer**	
COLO 205	114.73
HCC-2998	117.91
HCT-116	104.55
HCT-15	113.75
HT29	112.97
KM12	101.26
SW-620	98.21
**CNS Cancer**	
SF-268	86.68
SF-295	102.83
SF-539	94.02
SNB-19	100.65
SNB-75	57.54
U251	110.55
**Melanoma**	
LOX IMVI	91.12
MALME-3M	105.00
M14	109.93
MDA-MB-435	101.79
SK-MEL-2	106.15
SK-MEL-28	101.21
SK-MEL-5	99.23
UACC-257	104.92
UACC-62	97.99
**Ovarian Cancer**	
IGROV1	84.06
OVCAR-3	105.31
OVCAR-4	86.34
OVCAR-5	93.44
OVCAR-8	104.99
NCI/ADR-RES	107.87
SK-OV-3	114.74
**Renal Cancer**	
786-0	85.89
A498	70.14
ACHN	99.94
CAKI-1	80.51
RXF 393	52.05
SN12C	91.28
TK-10	66.49
UO-31	58.91
**Prostate Cancer**	
PC-3	102.50
DU-145	92.95
**Breast Cancer**	
MCF7	100.37
MDA-MB-231/ATCC	88.82
HS 578T	98.99
T-47D	110.26
MDA-MB-468	102.75
Mean	96.73
Delta	44.68
Range	81.54

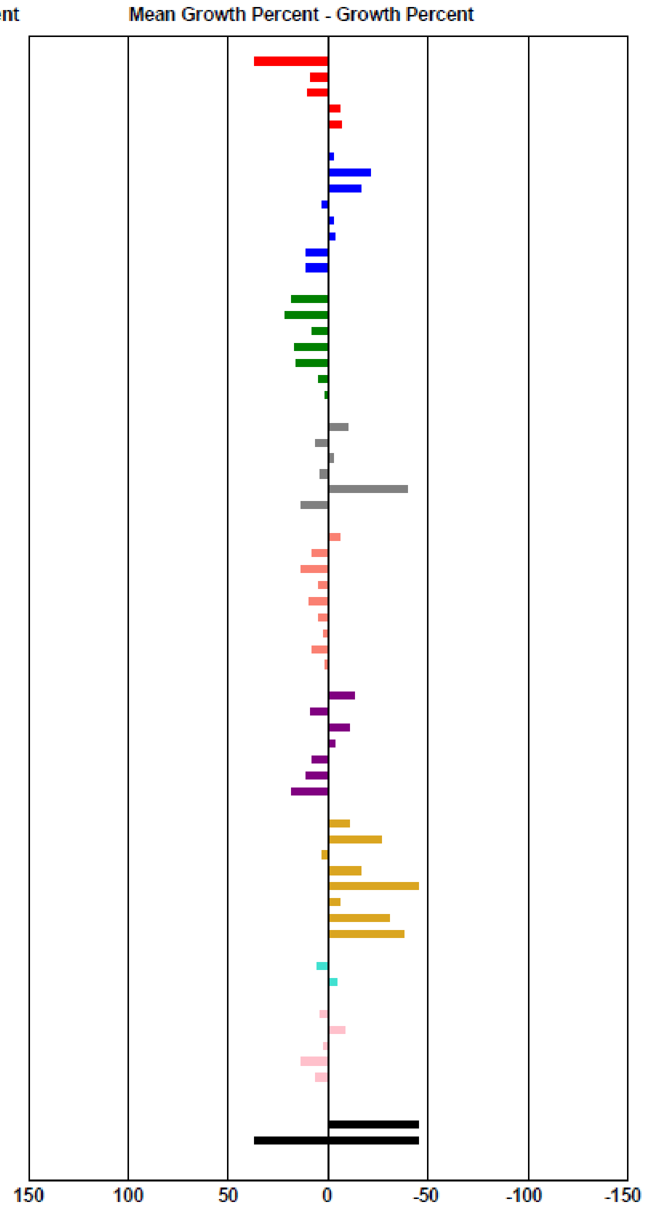

Figure S55: One dose growth (%) and mean graph for compound **3k**

Developmental Therapeutics Program | **NSC:** D-827500 / 1 | **Conc:** 1.00E-5 Molar | **Test Date:** Jan 04, 2021

One Dose Mean Graph | **Experiment ID:** 2101OS70 | **Report Date:** Feb 01, 2021

Panel/Cell Line	Growth Percent
Leukemia	
HL-60(TB)	124.95
K-562	106.07
MOLT-4	117.77
RPMI-8226	93.95
SR	106.13
Non-Small Cell Lung Cancer	
A549/ATCC	93.27
EKVX	76.23
HOP-62	83.22
HOP-92	99.29
NCI-H226	100.63
NCI-H23	94.31
NCI-H322M	102.51
NCI-H460	107.97
Colon Cancer	
COLO 205	114.95
HCC-2998	114.70
HCT-116	107.37
HCT-15	113.43
HT29	107.89
KM12	100.21
SW-620	103.87
CNS Cancer	
SF-268	84.92
SF-295	100.94
SF-539	96.77
SNB-19	101.23
SNB-75	48.87
U251	113.72
Melanoma	
LOX IMVI	100.65
MALME-3M	101.84
M14	111.00
MDA-MB-435	104.54
SK-MEL-2	111.07
SK-MEL-28	94.54
SK-MEL-5	100.21
UACC-257	104.01
UACC-62	91.64
Ovarian Cancer	
IGROV1	84.73
OVCAR-3	112.14
OVCAR-4	80.81
OVCAR-5	88.84
OVCAR-8	103.33
NCI/ADR-RES	106.19
SK-OV-3	116.40
Renal Cancer	
786-0	95.20
A498	54.94
ACHN	99.42
CAKI-1	73.32
RXF 393	69.37
SN12C	85.43
TK-10	71.31
UO-31	58.03
Prostate Cancer	
PC-3	97.62
DU-145	91.69
Breast Cancer	
MCF7	101.29
MDA-MB-231/ATCC	89.93
HS 578T	95.22
T-47D	104.60
MDA-MB-468	97.85
Mean	96.71
Delta	47.84
Range	76.08

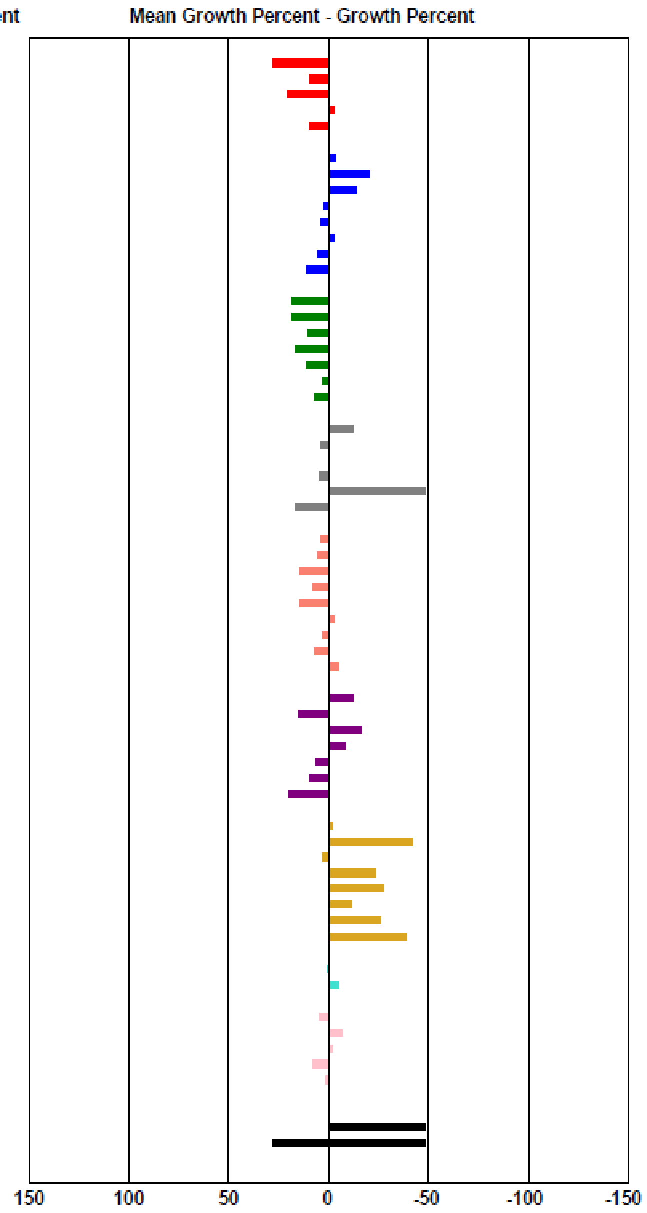

Figure S56: One dose growth (%) and mean graph for compound **3l**

2- Biological evaluation
Cytotoxicity results

Cell culture Protocol

Cell Line cells were obtained from American Type Culture Collection, cells were cultured using DMEM (Invitrogen/Life Technologies) supplemented with 10% FBS (Hyclone,), 10 ug/ml of insulin (Sigma), and 1% penicillin-streptomycin. All of the other chemicals and reagents were from Sigma, or Invitrogen.
Plate cells (cells density 1.2 – 1.8 × 10,000 cells/well) in a volume of 100µl complete growth medium + 100 ul of the tested compound per well in a 96-well plate for 24 hours before the MTT assay.

Cell culture protocol
1. Remove culture medium to a centrifuge tube.
2. Briefly rinse the cell layer with 0.25% (w/v) Trypsin 0.53 mM EDTA solution to remove all traces of serum which contains Trypsin inhibitor.
3. Add 2.0 to 3.0 ml of Trypsin EDTA solution to flask and observe cells under an inverted microscope until cell layer is dispersed (usually within 5 to 15 minutes).
Note: To avoid clumping do not agitate the cells by hitting or shaking the flask while waiting for the cells to detach. Cells that are difficult to detach may be placed at 37°C to facilitate dispersal.
4. Add 6.0 to 8.0 mL of complete growth medium and aspirate cells by gently pipetting.
5. Transfer the cell suspension to the centrifuge tube with the medium and cells from step 1, and centrifuge at approximately 125 xg for 5 to 10 minutes. Discard the supernatant.
6. Resuspend the cell pellet in fresh growth medium. Add appropriate aliquots of the cell suspension to new culture vessels.
7. Incubate cultures at 37°C for 24 h.
8-After treatment of cells with the serial concentrations of the compound to be tested incubation is carried out for 48 h at 37ºC, then the plates are to be examined under the inverted microscope and proceed for the MTT assay

MTT – Cytotoxicity assay protocol

MTT assay for cell viability
Cells were cultured using DMEM (Invitrogen/Life Technologies) supplemented with 10% FBS (Hyclone,), 10 ug/ml of insulin (Sigma), and 1% penicillin-streptomycin. Plate cells (cells density 1.2 – 1.8 × 10,000 cells/well) in a volume of 100µl complete growth medium + 100 ul of the tested compound per well in a 96-well plate for 24 hours before the MTT assay. In atypical experiment, one hundred micro liters of serial 10-fold diluted sterile tested compounds were added to final concentrations of 0.01e100 mM using culture media as

negative control. After 24 h of culture incubation and supernatants discarded. HL-60 (TB), HCT-116 and MCF cell lines were trypsinized and washed with Ca2/Mg2 free PBS (pH 7.2). We removed cultures from incubator into laminar flow hood or other sterile work area. Cells in the log phase of growth should be employed and final cell number should not exceed 106 cells/cm^2. Each test should include a blank containing complete medium without cells. Reconstitute each vial of MTT [M-5655] to be used with 3 ml of medium or balanced salt solution without phenol red and serum. Add reconstituted MTT in an amount equal to 10% of the culture medium volume. Return cultures to incubator for 2-4 hours depending on cell type and maximum cell density. After the incubation period, remove cultures from incubator and dissolve the resulting formazan crystals by adding an amount of MTT Solubilization Solution [M-8910] equal to the original culture medium volume. Spectrophotometrically absorbance was measured at a wave length of 570 nm. Measure the background absorbance of multiwell plates at 690 nm and subtract from the 450 nm measurement. Results from all experiments were recorded and the percentage of viable cells was calculated.

Dr. Mohamed badr
Assay : MTT cytotoxicity assay
Samples : 06 compounds.
Reader : BIOLINE ELIZA READER wl 450 nm
Solvent : DMSO

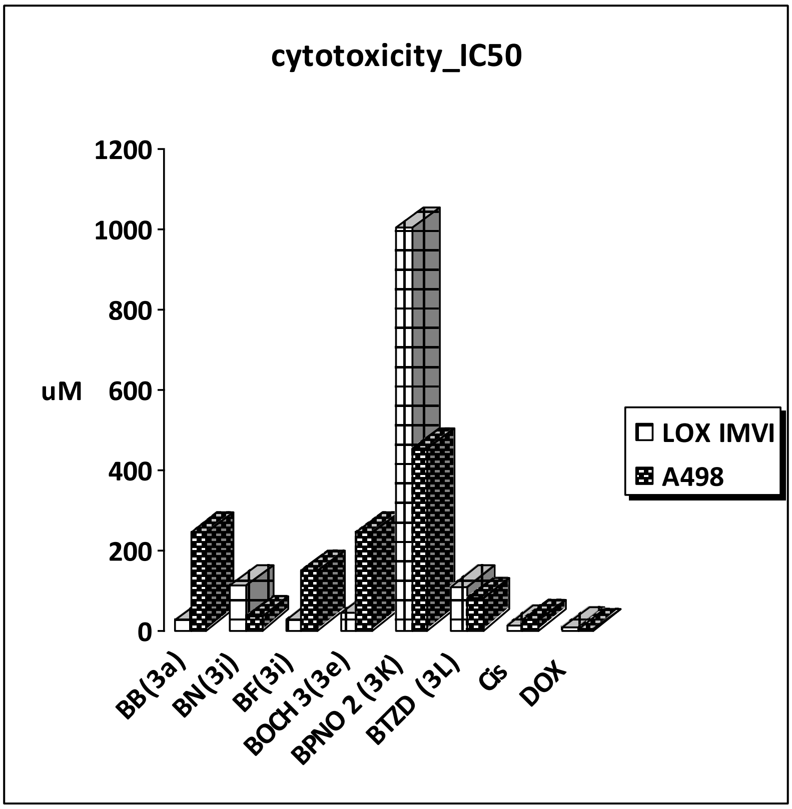

Figure S57: IC $_{50}$ of compounds **3a, 3e, 3i, 3j, 3k, 3l**, **cisplatin** and **doxorubicin** against LOX-IMVI and A 498 cancer cell lines.

Table S 1: Calculation of IC $_{50}$ of compounds **3a** and **3j** against LOX-IMVI

researcher	assay	Date	cells		
Dr. Mohamed badr	MTT	12-Jun		LOX-IMVI	A498

	Blank	CC	Sample No.		BB(3a)/LOX- IMVI			Sample No.		BN(3j)/LOX- IMVI		
	1	2	3	4	5	6	7	8	9	10	11	12
A	B	C	100Um	25Um	6.3Um	1.6Um	0.4Um	100Um	25Um	6.3Um	1.6Um	0.4Um
B	B	C	100Um	25Um	6.3Um	1.6Um	0.4Um	100Um	25Um	6.3Um	1.6Um	0.4Um
C	B	C	100Um	25Um	6.3Um	1.6Um	0.4Um	100Um	25Um	6.3Um	1.6uM	0.4uM

ROBONIK P2000 Eia reader

Wave length: 450 nm

Reference: 630 nm

	1	2	3	4	5	6	7	8	9	10	11	12
A	0	0.559	0.231	0.275	0.342	0.369	0.431	0.271	0.327	0.376	0.431	0.482
B	0	0.542	0.222	0.262	0.351	0.381	0.427	0.264	0.337	0.391	0.441	0.464
C	0	0.537	0.209	0.263	0.348	0.384	0.418	0.259	0.343	0.404	0.429	0.471
mean	0	0.546	0.2207	0.2667	0.347	0.378	0.4253	0.2647	0.336	0.39	0.434	0.4723
%		`	40.415	48.84	63.553	69.231	77.9	48.474	61.48	71.49	79.43	86.508

BB(3a)/LOX- IMVI

log conc.	% viability
2	40.4
1.398	48.8
0.796	63.6
0.193	69.2
-0.41	77.9

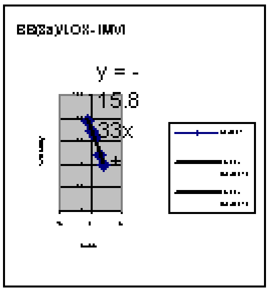

BN(3j)/LOX- IMVI

log conc.	% viability
2	48.47
1.3979	61.48
0.7959	71.49
0.1931	79.43
-0.409	86.51

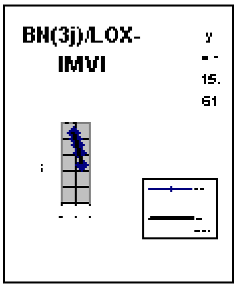

Table S 2: Calculation of IC $_{50}$ of compounds **3i** and **3e** against LOX-IMVI

	Blank	CC	Sample No.		BF(3i)/LOX- IMVI			Sample No.		BOCH3(3e)/LOX- IMVI		
	1	2	3	4	5	6	7	8	9	10	11	12
A	B	C	100uM	25uM	6.3uM	1.6uM	0.4uM	100uM	25uM	6.3uM	1.6uM	0.4uM
B	B	C	100uM	25uM	6.3uM	1.6uM	0.4uM	100uM	25uM	6.3uM	1.6uM	0.4uM
C	B	C	100uM	25uM	6.3uM	1.6uM	0.4uM	100uM	25uM	6.3uM	1.6uM	0.4uM

ROBONIK P2000 Eia reader

Wave length: 450 nm

Reference: 630 nm

	1	2	3	4	5	6	7	8	9	10	11	12
A	0	0.682	0.288	0.351	0.384	0.466	0.518	0.318	0.356	0.402	0.464	0.524
B	0	0.679	0.276	0.357	0.388	0.457	0.526	0.331	0.349	0.389	0.455	0.519
C	0	0.691	0.282	0.349	0.394	0.468	0.493	0.324	0.362	0.392	0.471	0.531
mean	0	0.684	0.282	0.3523	0.3887	0.4637	0.5123	0.3243	0.356	0.394	0.463	0.5247
% viability			41.228	51.511	56.823	67.788	74.903	47.417	52	57.65	67.74	76.706

BF(3i)/LOX- IMVI

log conc.	% viability
2	41.2
1.398	51.5
0.796	56.8
0.193	67.8
-0.41	74.9

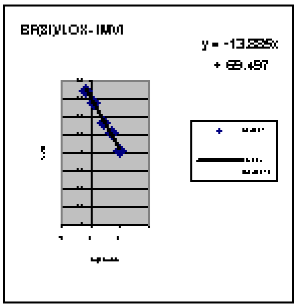

BOCH3(3e)/LOX- IMVI

log conc.	% viability
2	47.42
1.3979	52
0.7959	57.65
0.1931	67.74
-0.409	76.71

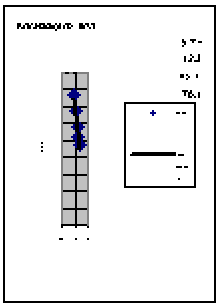

Table S 3: Calculation of IC $_{50}$ of compounds **3k** and **3l** against LOX-IMVI

	Blank	CC	Sample No.		BPNO2 (3K)/LOX- IMVI			Sample No.		BTZD (3L)/LOX- IMVI		
	1	2	3	4	5	6	7	8	9	10	11	12
A	B	C	100uM	25uM	6.3uM	1.6uM	0.4uM	100uM	25uM	6.3uM	1.6uM	0.4uM
B	B	C	100uM	25uM	6.3uM	1.6uM	0.4uM	100uM	25uM	6.3uM	1.6uM	0.4uM
C	B	C	100uM	25uM	6.3uM	1.6uM	0.4uM	100uM	25uM	6.3uM	1.6uM	0.4uM

ROBONIK P2000 Eia reader

Wave length: 450 nm

Reference: 630 nm

	1	2	3	4	5	6	7	8	9	10	11	12
A	0	0.581	0.391	0.463	0.532	0.583	0.628	0.282	0.343	0.398	0.443	0.492
B	0	0.558	0.376	0.454	0.559	0.575	0.619	0.277	0.346	0.404	0.452	0.477
C	0	0.579	0.357	0.461	0.542	0.591	0.641	0.285	0.339	0.411	0.461	0.488
mean	0	0.573	0.3747	0.4593	0.5443	0.583	0.6293	0.2813	0.343	0.404	0.452	0.4857
% viability			65.425	80.21	95.052	101.8	109.9	49.127	59.84	70.61	78.93	84.808

BPNO2 (3K)/LOX-IMVI

2	65.4
1.398	80.2
0.796	95.1
0.193	102
-0.41	110

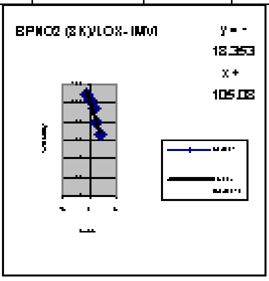

BTZD (3L)/LOX-IMVI

2	49.13
1.3979	59.84
0.7959	70.61
0.1931	78.93
-0.409	84.81

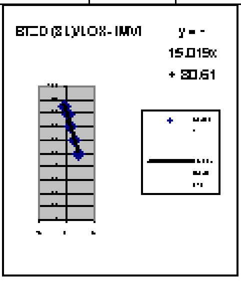

Table S 4: Calculation of IC $_{50}$ of compounds **cisplatin and doxorubicin** against LOX-IMVI

	Blank	CC	Sample No.		Cis/LOX- IMVI			Sample No.		DOX/LOX- IMVI		
	1	2	3	4	5	6	7	8	9	10	11	12
A	B	C	100uM	25uM	6.3uM	1.6uM	0.4uM	100uM	25uM	6.3uM	1.6uM	0.4uM

B	B	C	100uM	25uM	6.3uM	1.6uM	0.4uM	100uM	25uM	6.3uM	1.6uM	0.4uM
C	B	C	100uM	25uM	6.3uM	1.6uM	0.4uM	100uM	25uM	6.3uM	1.6uM	0.4uM

ROBONIK P2000 Eia reader

Wave length: 450 nm

Reference: 630 nm

	1	2	3	4	5	6	7	8	9	10	11	12
A	0	0.594	0.218	0.275	0.341	0.389	0.428	0.192	0.257	0.318	0.362	0.424
B	0	0.606	0.225	0.262	0.338	0.382	0.435	0.192	0.263	0.326	0.354	0.435
C	0	0.643	0.196	0.268	0.329	0.391	0.439	0.176	0.266	0.323	0.357	0.438
mean	0	0.614	0.213	0.2683	0.336	0.3873	0.434	0.1867	0.262	0.322	0.358	0.4323
% viability			34.672	43.679	54.693	63.049	70.646	30.385	42.65	52.47	58.22	70.374

Cis/LOX- IMVI

log conc.	% viability
2	34.7
1.398	43.7
0.796	54.7
0.193	63
-0.41	70.6

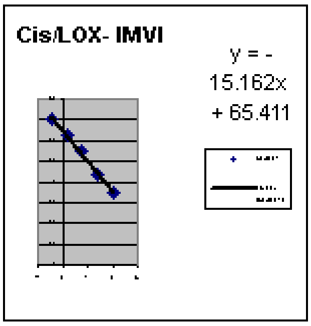

DOX/LOX- IMVI

log conc.	% viability
2	30.39
1.3979	42.65
0.7959	52.47
0.1931	58.22
-0.409	70.37

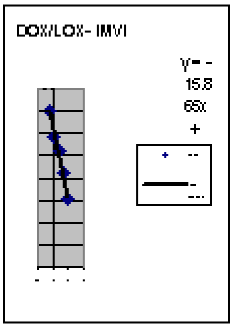

Table S 5: Calculation of IC $_{50}$ of compounds 3a and 3j against A498 cell line

	Blank	CC	Sample No. BB(3a)/A498					Sample No. BN(3j)/A498				
	1	2	3	4	5	6	7	8	9	10	11	12
A	B	C	100uM	25uM	6.3uM	1.6uM	0.4uM	100uM	25uM	6.3uM	1.6uM	0.4uM
B	B	C	100uM	25uM	6.3uM	1.6uM	0.4uM	100uM	25uM	6.3uM	1.6uM	0.4uM
C	B	C	100uM	25uM	6.3uM	1.6uM	0.4uM	100uM	25uM	6.3uM	1.6uM	0.4uM

ROBONIK P2000 Eia reader

Wave length: 450 nm

Reference: 630 nm

	1	2	3	4	5	6	7	8	9	10	11	12
A	0	0.529	0.292	0.364	0.432	0.474	0.532	0.218	0.259	0.319	0.364	0.432
B	0	0.494	0.285	0.352	0.411	0.481	0.525	0.222	0.267	0.324	0.369	0.428
C	0	0.513	0.284	0.349	0.428	0.469	0.518	0.213	0.264	0.331	0.371	0.441
mean	0	0.512	0.287	0.355	0.4237	0.4747	0.525	0.2177	0.263	0.325	0.368	0.4337
% viability			56.055	69.336	82.747	92.708	102.54	42.513	51.43	63.41	71.88	84.701

BB(3a)/A498

2	56.1
1.398	69.3
0.796	82.7
0.193	92.7

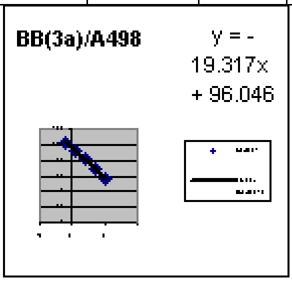

BN(3j)/A498

2	42.51
1.3979	51.43
0.7959	63.41
0.1931	71.88

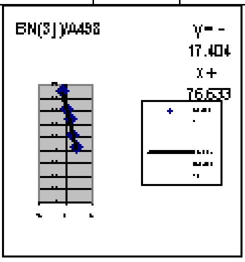

-0.41	103

-0.409	84.7

Table S 6: Calculation of IC $_{50}$ of compounds **3i and 3e** against A498 cell line

	Blank	CC	Sample No.		BF(3i)/A498			Sample No.		BOCH3(3e)/A498		
	1	2	3	4	5	6	7	8	9	10	11	12
A	B	C	100uM	25uM	6.3uM	1.6uM	0.4uM	100uM	25uM	6.3uM	1.6uM	0.4uM
B	B	C	100uM	25uM	6.3uM	1.6uM	0.4uM	100uM	25uM	6.3uM	1.6uM	0.4uM
C	B	C	100uM	25uM	6.3uM	1.6uM	0.4uM	100uM	25uM	6.3uM	1.6uM	0.4uM

ROBONIK P2000 Eia reader

Wave length: 450 nm

Reference: 630 nm

	1	2	3	4	5	6	7	8	9	10	11	12
A	0	0.509	0.264	0.338	0.381	0.427	0.482	0.294	0.359	0.414	0.474	0.515
B	0	0.524	0.274	0.329	0.379	0.433	0.505	0.292	0.366	0.406	0.466	0.539
C	0	0.517	0.272	0.341	0.381	0.435	0.497	0.285	0.351	0.428	0.482	0.522
mean	0	0.517	0.27	0.336	0.3803	0.4317	0.4947	0.2903	0.359	0.416	0.474	0.5253
% viability			52.258	65.032	73.613	83.548	95.742	56.194	69.42	80.52	91.74	101.68

BF(3i)/A498

2	52.3
1.398	65
0.796	73.6
0.193	83.5
-0.41	95.7

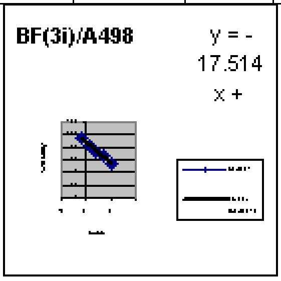

BOCH3(3e)/A498

2	56.19
1.3979	69.42
0.7959	80.52
0.1931	91.74
-0.409	101.7

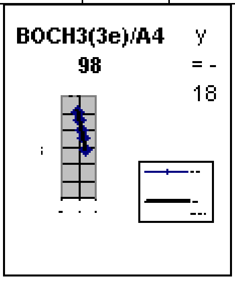

Table S 7: Calculation of IC $_{50}$ of compounds **3k and 3l** against A498 cell line

	Blank	CC	Sample No.		BPNO2 (3K)/A498			Sample No.		BTZD (3L)/A498		
	1	2	3	4	5	6	7	8	9	10	11	12
A	B	C	100uM	25uM	6.3uM	1.6uM	0.4uM	100uM	25uM	6.3uM	1.6uM	0.4uM
B	B	C	100uM	25uM	6.3uM	1.6uM	0.4uM	100uM	25uM	6.3uM	1.6uM	0.4uM
C	B	C	100uM	25uM	6.3uM	1.6uM	0.4uM	100uM	25uM	6.3uM	1.6uM	0.4uM

ROBONIK P2000 Eia reader

Wave length: 450 nm

Reference: 630 nm

	1	2	3	4	5	6	7	8	9	10	11	12
A	0	0.525	0.316	0.385	0.432	0.482	0.542	0.262	0.313	0.354	0.414	0.472
B	0	0.541	0.329	0.379	0.441	0.494	0.551	0.249	0.308	0.358	0.421	0.486
C	0	0.506	0.306	0.377	0.452	0.487	0.529	0.261	0.316	0.347	0.425	0.491
mean	0	0.524	0.317	0.3803	0.4417	0.4877	0.5407	0.2573	0.312	0.353	0.42	0.483
% viability			60.496	72.583	84.288	93.066	103.18	49.109	59.61	67.37	80.15	92.176

BPNO2 (3K)/A498

2	60.5

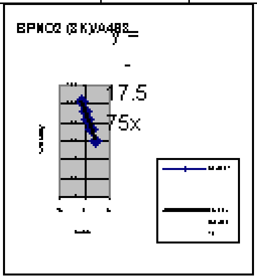

BTZD (3L)/A498

2	49.11

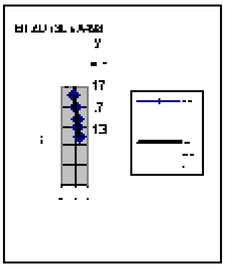

1.398	72.6
0.796	84.3
0.193	93.1
-0.41	103

1.3979	59.61
0.7959	67.37
0.1931	80.15
-0.409	92.18

Table S 8: Calculation of IC $_{50}$ of compounds **cisplatin** and **doxorubicin** against A498 cell line

	**Blank**	**CC**	**Sample No. Cis/A498**					**Sample No. DOX/A498**				
	**1**	**2**	**3**	**4**	**5**	**6**	**7**	**8**	**9**	**10**	**11**	**12**
A	B	C	100uM	25uM	6.3uM	1.6uM	0.4uM	100uM	25uM	6.3uM	1.6uM	0.4uM
B	B	C	100uM	25uM	6.3uM	1.6uM	0.4uM	100uM	25uM	6.3uM	1.6uM	0.4uM
C	B	C	100uM	25uM	6.3uM	1.6uM	0.4uM	100uM	25uM	6.3uM	1.6uM	0.4uM

ROBONIK P2000 Eia reader

Wave length: 450 nm
Reference: 630 nm

	**1**	**2**	**3**	**4**	**5**	**6**	**7**	**8**	**9**	**10**	**11**	**12**
A	0	0.518	0.192	0.242	0.286	0.324	0.371	0.129	0.178	0.228	0.272	0.324
B	0	0.488	0.202	0.251	0.292	0.331	0.365	0.138	0.193	0.236	0.279	0.308
C	0	0.469	0.195	0.258	0.294	0.336	0.382	0.125	0.184	0.234	0.265	0.319
mean	0	0.492	0.1963	0.2503	0.2907	0.3303	0.3727	0.1307	0.185	0.233	0.272	0.317
% viability			39.932	50.915	59.119	67.186	75.797	26.576	37.63	47.32	55.32	64.475

Cis/A498

2	39.9
1.398	50.9
0.796	59.1
0.193	67.2
-0.41	75.8

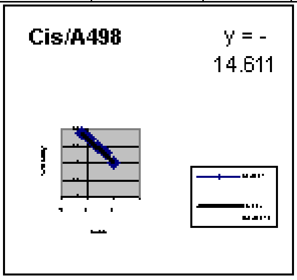

DOX/A498

2	26.58
1.3979	37.63
0.7959	47.32
0.1931	55.32
-0.409	64.47

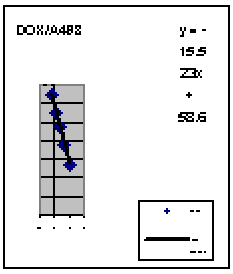

Topoisomerase enzyme assay

Topoisomerase I and II inhibitory activity

Compounds **3a-b, 3i-l**, cisplatin and doxorubicin was evaluated for topoisomerase Iα and topoisomerase IIβ inhibitory activity utilizing the human DNA topoisomerase Elisa kit. Standards and the tested compound were dissolved in sample diluent and 2 folds serial dilution was achieved. Biotin-conjugated antibody and avidin conjugated Horseradish Peroxidase (HRP-avidin) were diluted to 10 folds. 100 µL of each concentration of standard or test compound were added to each well. Incubate at 37 °C for 1 h. The liquid in each well was removed. 100 µL of Biotin-conjugated antibody solution was added to each well followed by incubation for 1 h at 37 °C. The microtiter plate was allowed to aspirate and washed 3 times. 100 µL of avidin conjugated Horseradish Peroxidase (HRP-avidin) solution was added to each well and the plate was incubated for 1 h at 37 °C. The plate was aspirated and washed 5 times. 90 µL of TMB substrate were added to each well and the plate was incubated for 30 min at 37 °C and protected from light. 50 µL stop solution was added. The absorbance was measured spectrophotometrically within 5 min at 450 nm**.**

Table S 9: Topoisomerase I/II inhibitory activity by compounds **3a-3b**, **3i-l**, cisplatin and doxorubicin.

Compound	Topoisomerase I IC_{50} in µM	Topoisomerase II IC_{50} µM
**3a**	14.50 ± 0.79	35.60 ± 1.94
**3b**	7.15 ± 0.39	31.10 ± 1.69
**3i**	**4.77 ± 0.26**	**15.00 ± 0.81**
**3j**	23.50 ± 1.28	39.80 ± 2.17
**3k**	36.60 ± 1.99	18.07 ± 1.02
**3l**	18.80 ± 1.02	14.30 ± 0.78
**Cisplatin**	5.71 ± 0.31	11.30 ± 0.62
**Doxorubicin**	3.36 ± 0.18	6.49 ± 0.35

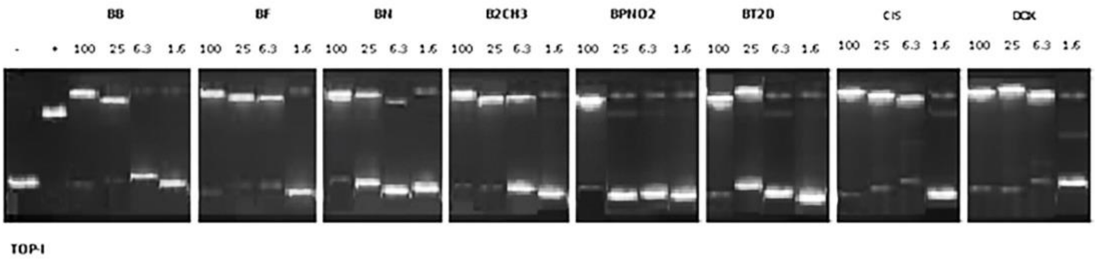

Figure S58: topoisomerase I inhibition induced by compounds **3a** (BB), **3b** (B2CH3), **3i** (BF), **3j** (BN), **3k** (BPNO2), **3l** (BTZD), **cisplatin** and **doxorubicin** (conc. in µg/mL)

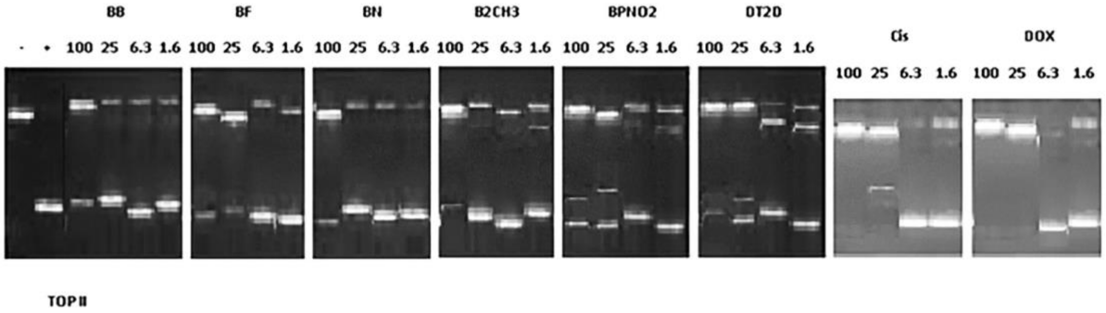

Figure S59: topoisomerase II inhibition induced by compounds **3a** (BB), **3b** (B2CH3), **3i** (BF), **3j** (BN), **3k** (BPNO2), **3l** (BTZD), **cisplatin** and **doxorubicin** (conc. in µg/mL)

Cell cycle analysis and detection of apoptosis

Annexin V-FITC apoptosis assay

Cell apoptosis were analyzed by the Annexin V-FITC Apoptosis Detection Kit (Bio Vision Research Products, USA). Thereafter, 1-5 x 105 cells were collected by centrifugation and resuspend in 500 µl of 1X Binding Buffer. Added 5 µl of Annexin V-FITC and 5 µl of propidium iodide (PI 50mg/ml, optional.). The cells incubated at room temperature for 5 min in the dark; Analyzed by Annexin V-FITC binding flow cytometric method (Ex = 488 nm; Em = 530 nm) using FITC signal detector (usually FL1) and PI staining by the phycoerythrin emission signal detector (usually FL2). For adherent cells, we gently trypsinized and washed cells once with serum-containing media before incubation with Annexin V-FITC (A.3-5).

Table S 10: the cell cycle analysis of Melanoma LOX IMVI cell treated with compound **3i** and **doxorubicin** against negative control.

Compound	%G0-G1	%S	%G2/M	%Pre-G1
**3i**/LOX IMVI	45.53	45.06	9.41	38.51
**Dox.** /LOX IMVI	37.85	56.21	5.94	46.29
**cont.** LOX IMVI	57.03	29.67	13.3	1.86

Table S11: the apoptosis assay of Melanoma LOX IMVI cell treated with IC_{50} concentration of compound **3i** and **doxorubicin** against negative control.

Compound	Apoptosis			Necrosis
	Total	Early	Late	
**3i/** LOX IMVI	38.51	3.03	24.17	11.31
**Dox. /** LOX IMVI	46.29	1.94	28.53	15.82
**cont.** LOX IMVI	1.86	0.55	0.16	1.15

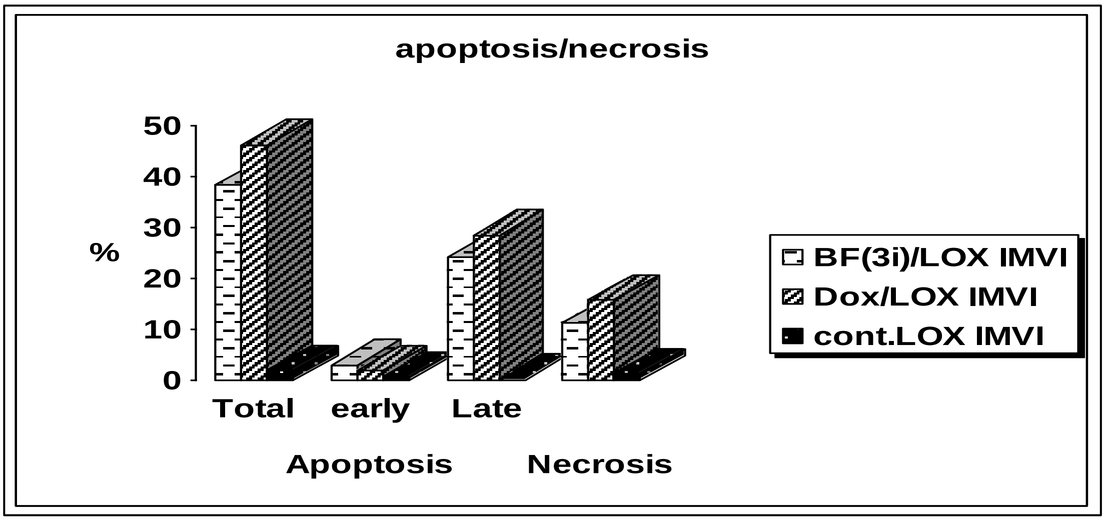

Figure S60: the apoptosis and necrosis assay of Melanoma LOX IMVI induced by IC_{50} concentration of compound **3i** and **doxorubicin** against negative control.

Effects of compound 3i on the protein expression level of Bax, caspases 3 activity and on PARP-1 inhibition

Gel electrophoresis and immuno-blot analysis of proteins (Western Blot)

(Burnette, 1981; Sambrook et al., 1989).

Principle:

Sodium dodecyl sulfate polyacrylamide gel electrophoresis (SDS-PAGE) is used to separate proteins based on their size. When coupled with western blotting (immunoblotting), both are typically used to determine the presence and/or relative abundance of a target protein in a sample containing a complex mixture of proteins. In this technique, total protein in each sample is loaded and electrophoretically separated by applying an electric current which allows the proteins to migrate through the gel matrix. In order for the proteins to migrate through the gel, they are first denatured and negatively charged by exposure to a detergent such as SDS. A molecular weight marker that produces bands of known size is used to help identifying proteins of interest. After the protein components have been sufficiently separated, they can be transferred to a polyvinylidene fluoride (PVDF) membrane by applying an electric current to the gel so that the proteins migrate out of the gel onto the membrane. For detection of a specific protein on the membrane, a primary antibody against that protein is added to form a protein-antibody complex followed by the addition of a secondary antibody that binds to the complex through its antibody side. The secondary antibody is typically linked to an enzyme that produces luminescence upon the reaction with its substrate. The amount of the luminescence, directly proportional to the amount of the protein that reacted with the antibody, is captured by Biorad Imager

Reagents preparation:

- Lysis buffer: 10mM Tris, 100mM NaCl, 25mM ethylenediamine tetra acetic acid (EDTA), 25mM Ethylene glycol bis(2-aminoethyl) tetra acetic acid (EGTA),0.1 Sodium dodecyl sulfate (SDS), % 1% (v/v) Triton X-100, 2% (v/v) NP-40 (pH 7.4), with 1:200 protease inhibitor cocktail (Sigma) and 1:300 phosphatase inhibitor cocktail Tablet (Roche).
- Protein marker (Thermo scientific)
- SDS Loading buffer (6X): 750 mM Tris-HCl (pH 6.8); 600 mM dithiothreitol (DTT); 12 % SDS; 0.012 % Bromophenol blue; 60 % glycerol
- Tris-Glycine SDS running buffer: 25 mM Tris; 192 mM glycine (electrophoresis grade) (pH 8.3 -8.4); 0.1% SDS.
- Tris-Glycine transfer buffer: 25 mM Tris; 192 mM glycine; 0.05% SDS; 15% methanol. Methanol was immediately added before the transfer.

- Tris Buffered Saline Tween (TBS-T): 10 mM Tris-HCl (pH 8.0); 150 mM NaCl; 0.1% Tween-20
- 5 % non-fat dry milk in TBS-T
- Primary antibodies for the proteins to be detected ....
- Secondary antibodies for the proteins to be detected
- ECL^{TM} western blotting detection chemiluminescent substrate (PerkinElmer, USA).

Procedure:

- The experiment was terminated by lysing the cells in cold lysis buffer. The cells were then immediately frozen at −20 °C for 1 h for further lysis, and collected by cell scraper and sonicated 2×10s, followed by centrifugation at 4000 rpm for 10 min under cooling.
- Total protein concentrations were determined colorimetrically in the supernatant using Bradford method before proceeding to the western blotting.
- **Western blotting**
- Equal amounts (20 µg) of protein samples were mixed and boiled with SDS Loading buffer for 10 min, allowed to cool on ice and then loaded into SDS-polyacrylamide gel and separated by Cleaver electrophoresis unit (Cleaver, UK), transferred onto polyvinylidene fluoride (PVDF) membranes (BioRad) for 30 min using a Semi-dry Electroblotter (Biorad, USA) at 2.5 A and 25 V for 30 min.
- The membrane was blocked with 5% nonfat dry milk in TBS-T for two hours at RT, in order to reduce non-specific protein interactions between the membrane and the antibody.
- The membrane was incubated overnight at 4°C with primary antibodies (Cell Signaling Technology) and β-actin (Sigma). The blots were then washed for three times (10 min each) with TBS-T.
- The membrane was then incubated with the corresponding horse radish peroxidase (HRP)- linked secondary antibodies (Dako) for another hour at room temperature, followed by washing for three times (10 min each) with TBS-T

- The chemiluminescent Western ECL substrate (Perkin Elmer, Waltham, MA) was applied to the blot according to the manufacturer's recommendation. Briefly, the membranes were incubated for 1 min with a mixture of equal volumes from ECL solution A and ECL solution B.
- The chemiluminescent signals were captured using a CCD camera-based imager (Chemi Doc imager, Biorad, USA), and the bands intensities were then measured by ImageLab (Biorad)
- Protein-sized markers were used in all gels to localize the gel transfer regions for specific proteins and determine the transfer efficiency.

Table S 12: Effects of compound **3i** on the protein expression level of Bax, caspases 3 activity and on PARP-1 inhibition

Compound	Caspace-3	Bax	PARP-1
	Optical density (OD)	OD	OD
3i/ LOX IMVI	3.14	4.13	0.87
Doxorubicin/ LOX IMVI	5.67	6.56	0.38
Control/ LOX IMVI	1	1	1

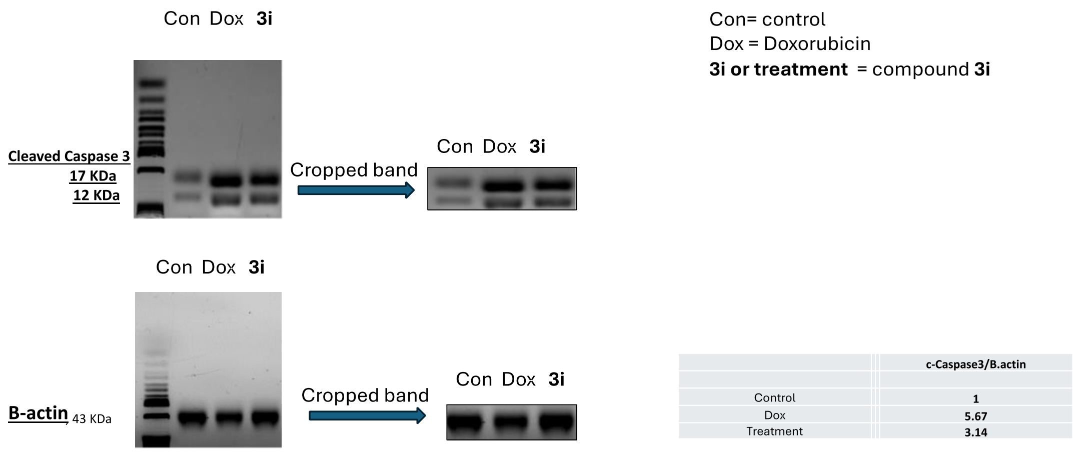

Figure For supp information: Effect of compound 3i on the expression Cleaved Caspase-3

Figure S61: Effects of compound **3i** (BF3) on the protein expression level of Casase 3,in LOX IMVI cell line.

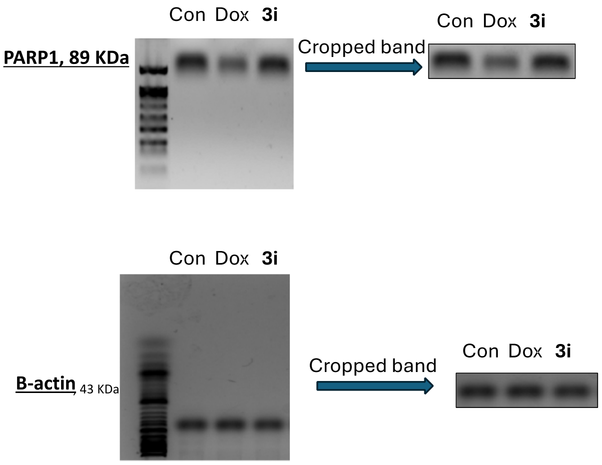

Con= control
Dox = Doxorubicin
3i or treatment = compound **3i**

	PARP1/B.actin
Control	1
Dox	0.38
Treatment	0.87

Figure For supp information: Effect of compound 3i on the expression PARP1.

Figure S62: Effects of compound **3i** (BF3) on the protein expression level of PARP-1 activity in LOX IMVI cell line.

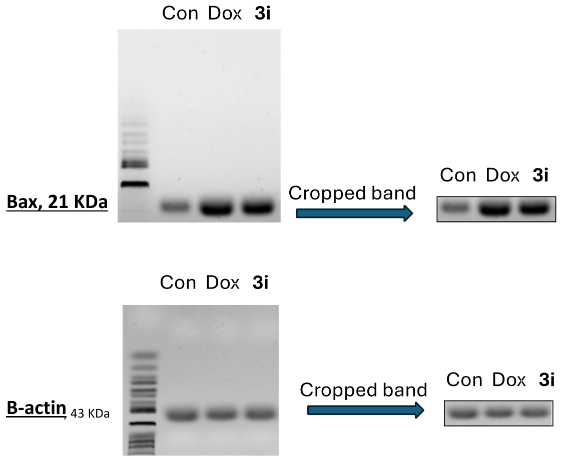

Con= control
Dox = Doxorubicin
3i or treatment = compound **3i**

	Bax/B.actin
Control	1
Dox	6.56
Treatment	4.13

Figure For supp information: Effect of compound 3i on the expression Bax

Figure S63: Effects of compound **3i** (BF3) on the protein expression level of Bax, activity in LOX IMVI cell line.

Docking studies

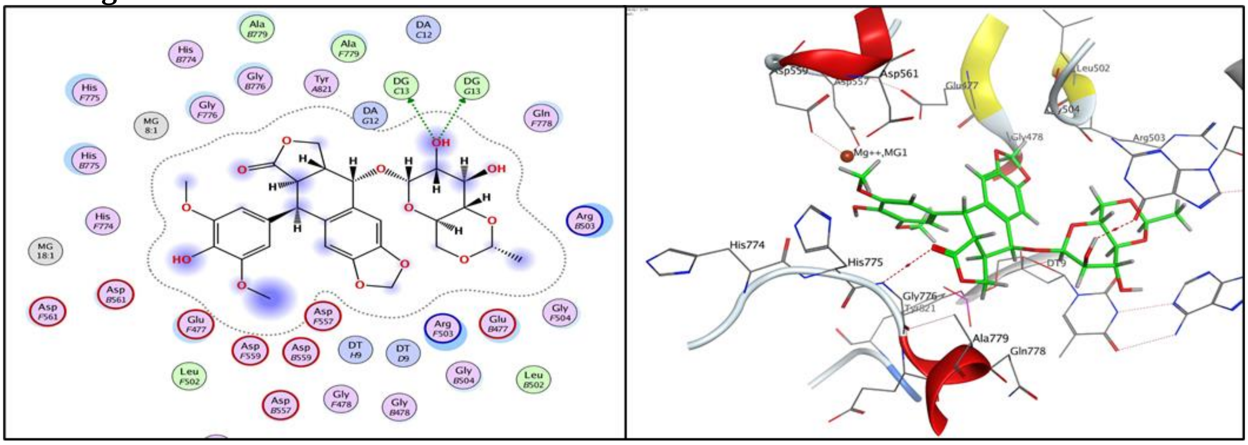

Figure S64: 2D and 3D illustration of **etoposide** docked into the active site of topoisomerase II enzyme (PDB: 3QX3).
